# Unlocking New Topologies in Zr-Based Metal–Organic Frameworks by Combining Linker Flexibility and Building Block Disorder

**DOI:** 10.1021/jacs.2c13731

**Published:** 2023-04-26

**Authors:** Charlotte Koschnick, Maxwell W. Terban, Ruggero Frison, Martin Etter, Felix A. Böhm, Davide M. Proserpio, Simon Krause, Robert E. Dinnebier, Stefano Canossa, Bettina V. Lotsch

**Affiliations:** † Max Planck Institute for Solid State Research, Heisenbergstraße 1, Stuttgart 70569, Germany; ‡ Department of Chemistry, University of Munich, Butenandtstraße 5-13, Munich 81377, Germany; § Physik-Institut, University of Zurich, Winterthurerstrasse 190, Zurich CH-8057, Switzerland; ∥ Deutsches Elektronen-Synchrotron (DESY), Notkestraße 85, Hamburg 22607, Germany; ⊥ Dipartimento di Chimica, Università Degli Studi di Milano, Via Golgi 19, Milano 20133, Italy

## Abstract

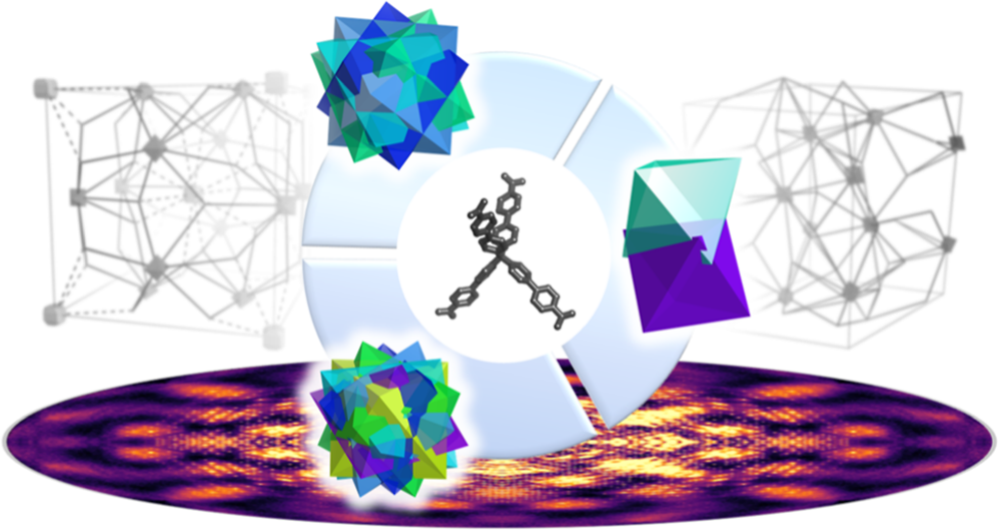

The outstanding diversity of Zr-based frameworks is inherently linked to the variable coordination geometry of Zr-oxo clusters and the conformational flexibility of the linker, both of which allow for different framework topologies based on the same linker–cluster combination. In addition, intrinsic structural disorder provides a largely unexplored handle to further expand the accessibility of novel metal–organic framework (MOF) structures that can be formed. In this work, we report the concomitant synthesis of three topologically different MOFs based on the same M_6_O_4_(OH)_4_ clusters (M = Zr or Hf) and methane-tetrakis(*p*-biphenyl-carboxylate) (MTBC) linkers. Two novel structural models are presented based on single-crystal diffraction analysis, namely, cubic c-(4,12)MTBC-M_6_ and trigonal tr-(4,12)MTBC-M_6_, which comprise 12-coordinated clusters and 4-coordinated tetrahedral linkers. Notably, the cubic phase features a new architecture based on orientational cluster disorder, which is essential for its formation and has been analyzed by a combination of average structure refinements and diffuse scattering analysis from both powder and single-crystal X-ray diffraction data. The trigonal phase also features structure disorder, although involving both linkers and secondary building units. In both phases, remarkable geometrical distortion of the MTBC linkers illustrates how linker flexibility is also essential for their formation and expands the range of achievable topologies in Zr-based MOFs and its analogues.

## Introduction

With more than 2000 publications to date, Zr-based metal–organic frameworks (MOFs) are among the most intensively investigated classes of MOFs for a variety of applications including gas storage and separation, catalysis, and drug delivery.^1^ When combined with carboxylate-terminated organic linkers, the strong Zr–O bonds impart high chemical and thermal stability to these MOFs.^2,3^ Hexanuclear Zr_6_O_4_(OH)_4_ secondary building units (SBUs) are by far the most commonly observed and can accommodate up to 12 linker-connected carboxylate groups, thus reaching charge neutrality.^4,5^ Zr_6_O_4_(OH)_4_ coordination sites not occupied by linker carboxylate groups often host reactive and displaceable OH/H_2_O pairs, which are key for post-synthetic modifications to introduce functional groups onto the cluster.^6−8^ Given their versatile coordination capacity, combined with the possibility of capping some of these sites with hydroxy anions or monocarboxylates, Zr clusters can lead to a wide range of MOF topologies depending on the geometric properties of the selected linkers and synthesis conditions.^9−11^ Combination of Zr_6_O_4_(OH)_4_ SBUs with tetrakis(4-carboxyphenyl)porphyrin linkers, for example, leads to five different MOF networks: **she** in PCN-224,^12^ averaged **ftw** in disordered PCN-224 (dPCN-224),^13^**scu** in NU-902,^14^**csq** in PCN-222/MOF-545,^15,16^**sqc** in PCN-225,^17^ and **shp** in PCN-223.^18^ In contrast, formation of **ith** and **flu** type networks requires tetrahedral linkers such as methanetetrabenzoic acid (MTB) yielding MOF-812 and MOF-841, respectively.^19^ The elongated methane-tetrakis(*p*-biphenylcarboxylate) (MTBC) linker yields the isoreticular MOF PCN-521 with the same **flu** network as MOF-841, but with larger cavities.^20^

The topological diversity of Zr-based MOFs is generally limited to combinations of linkers and clusters whose geometries are compatible with the formation of two- or three-dimensional periodic architectures. In this regard, linker flexibility can be key to expanding the number of topologies by mitigating moderate geometrical frustration of the MOF;^21^ that is, when the ideal geometry of the building blocks does not allow the formation of extended structures. In addition to linker flexibility, orientational disorder of the building blocks can also afford topologies otherwise incompatible due to geometrical frustration by making the geometry of the disordered species less relevant for the framework connectivity. This can occur during the formation of a framework, when some of its inorganic nodes accept linker coordination with a geometry that is not compatible with a unique orientation of the metal cluster, thus resulting in multiple cluster orientations bearing analogous—if not equivalent—energies.^13,18,22,23^ It is worthwhile keeping in mind that the structural complexity of such disordered MOFs can complicate the interpretation of crystallographic data and even result in misleading structural interpretation.^24^ For example, it was recently shown that cubic Zr_8_O_6_ clusters appearing via structure refinements from single-crystal X-ray diffraction (SCXRD) of PCN-221 actually resulted from the superposition of disordered Zr_6_O_4_(OH)_4_ SBUs.^13^ Analogous octanuclear clusters were also reported in Zr-MTBC,^25^ a MOF based on 4-connected MTBC linkers and a combination of 12-connected cubic Zr_8_O_8_(OH)_4_ and octahedral Zr_6_O_4_(OH)_4_ SBUs. Here, we show that observation of these cubic clusters is, similar to dPCN-224, a marker for orientational disorder, which allows the formation of this particular framework topology. We thus present a new model to describe the structure, herein abbreviated as c-(4,12)MTBC-Zr_6_, based on disordered Zr_6_O_4_(OH)_4_ SBUs, which shares analogous unit cell parameters and **buh** topology with Zr-MTBC. We further describe the complex variety of other phases obtained by the combination of octahedral clusters of the type M_6_O_4_(OH)_4_ (M = Zr, Hf) and MTBC linkers, which include an unreported MOF with trigonal symmetry, tr-(4,12)MTBC-M_6_, featuring a complex disordered structure. By these means, we highlight a new approach to investigate, resolve, and structurally define disorder as a new design tool for Zr-and Hf-based MOFs toward unexpected structural motifs. This poorly explored phenomenon and the overlooked degrees of freedom are likely present in many other reported structures.^26^ In addition, our results present disorder as a strategy to circumvent structural frustration that may lead to novel properties in these frameworks.

## Results and Discussion

### Crystal Structure and Disorder in c-(4,12)MTBC-Zr_6_

**Figure 1:**
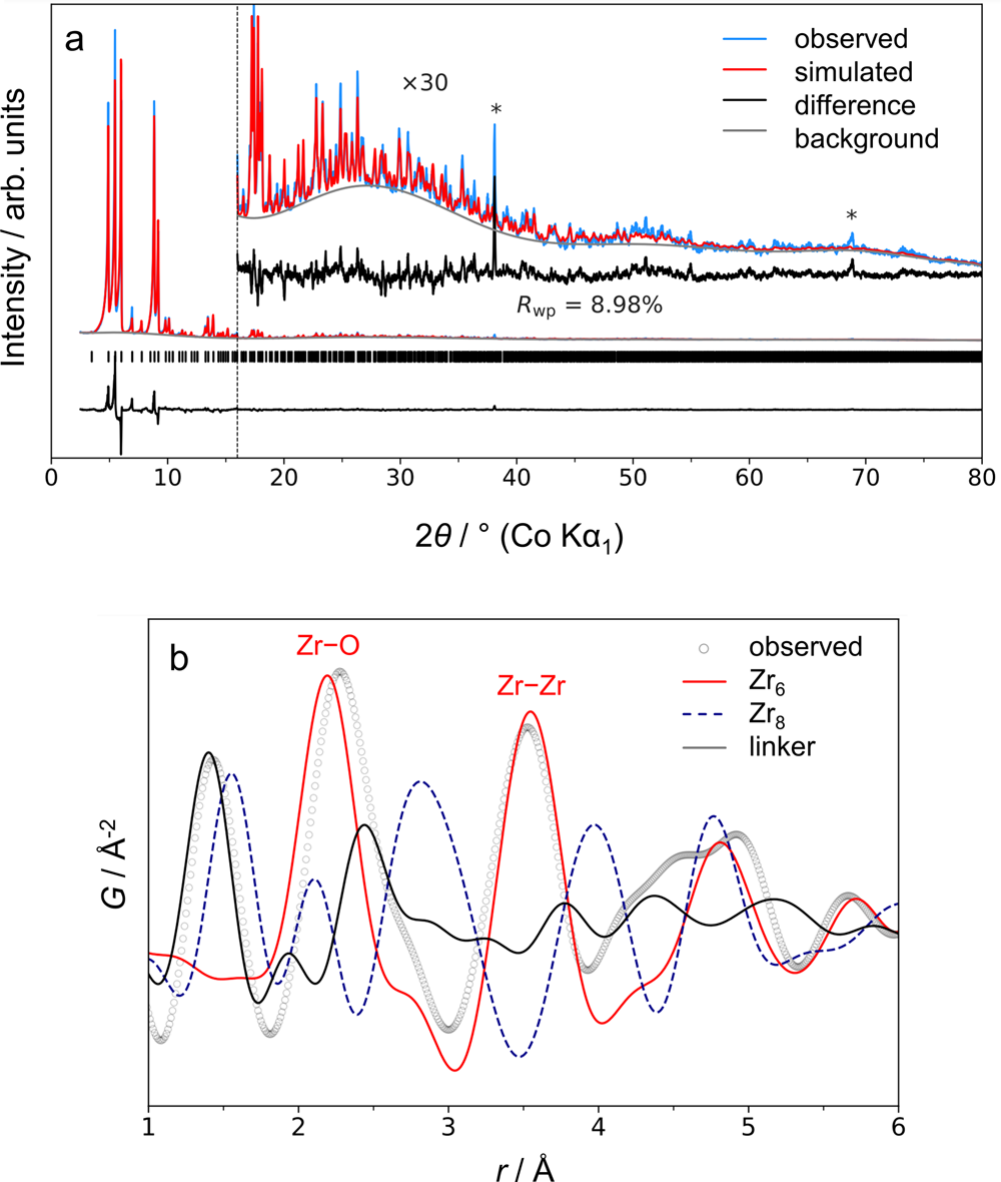
Rietveld refinement of the experimental c-(4,12)MTBC-Zr_6_ PXRD pattern (Co Kα_1_) using the published model of Zr-MTBC (a)^25^ and experimental PDF (Mo Kα_1_ radiation source, *Q*_max_ = 14.5 Å^–1^) of c-(4,12)MTBC-Zr_6_ compared to the simulated PDFs of Zr_6_ and Zr_8_ clusters, and the MTBC linker from the published Zr-MTBC structure (b). An artefact peak in the subplot of the PXRD profile is due to sample environment and marked with an asterisk.

Solvothermal synthesis of c-(4,12)MTBC-Zr_6_ was achieved by reacting ZrCl_4_ and MTBC in diethylformamide (DEF) with benzoic acid as modulator, following a reported procedure (Supporting Information Section S1.2).^25^ Under these conditions, crystals with sizes in the range of 2–5 μm were obtained as truncated octahedra (Figures S2.2.1 and S2.2.2). The experimental powder X-ray diffraction (PXRD) pattern of the product agrees well with the simulated pattern of the published Zr-MTBC structure, which was reported with both cubic Zr_8_O_6_ and octahedral Zr_6_O_4_(OH)_4_ SBUs (Figure 1a).^25^ To test for the presence of Zr_8_ clusters, we analyzed the Zr-MTBC crystals with pair distribution function (PDF) analysis, which suggests the presence of Zr_6_O_4_(OH)_4_ SBUs only (Figure 1b). This draws parallels to porphyrinic MOF PCN-221 previously published with Zr_8_O_6_ clusters, which according to our recent study is more correctly described by the new structure model *d*PCN-224 with disordered Zr_6_O_4_(OH)_4_ SBUs.^13^

**Figure 2:**
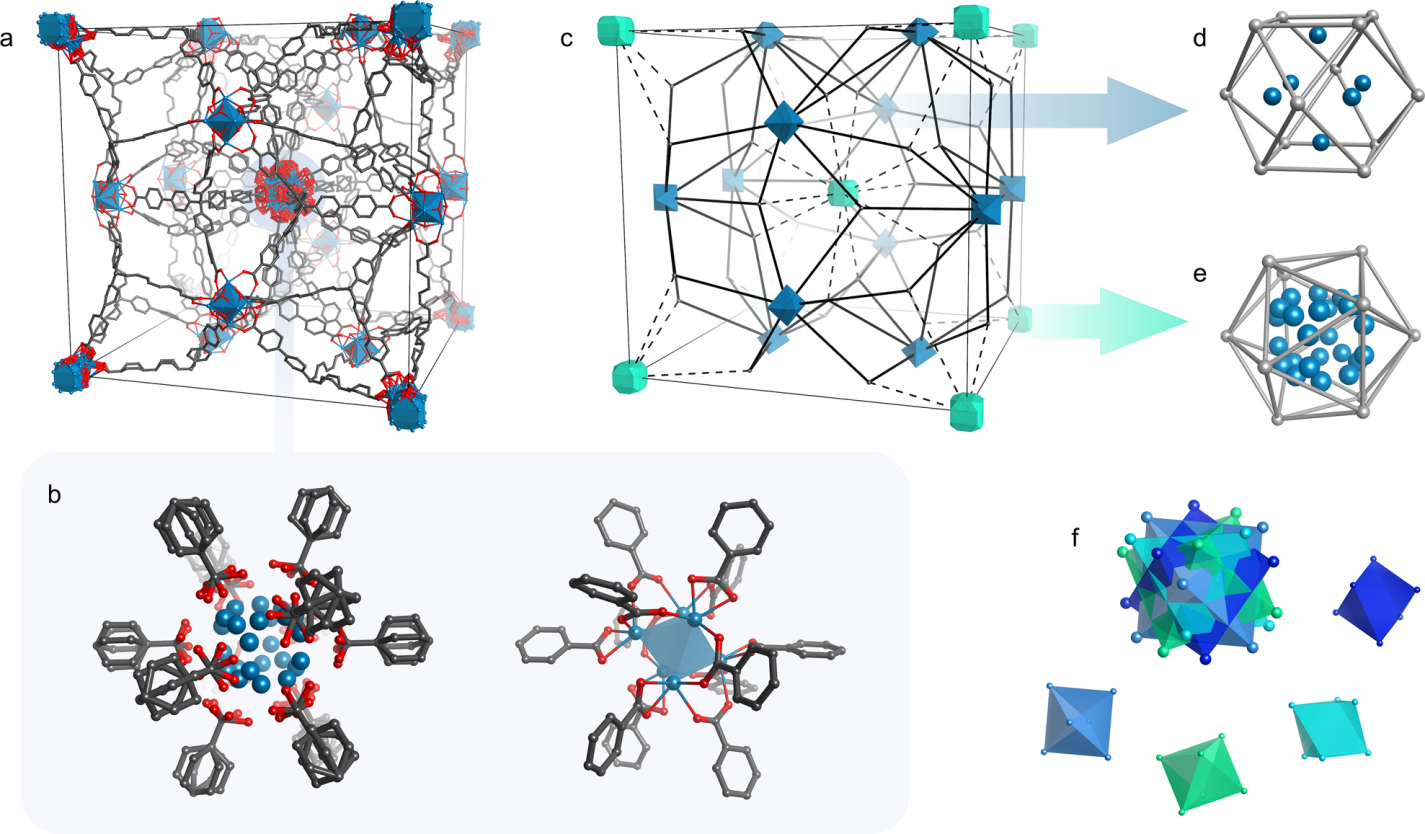
Crystal structure of c-(4,12)MTBC-Zr_6_. The complete unit cell (a) is displayed highlighting the modeled average disordered cluster site next to one of its four possible local variants (b). Next to the topological view (c), the ordered and disordered cluster sites (d,e) are simplified by showing Zr atoms in blue and the surrounding linker carboxylate carbons in silver. The superposition of four Zr_6_ octahedra in the disordered cluster is also highlighted by marking octahedra in different colors (f). Only the fraction of linkers contained in the unit cell is displayed for the sake of clarity.

We next optimized the synthesis for increasing the crystal size. By conducting the reaction without stirring to favor heterogeneous over homogeneous nucleation (as supersaturation decreases in proximity of nucleation sites when diffusion layers are not eliminated by mixing), we obtained single crystals of up to 200 μm in size with the same truncated octahedral (**TO**) morphology (Figure S3.2.1a). Structure refinements using SCXRD data revealed a cubic framework where two distinct cluster sites are present, both accepting 12 carboxylate groups of 12 linkers exhibiting a moderate distortion (100–123°) from their ideal tetrahedral geometry (109.5°). As far as the SBUs are concerned, c-(4,12)MTBC-Zr_6_ features two crystallographically distinct sites hosting Zr_6_O_4_(OH)_4_ 12-connected SBUs: one perfectly ordered and one where they are disordered in four alternative positions (Figure 2a–c). Sites which accommodate a single and ordered cluster orientation possess a slightly distorted cuboctahedral coordination environment (Figure 2d), which is similar to the Zr_6_O_4_(OH)_4_ environment found in UiO-66.^27^ The geometrical distortion for the other site is, however, severe enough to cause multiple degenerate orientations of the cluster (Figure 2e,f). Here, metal-nodes are approached by 12 linker carboxylate groups with a distorted icosahedral geometry, which significantly deviates from the ideal coordination environment of the cluster. Thus, Zr_6_O_4_(OH)_4_ SBUs adopt one of four possible orientations, resulting in a framework with well-defined linker positions, but disordered, 12-fold coordinated clusters. The coordination environment of these clusters is essentially identical to the one found in the isoreticular framework NPF-200.^28^ Its structure features the same topology and space group symmetry as that of c-(4,12)MTBC-Zr_6_ and, in the sites where we report disordered Zr_6_O_4_(OH)_4_ octahedra, cubic Zr_8_(μ_2_-O(H))_12_ clusters were assigned to the observed electron densities. While this was proposed as second evidence of the existence of cubic Zr_8_-type clusters after their first claim in PCN221, also in this case, refinements were based on diffraction data of relatively poor quality (reported *R*_int_ ≈ 11% at 1.0 Å resolution). This might have hampered the observation of electron density residues consistent with the presence of disordered Zr_6_O_4_(OH)_4_.

**Figure 3:**
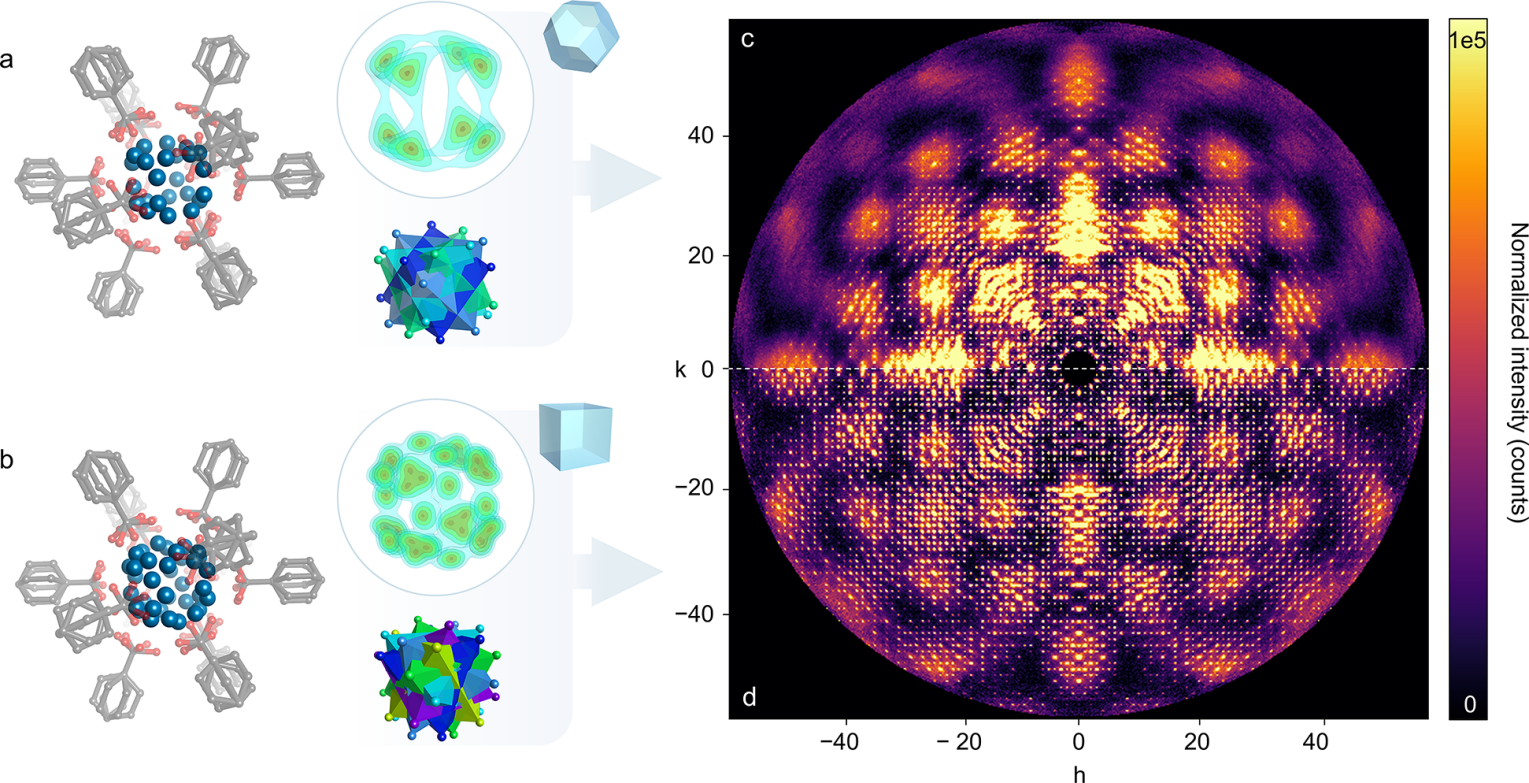
The two obtained variants of c-(4,12)MTBC-Zr_6_ as truncated octahedra (a) and cubes (b) are displayed by highlighting the differences in electron density maps and the crystallographic model of their disordered cluster sites. These relate to marked differences in crystallinity as can be seen from the reciprocal space reconstruction of the respective *hk*0 planes (c,d). Intensities are normalized to the total number of counts. Electron density isosurface levels (light blue to dark red) correspond to 1.7, 2.4, 2.8, 3.1, and 3.25 e^–^/Å^3^ (a) and 2.0, 3.0, 4.6, 6.0, and 6.5 e^–^/Å^3^ (b).

The disorder in c-(4,12)MTBC-Zr_6_ leaves characteristic diffuse scattering visible in reciprocal space reconstructions from SCXRD data. As shown in Figure 3c, Bragg reflections reach a relatively low diffraction angle, while broad cloud-like features become more prominent with increasing distance from the origin. The rather weak diffraction of this MOF was significantly improved by changing the synthetic conditions. By decreasing the concentration of ZrCl_4_ and the MTBC linker in the reaction mixture and increasing the reaction time, the same MOF structure was obtained but with a cubic, instead of **TO**, crystal morphology (Figure S3.2.1c). While the crystal size remained similar (≈200 μm), cube-shaped c-(4,12)MTBC-Zr_6_ features a significantly different orientational disorder of the cluster, as additional electron density maxima can be observed from difference electron density maps. These were successfully modeled as additional metal positions consistent with an overall superposition of six Zr_6_ octahedra instead of four (Figure 3a compared to Figure 3b). The occupancies of all metal centers were then refined to achieve an overall full occupancy of the metal cluster on this site. (See the Supporting Information for further details). A deconstruction of their overlapped structure is displayed in the Supporting Information file, Figure S6.2.12. Concomitantly, these cube-shaped crystals show, compared to their **TO** analogues, sharp Bragg peaks up to considerably higher spatial frequencies (linked to higher diffraction angle in the experiments) due to a higher framework periodicity (Figure 3c compared to Figure 3d). This demonstrates that by changing the synthetic conditions, it is possible to achieve higher crystallinity and affect the type of cluster disorder. In the current reticular chemistry landscape, this is a rarely documented example of enhancement of tunable structural disorder through simple manipulation of the synthetic procedure evidenced by single-crystal diffraction data.^29^

### Single Crystal Diffuse Scattering Analysis

**Figure 4:**
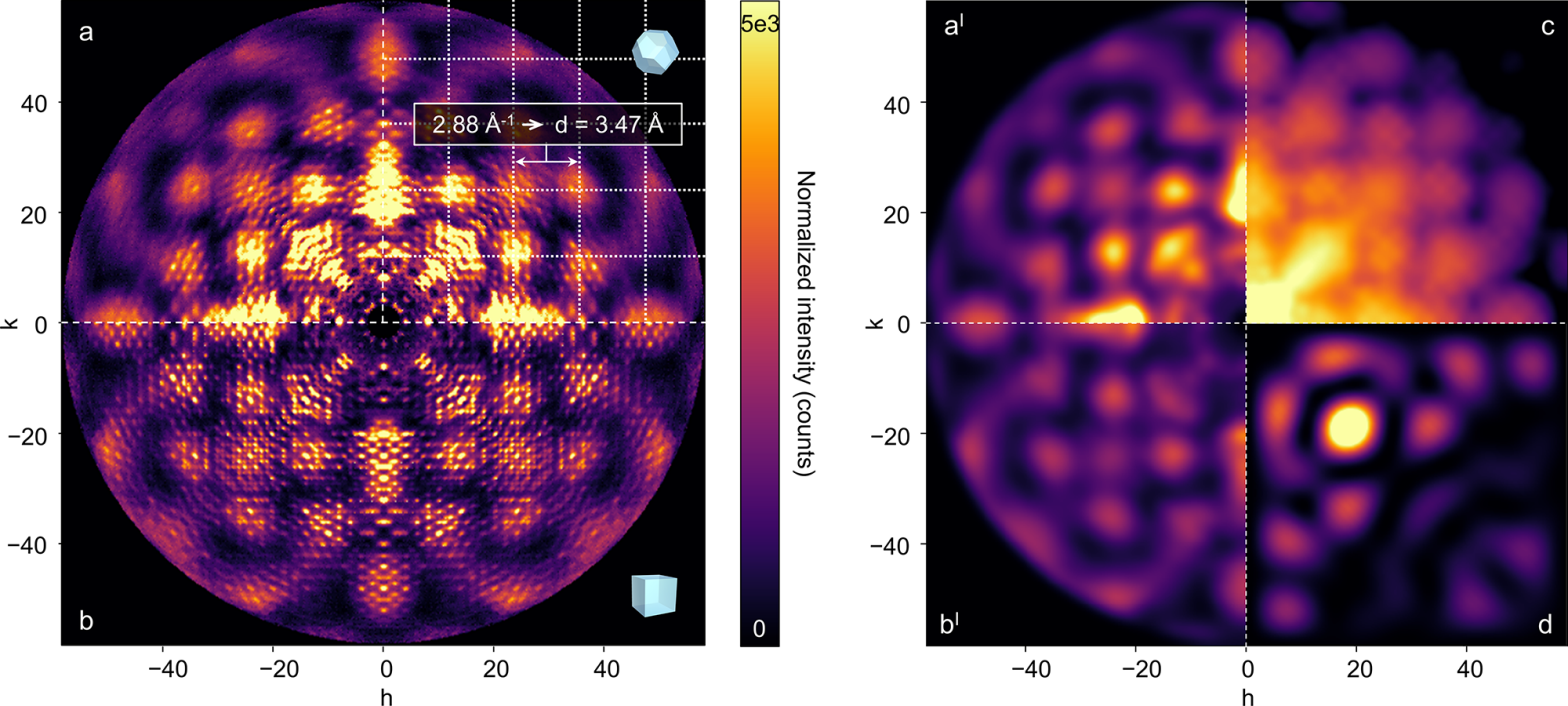
Diffuse scattering from the *hk*0 plane of c-(4,12)MTBC-Zr_6_ with **TO** and cubic morphology (a,b, respectively) obtained by removing Bragg reflections. On the right, corresponding cloud-like features extracted by using a Gaussian frequency filter on the experimental diffuse scattering without changing the intensity scale (a^I^,b^I^), calculated diffraction patterns of a single unit cell (c, logarithmic scale to facilitate comparison) and diffuse scattering from a simulated c-(4,12)MTBC-Zr_6_ crystal containing a random distribution of disordered cluster positions (d). Arbitrary intensity scales are used for (c,d) to facilitate comparison.

While distinct morphologies and types of cluster disorder in c-(4,12)MTBC-Zr_6_ are linked to synthetic conditions, some diffuse scattering characteristics are systematically found in both **TO** and cube-shaped crystals (Figure 4a,b). First, these intensities are generally arranged in clouds forming a somewhat square grid with spacing approaching the equivalent of 3.5 Å (Figure 4a), which is the Zr–Zr distance found in the ordered Zr_6_O_4_(OH)_4_ SBUs from our refinements (3.5001(9) Å). Furthermore, the diffuse-to-Bragg intensity ratio visibly increases from the origin of reciprocal space outward, and, in the case of **TO** crystals, diffuse scattering remains the only type of intensity present until the edge of the sampled reciprocal space. As no notable intensity maxima are present between Bragg reflections, the overall picture points toward a major displacive disorder contribution to the observed diffuse intensities,^30,31^ without any indication of missing cluster or missing linker defects—which might be present in low, undetectable amounts. To understand the origin of the observed diffuse intensities, we calculated the diffraction pattern of c-(4,12)MTBC-Zr_6_ model crystals featuring only random cluster disorder, based on the average structure refined from **TO** crystals (Figure 4d). These were produced *via* Monte Carlo simulations by creating a supercell where each disordered cluster site hosts a specific cluster orientation that does not depend on which orientation is present in the neighboring equivalent sites (Supporting Information Section S7.2). The computed diffraction pattern shows diffuse intensity clouds arranged with a significantly different distribution with respect to the experimental patterns (Figures 4d versus 4aI,4bI) as they are mostly absent along the main axes (*h*00 and 0*k*0 in the *hk*0 plane). This is because none of the different cluster orientations in the disordered sites includes Zr–Zr correlations along the main crystal directions (Comparison of Figures S7.1.11 and S7.1.12). The ordered clusters, on the other hand, contain such correlations, and a simulated diffraction pattern from a single unit cell based exclusively on the ordered structure (no disordered clusters) shows an overall good match with the diffuse intensity distributions of the experimental patterns (Figure 4c). This shows that, in the latter, the distribution of diffuse features is mostly defined by the unit cell form factor and not by one of the disordered clusters. While cluster disorder is present as evidenced by the average structure, the diffuse scattering features associated to the distribution of cluster orientations across the framework cannot be distinguished in the experimental data.

**Figure 5:**
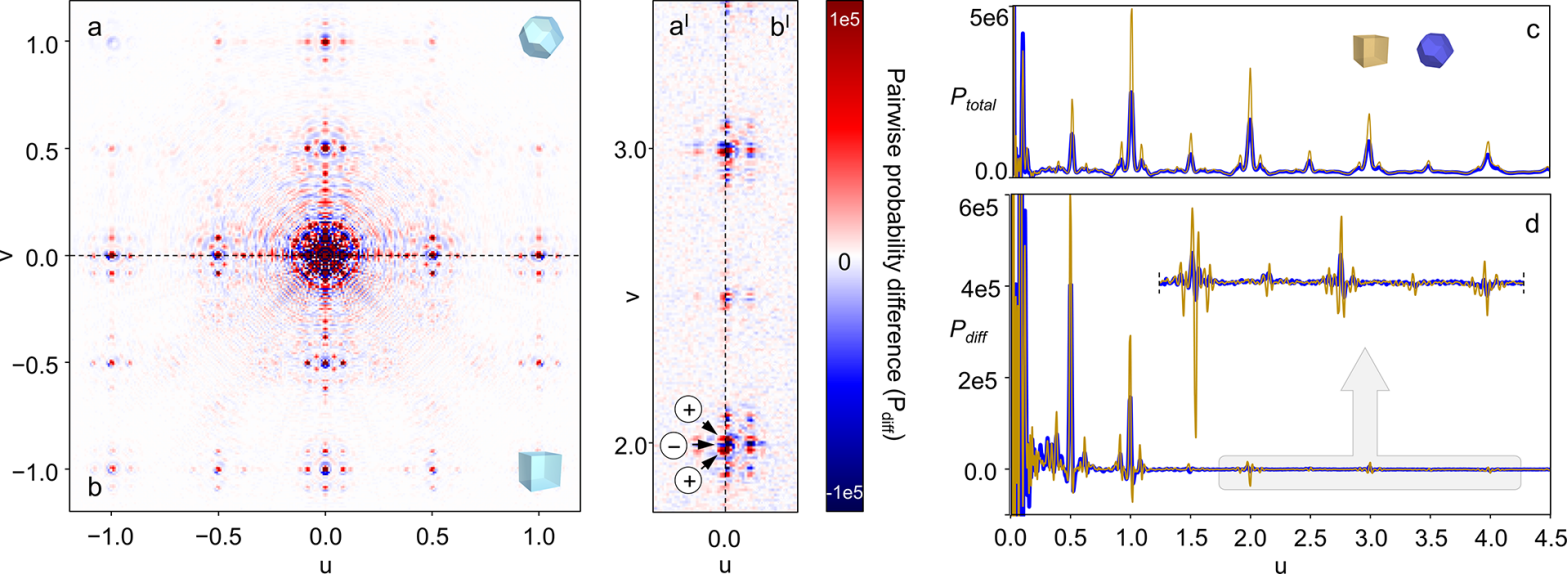
Plot of the *uv*0 plane from the 3DΔPDF of **TO** (a,a^I^) and cube-shaped (b,b^I^) c-(4,12)MTBC-Zr_6_ crystals and probability density scan along *u*00 extracted from their total 3D PDF (c) and 3DΔPDF (d) up to 4.5 unit cells distance.

Additional information on the local structure of c-(4,12)MTBC-Zr_6_ can be found in the three-dimensional (3D) PDF (3DPDF) and in the 3D delta PDF (3DΔPDF).^32^ The former can be obtained by direct Fourier transform (FT) of the total experimental intensities obtained from single-crystal diffraction measurements, the latter by first removing the Bragg reflections from the reconstructed 3D reciprocal space and then by applying FT (Figure 5a–b,aI–bI; Supporting Information Section S7.1).^33^ The result is a 3D map displaying the difference between the interatomic correlations in the real structure of c-(4,12)MTBC-Zr_6_ and those belonging to the average structure—subtracted by the Bragg peaks’ removal. In these maps from both cube-shaped and **TO** crystals, the absence of isolated negative correlation peaks within 1 unit cell distance indicates that no vacancies such as missing linkers or clusters are detectable. Indeed, vacancies would produce characteristic negative maxima without surrounding positive signals of comparable intensity. On the contrary, the 3DΔPDF is dominated by positive correlation peaks with proximal negative oscillations, which signal the presence of displacive disorder. The positive components of the 3DΔPDF share similar positions, at short distances, with the total 3D PDF (comparison of Figure 5c with 5d). Since the latter is dominated by the average structure, this supports further that the information contained in the extracted diffuse scattering and transferred to the 3DΔPDF relates to framework distortion rather than to the orientation of the disordered clusters. Given the qualitative similarity of the patterns from cube-shaped and **TO** crystals, this conclusion applies to both types of products.

Overall, the available evidence indicates that the observed diffuse scattering from c-(4,12)MTBC-Zr_6_ crystals originates from correlated framework distortion. Its origin can be reasonably attributed to the alternative orientations of the disordered clusters and the resulting adaptation of surrounding linkers, which creates a static “wave” of deformation across the framework. This interpretation is consistent with the alternation of positive and negative correlation peaks in the 3DΔPDF,^29,32,34−36^ indicating that displacive disorder is the main deviation from periodicity in both cube-shaped and **TO** crystals. As for the link between their differences in morphology, defect structure, and diffuse intensities, it is not possible to define a rationale that is reliably supported by experimental data. There is, however, a clear link between lower reactants’ concentration in the synthesis, faster growth of {111} crystal facets with respect to {100} ones (resulting in a cubic morphology), and an overall higher crystallinity. The latter is also consistent with the isotropic Zr atoms’ mean-square displacements (crystallographic U_eq_ parameters), which range from 0.0505(1) to 0.0526(1) Å^2^ in cube-shaped crystals and from 0.1135(2) to 0.1318(3) Å^2^ in **TO** crystals.

### Multiphase Behavior of MTBC-Based Zr MOFs: tr-(4,12)MTBC-Zr_6_

The optimized synthesis of c-(4,12)MTBC-Zr_6_ single crystals did not yield a phase-pure product (Supporting Information Section S1.4–1.5) but instead resulted in phase mixtures where two additional compounds were found as elongated octahedral crystals and hexagonal platelets. The first was identified by SCXRD as the tetragonal MOF PCN-521 (4,8)-**flu**,^20^ while the latter revealed another yet unreported MTBC-Zr_6_ framework with the same (4,12)MTBC-Zr_6_ connectivity, but with a trigonal symmetry (space group *P*3̅*c*1) and unprecedented topology: tr-(4,12)MTBC-Zr_6_. Compared to the cubic trinodal (4,12)-**buh** of c-(4,12)MTBC-Zr_6_, the trigonal phase features a new unreported tetranodal (4,12) net (Supporting Information Section S8).

**Figure 6:**
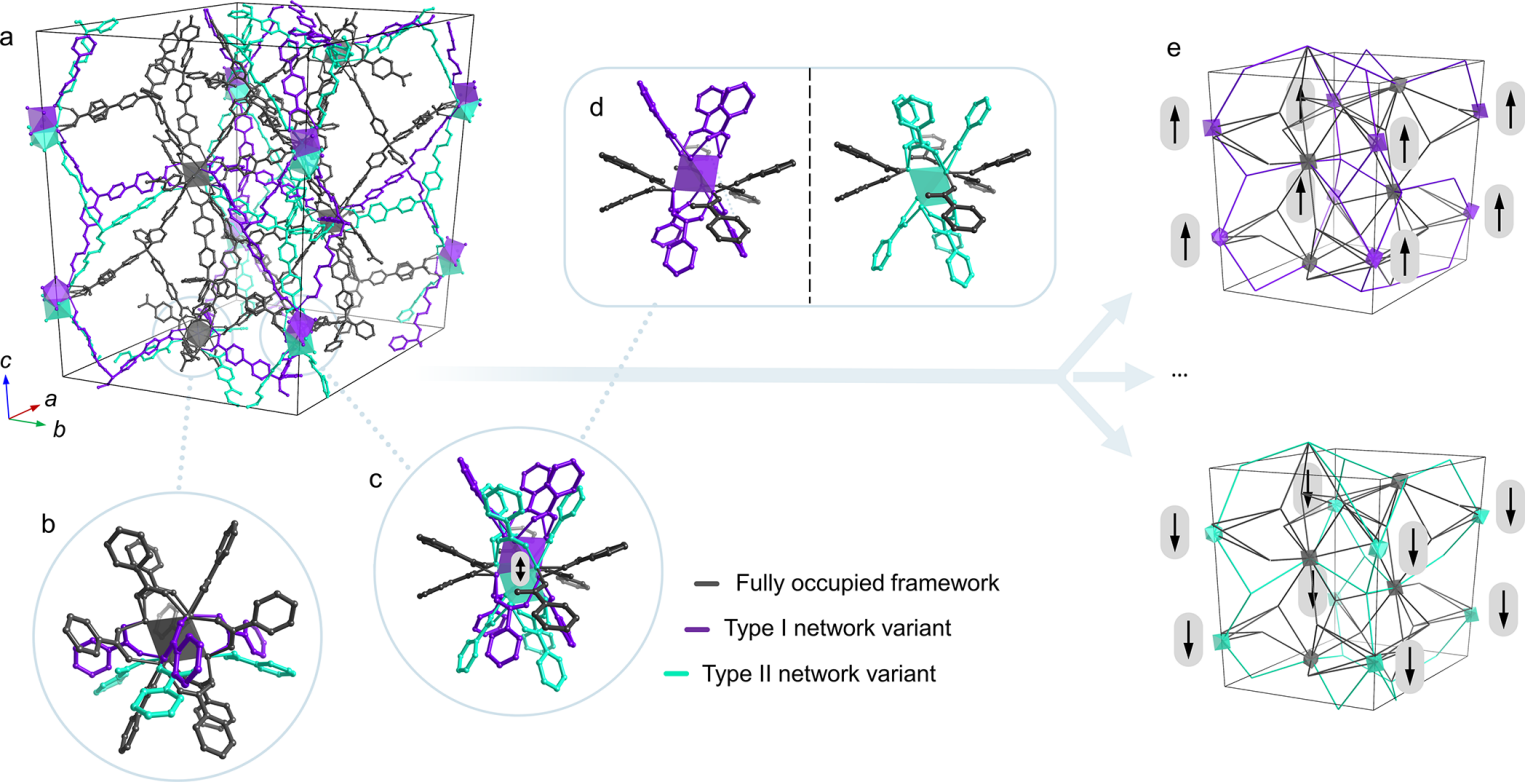
Characteristics of the complex topology in the tr-(4,12)MTBC-Zr_6_ structure. The complete unit cell (a) is shown with the fully occupied clusters and linkers in gray (b) and alternative framework fractions in violet and turquoise. Clusters lying on the edges of the unit cell are disordered in two alternative positions (c), either above or below the (1/4 0 0) site (d). Of all possible periodic or aperiodic networks, those resulting from all “upshifted” or all “downshifted” clusters are presented (e).

The trigonal structure features a complex combination of mutually exclusive networks originating from correlated disorder of the building blocks (Figure 6). In particular, while some clusters are not disordered and fully occupied (gray in Figure 6), one-third of the clusters lie in two alternative positions: either above or below the crystallographic sites (0, 0, 1/4) and (0, 0, 3/4) and shifted from them by 1.027(3) Å (Figure 6c,d). Accordingly, three sets of highly distorted linkers (tetrahedral angle: 89–125°) can be identified depending on whether they are linked to the upshifted or downshifted Zr_6_ clusters (violet and turquoise, respectively, in Figure 6). We dismissed the possibility of wrong symmetry assignment by repeating the data reduction and structure solution using the P1 space group, which showed the same kind of framework disorder also when including inversion twinning in the model. Given the continuous alternation of clusters and linkers along the unique axis *c* and the general absence of vacancy defects suggested by our refinements, it can be reasonably assumed that a single column of building blocks along *c* comprises mostly upshifted or downshifted building blocks, thus making the structure disorder along *c* highly correlated—ideally affording periodicity. On the other hand, the framework structure is compatible with neighboring columns of building blocks being shifted differently with respect to one another, thereby creating aperiodicity in the *ab* plane of the crystals. The resulting aperiodic modular structure^37^ represents a rare case of multivariate framework architecture,^38^ akin to the intrinsically aperiodic Truchet-tile structure of the recently reported TRUMOF-1.^39^ This agrees with diffuse scattering features present in reciprocal space reconstructions, which evidence extra reflections and diffuse streaks within the *hk*0 plane, while only sharp intensities are present along the orthogonal direction (Figure S6.2.10). Nonetheless, the latter appears to be inconsistent with a well-defined value of the *c*-axis and suggests the presence of a complex structure modulation whose analysis is beyond the scope of this study. Interestingly, the linkers belonging to the framework’s upshifted variant bind the ordered clusters with a typical bridging coordination, where each oxygen binds to a different Zr center. Conversely, the other fraction of disordered linkers—downshifted variant—bind these clusters with a monodentate η^1^-coordination (Figure 6b). While a smaller occupancy could be expected for these last linkers due to their weaker bonding to the clusters, it was not possible to refine their occupancies separately since the two parts are crystallographically generating one another by symmetry. On the other hand, if their occurrence is indeed less energetically favorable, one would expect the formation of frameworks where only the upshifted (or only downshifted) configuration is present, resulting in an ordered structure (Figure 6e). The fact that the system instead selects a disordered structure with a monodentate linker coordination suggests that the fully ordered variant might not be a stable product due to geometrical frustration and that structure disorder might be essential to allow for the formation of this MOF as in the case of c-(4,12)MTBC-Zr_6_. Alternatively, the fully ordered structure may correspond to the thermodynamic minimum in the shallow energy landscape of all tr-(4,12)MTBC-Zr_6_ variants, but its formation may be kinetically or entropically challenging.

Aiming to obtain a pure tr-(4,12)MTBC-Zr_6_ product, we tested several conditions including variation of the reaction time, temperature, concentration of reactants, solvent, and water content during synthesis from ZrCl_4_ (Supporting Information Sections S1.4 and S1.5). Changes in temperature and concentrations of reactants marginally affect the overall phase composition as analyzed by PXRD and mostly yield the previously described c-(4,12)MTBC-Zr_6_. Increasing the reaction time shifts the yield more in favor of tetragonal PCN-521 and trigonal tr-(4,12)MTBC-Zr_6_, and a smaller percentage of the cubic phase is obtained. When employing dimethylacetamide (DMA) as the solvent instead of DEF to disfavor solvent decomposition, cubic c-(4,12)MTBC-Zr_6_ was not formed. Here, 24 h reaction times yielded a mixture of the trigonal and tetragonal phase, whereas reaction for 3 days considerably shifted the yield in favor of the tetragonal MOF, suggesting that the trigonal phase might transform into the tetragonal phase under these conditions. Key for shifting phase selectivity has been conducting the synthesis with a controlled amount of water. We indeed screened synthetic conditions by employing anhydrous ZrCl_4_ that has been opened and stored under dry conditions in a glovebox and tested the addition of 0, 4, 8, and 12 μL of H_2_O while using identical amounts of reagents dissolved in 3.2 mL of DMA (Supporting Information Table S1.5.1). Reaction under the addition of 4 μL of water yielded hexagonal particles, while synthesis with 8 or 12 μL of water yielded mixtures of hexagonal and octahedral particles, characteristic for the trigonal and tetragonal phases, respectively. Also, in these cases, reaction has a strong effect on phase composition, as phase homogeneity (based on crystal morphology) was only observed for reactions involving 4 μL of water lasting 1 and 2 days, whereas extending reaction times to 3 days results in the appearance of octahedral crystals, which become predominant after 4 days and the only product after 6 and 12 days (Supporting Information Figure S3.2.2). Unfortunately, poor crystallinity and the problematic presence of amorphous content hampered unambiguous phase identification by PXRD (Supporting Information Figures S4.3.1 and S4.3.2). Therefore, the observation of hexagonal platelets should not be linked to the formation of tr-(4,12)MTBC-Zr_6_ with absolute certainty as the presence of unknown MTBC–Zr phases with hexagonal platelet morphology cannot be excluded. The decisive role of water in defining the outcome of the MTBC–Zr system draws interesting parallels to the chemistry of Zr-porphyrinic MOFs, which we recently showed as being highly dependent on water content.^40^ This suggests that water is not only an essential reagent but also a key player in the formation kinetics for the broader Zr MOF family.

### Extension to Hf-MTBC-Based Frameworks

**Figure 7:**
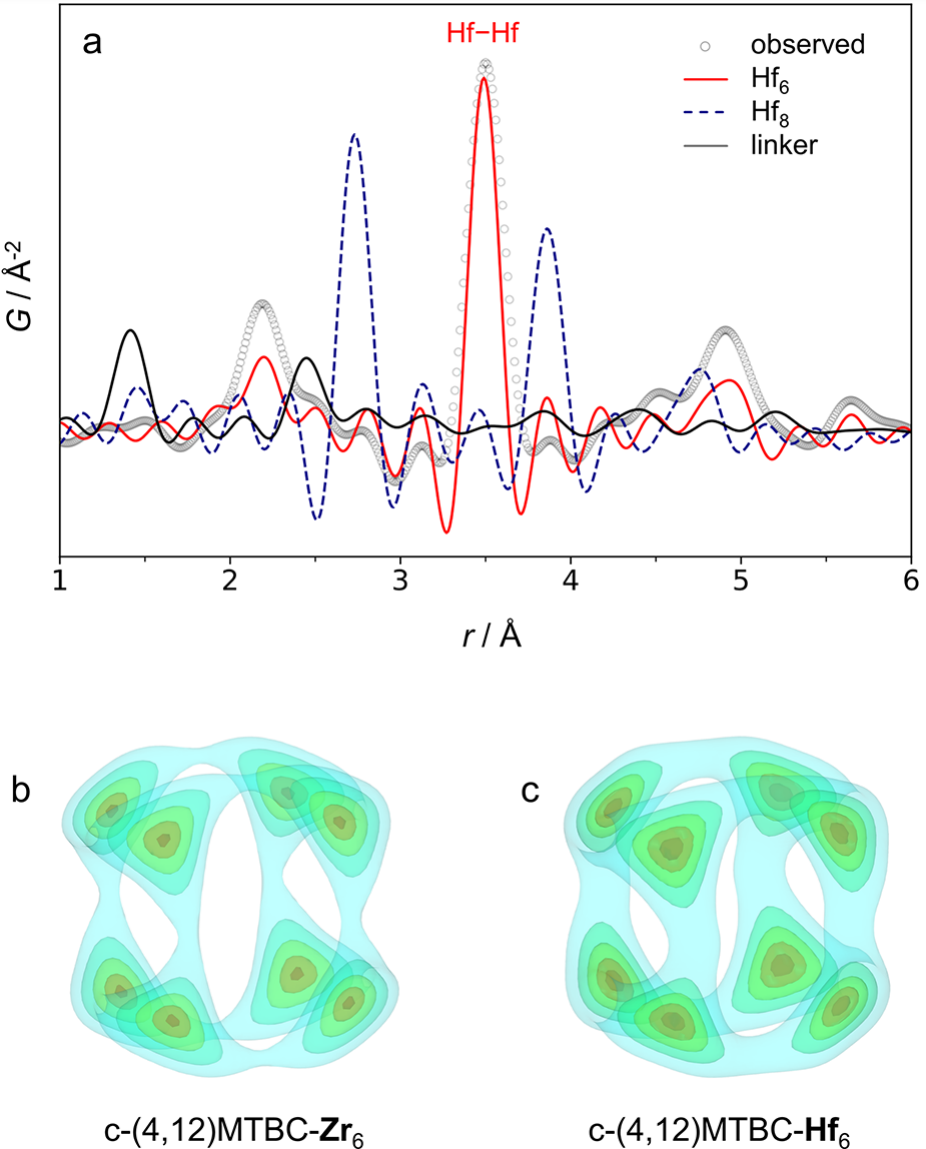
Experimental PDF of c-(4,12)MTBC-Hf_6_ (obtained from synchrotron radiation source with a *Q*_max_ = 21.0 Å^–1^) compared to the simulated PDFs of Hf_6_ and Hf_8_ clusters, and the MTBC linker from the published Hf-MTBC structure (a).^25^ Electron density maps of the disordered cluster sites obtained from SCXRD analysis of c-(4,12)MTBC-Zr_6_ (b) and c-(4,12)MTBC-Hf_6_ (c), both with a **TO** morphology.

The multiphase behavior in Zr-MTBC chemistry is not specific to their chemical composition. Indeed, the Hf-based isostructural analogues of the three discussed Zr-MTBC phases could be obtained by simply changing the metal precursor to HfCl_4_ in the synthesis, thereby yielding cubic c-(4,12)MTBC-Hf_6_, trigonal tr-(4,12)MTBC-Hf_6_, and tetragonal PCN-523 (Figure S3.2.1d,e). Similar to c-(4,12)MTBC-Zr_6_, PDF analysis of c-(4,12)MTBC-Hf_6_ revealed the presence of octahedral Hf_6_O_4_(OH)_4_ clusters only, which suggests that reported cubic Hf_8_ clusters^25^ also result from an incorrect modeling of what in fact is the superposition of orientational disordered Hf_6_O_4_(OH)_4_ clusters (Figure 7a). In fact, the electron density map of the Hf cluster sites obtained from SCXRD analysis resembles the electron density map of the Zr cluster sites in **TO** c-(4,12)MTBC-Zr_6_ crystals (Figure 7b,c). Crystals of c-(4,12)MTBC-Hf_6_ obtained as **TO** are thus isostructural to their Zr-based analogues both in their ordered building blocks and in the four-fold orientational disorder affecting the metal clusters. The multiphase behavior encountered in both Zr- and Hf-MTBC likely results from relatively small changes in energy for the molecular and coordination geometries of the building blocks rather than specifically from their chemical composition. By expanding this concept, analogous structures could be formed by adopting not only metal–oxo clusters with comparable coordination chemistry but also linkers with different lengths but similar binding capability and geometries, i.e., following an isoreticular approach.^41^ Although this might be true in some cases, the importance of the linker’s flexibility should not be underestimated as it might be decisive for whether a given linker–metal combination results in the formation of a crystalline framework as well as its tolerance to locally distorted bonding geometries and/or vacancies.^42^ The structure of tr-(4,12)MTBC-Zr_6_ exemplifies this concept. Here, the geometry of the large MTBC linker strongly deviates from an ideal tetrahedron as the mean angle between the central sp^3^ carbon (linker node) and the terminal sp^2^ carbons ranges approximately from 88 to 123°. Such rather extreme deviations from the ideal 109.5°, necessary for the framework’s architecture, result from a combination of distorted bonding geometry of the sp^3^ carbon and bending of the aromatic arms of the linker. This flexibility naturally increases when using larger linkers in synthesizing isoreticular frameworks, thus suggesting that expanding the linkers’ size while maintaining their geometry could lead to the occurrence of more diverse phase mixtures and possibly unknown polymorphic behaviors.

## Conclusions

The family of Zr-based MOFs presents extremely diverse structural possibilities, particularly with respect to synthesizing different frameworks sharing the same molecular building blocks. In this regard, the work herein presents the multiphase behavior of Zr- and Hf-MTBC, where identical synthesis conditions yield a phase mixture comprising up to three different phases, including the two previously unknown structures c-(4,12)MTBC-Zr_6_/Hf_6_ and tr-(4,12)MTBC-Zr_6_/Hf_6_. Interestingly, in these new frameworks, distinct types of disorder are present, which complicate the rationalization of the MOF synthesis and its optimization toward phase purity. While such disordered states will affect the properties of the material, they can be critical in allowing the formation of such highly connected crystalline structures in the first place. In fact, we showed that these can be achieved through the combination of linker flexibility and cluster disorder, enabling the formation of MOFs with topologies otherwise prohibited by geometrical frustration.

These considerations lead to three main conclusions. First, a broader exploration of synthetic variables such as solvents, methodologies, additives, etc. is needed to achieve selectivity towards the various Zr/Hf-MTBC phases. Second, the new structures we reported highlight how building block disorder can be of crucial benefit in MOF chemistry as a solution to the problem of geometrical frustration and a useful source of new, high-connected framework topologies. In the case of tr-(4,12)MTBC-Zr/Hf_6_, this leads not only to one network but also to virtually infinite variations thereof, depending on how alternative kinds of framework connectivity are distributed in space, which could be, in principle, controlled upon varying synthetic conditions. Lastly, detailed structural information on framework disorder is still relatively rare. The current understanding relies on powder diffraction and spectroscopy data, which are often complex to interpret and yield only spatially averaged information of the material structure. While the importance of acquiring reliable insights into polycrystalline samples is paramount, a more detailed 3D picture of the real structure of MOFs can be extracted from single-crystal diffuse scattering data. This practice is currently rare but highly needed, particularly in the context of reticular chemistry and defect engineering, as it will allow for better understanding of MOF formation when disorder is not induced to create variants of an ordered MOF but is intrinsic to the structure and essential for its formation. Further development and establishment of single-crystal diffraction techniques for both average and local structure analysis, combined with more extended and automated synthetic screenings, will be key to effectively chart the vast compositional and structural landscape of Zr- and Hf-MTBC frameworks as well as many other analogous systems.

## Supporting Information Available

All crystal structures described in our work can be obtained free of charge from the Cambridge Crystallographic Database at the web-page https://www.ccdc.cam.ac.uk/structures/. Their reference numbers are 2182237 [c-(4,12)MTBC-Zr_6_ with cubic morphology], 2215758 [c-(4,12)MTBC-Zr_6_ with **TO** morphology], 2206543 [c-(4,12)MTBC-Hf_6_ with **TO** morphology], 2206443 (tr-(4,12)MTBC-Zr_6_), and 2206530 (tr-(4,12)MTBC-Hf_6_). Observed 3D electron density maps used for the modeling of the reported crystal structures are provided as “.vesta” and “.pgrid” files that can be opened via the free software Vesta.^40^ Additional information on their creation and usage can be found in the Supporting Information (Section S6.3) (ZIP)
Synthesis of Zr-MTBC phases; SEM; optical microscopy; PXRD; XPDF analysis; SCXRD; single-crystal diffuse scattering analysis; topological analysis of tr-(4–12)MTBC-M_6_ and c-(4–12)MTBC-M_6_; remarks on the multiphase behavior in Zr-MTBC MOFs; and nitrogen sorption analysis (PDF)
