# Supplementary material for: Unlocking New Topologies in Zr-Based Metal–Organic Frameworks by Combining Linker Flexibility and Building Block Disorder
Source: J Am Chem Soc. 2023 Apr 26;145(18):10051–60. doi: 10.1021/jacs.2c13731 (PMC10176567; doi:10.1021/jacs.2c13731)
Supplement: Supplementary file 2 — ja2c13731_si_002.pdf [file ja2c13731_si_002.pdf]

Electronic Supplementary Information

Unlocking new Topologies in Zr-based Metal–Organic Frameworks by Combining Linker Flexibility and Building Block Disorder

Charlotte Koschnick,[1,2] Maxwell W. Terban,[1] Ruggero Frison,[3] Martin Etter,[4] Felix A. Böhm,[2] Davide M. Proserpio,[5] Simon Krause,[1] Robert E. Dinnebier,[1] Stefano Canossa,[1,*] Bettina V. Lotsch[1,2,*]

[1] Max Planck Institute for Solid State Research, Heisenbergstraße 1, Stuttgart, 70569, Germany

[2] Department of Chemistry, University of Munich, Butenandtstraße 5-13, Münich, 81377, Germany

[3] Physik-Institut, Universität Zürich, Winterthurerstrasse 190, CH-8057 Zürich, Switzerland

[4] Deutsches Elektronen-Synchrotron (DESY), Notkestraße 85, Hamburg, 22607, Germany

[5] Universita' degli Studi di Milano, Dipartimento di Chimica, Via Golgi 19, 20133 Milano, Italy

* s.canossa@fkf.mpg.de; b.lotsch@fkf.mpg.de

Table of Contents

S1. Synthesis of Zr-MTBC phases
- S1.1. Materials and general information 2
- S1.2. Synthesis of cubic Zr-MTBC according to literature 2
- S1.3. Adapted synthesis of cubic and trigonal (4,12)MTBC-Zr_6 and (4,12)MTBC-Hf_6 2
- S1.4. Screening of reaction conditions in DEF 2
- S1.5. Screening of reaction conditions in DMA 3

S2. Scanning electron microscopy (SEM)
- S2.1. Instrumental details 4
- S2.2. SEM micrographs 4

S3. Optical microscopy
- S3.1. Instrumental details 6
- S3.2. Optical microscopy images 6

S4. Powder X-ray diffraction (PXRD)
- S4.1. Data collection and processing details 8
- S4.2. Rietveld refinement plots 8
- S4.3. Additional PXRD measurements 15

S5. X-ray pair distribution function (XPDF) analysis
- S5.1. Data collection and processing details 16
- S5.2. XPDF analysis of truncated-octahedral c-(4-12)MTBC-Zr_6 17

S6. Single crystal X-ray diffraction (SCXRD)
- S6.1. Data collection, processing and general modelling details 18
- S6.2. SCXRD refinement details 19
- S6.3. 3D electron density maps availability 29

S7. Single crystal diffuse scattering analysis
- S7.1. 3D reciprocal space reconstructions and processing 33
- S7.2. Diffuse scattering simulations 40

S8. Topological analysis of tr-(4-12)MTBC-M_6 and c-(4-12)MTBC-M_6 41

S9. Remarks on the multiphase behaviour in Zr-MTBC MOFs 45

S10. Nitrogen sorption analysis 46

References 47

S1. Synthesis of Zr-MTBC phases

S1.1. Materials and general information

4',4'',4''',4''''-methanetetrayltetrabiphenyl-4-carboxylic acid (MTBC) was purchased from Alfa Chemistry. Zirconium(IV) chloride ($ZrCl_4$), Hafnium(IV) chloride ($HfCl_4$), benzoic acid, acetone, and N,N-dimethylacetamide (DMA) were purchased from Sigma Aldrich. N,N-diethylformamide (DEF) was purchased from Alfa Aesar. All chemicals were used as received without further purification. Note that the employed $ZrCl_4$ was either stored in the glovebox under inert gas ($ZrCl_4$_GB) or at ambient air ($ZrCl_4$_air) where it was prone for hydrolysis.[1]

Ultrasonication was conducted via an ELMASONIC S 100 bath equipped with a high-performance 37 kHz sandwich transducer and state-of-the-art microprocessor. Centrifugation was performed with a benchtop centrifuge Sigma-3-30K from SIGMA. For SEM analysis MOF suspensions were spin-coated onto silicon wafers with a WS-650S-NPP Lite device from Laurell Technology Corporation.

S1.2. Synthesis of cubic Zr-MTBC according to literature

Zr-MTBC was synthesised according to a procedure published by Ji *et al.*[2] $ZrCl_4$ (18.2 mg, 0.0781 mmol), MTBC (16 mg, 0.020 mmol), and benzoic acid (554 mg, 4.54 mmol)) were ultrasonically dissolved in DEF (3.2 mL) in a 5 mL microwave vial. The mixture was heated in an Al-block (24 h, pre-heated to 120 °C) under stirring. After cooling to room temperature outside the Al-block, the product was collected by centrifugation and washed with DEF (three times, 16k rpm/15 min/16 °C) and acetone (twice, 16k rpm/15 min/16 °C). The mixture was soaked in acetone over night and washed with acetone once. Supercritical CO_2 drying yielded a white powder as product.

S1.3. Adapted synthesis of cubic and trigonal (4,12)MTBC-Zr_6 and (4,12)MTBC-Zr_6

$ZrCl_4$ (18.2 mg, 0.0781 mmol), MTBC (16.0 mg, 0.020 mmol), and benzoic acid (554 mg, 4.54 mmol)) were ultrasonically dissolved in DEF (3.2 mL) in a 20 mL pyrex vial. The mixture was heated in an oven pre-heated to 120 °C for 24 h. After cooling to room temperature outside the Al-block, the formed crystals were washed with DEF twice and acetone three times (soaked in acetone over night during last washing step). The crystals were dried using supercritical CO_2. Single crystals of cubic and trigonal (4,12)-MTBC-Hf_6 were obtained analogously by reacting $HfCl_4$ (25.0 mg, 0.781 mmol) instead of $ZrCl_4$ for 48 h or 7 d in an oven.

S1.4. Screening of reaction conditions in DEF

$ZrCl_4$, MTBC, and benzoic acid were ultrasonically dissolved in DEF (3.2 mL) in a 20 mL pyrex vial. The solution was heated in an oven at 100 or 120 °C for 24 h to 7d. In the case of MOF_supernatant, the supernatant obtained after 24 h reaction was further reacted at 120 °C for 72 h. For all reactions, the formed crystals were washed with DEF or DMA twice and acetone three times (soaked in acetone over night during last washing step). The crystals were dried using supercritical CO_2.

Table S1.4.1. Amount of $ZrCl_4$, MTBC, and benzoic acid (BzA), reaction time, and reaction temperature (Temp.) employed during the screening of reaction conditions in DEF.

Sample	$ZrCl_4$	MTBC	BzA	Time	Temp.
MOF_100	*$ZrCl_4$_air* 18.2 mg, 0.0781 mmol	16.0 mg, 0.020 mmol	554 mg, 4.54 mmol	48 h	100 °C
MOF_120	*$ZrCl_4$_air* 18.2 mg, 0.0781 mmol	16.0 mg, 0.020 mmol	554 mg, 4.54 mmol	24 h	120 °C
MOF_less_MTBC	*$ZrCl_4$_air* 18.2 mg, 0.0781 mmol	10.4.0 mg 0.013 mmol	554 mg, 4.54 mmol	24 h	120 °C
MOF_ less_$ZrCl_4$+MTBC	*$ZrCl_4$_air* 9.0 mg 0.039 mmol	8.0 mg 0.010 mmol	554 mg, 4.54 mmol	5 d	120 °C

MOF_7d	*$ZrCl_4$_air* 18.2 mg, 0.0781 mmol	16.0 mg, 0.020 mmol	554 mg, 4.54 mmol	7 d	120 °C
MOF_supernatant	*$ZrCl_4$_air* 18.2 mg, 0.0781 mmol	16.0 mg, 0.020 mmol	554 mg, 4.54 mmol	24 h (1) 72 h (2)	120 °C

S1.5. Screening of reaction conditions in DMA

$ZrCl_4$ (stored at ambient air or new from glovebox), MTBC, benzoic acid, and water (see Table S.1.4.2. for more details) were ultrasonically dissolved in DMA (3.2 mL) in a 20 mL pyrex vial. The solution was heated in an oven (24 h to 12d, pre-heated to 120 °C). For all reactions, the formed crystals were washed with DEF twice and acetone three times (soaked in acetone over night during last washing step). The crystals were dried using supercritical CO_2.

Table S1.5.1. Amount of $ZrCl_4$ (ambient air = air; glove box = GB), MTBC, and benzoic acid (BzA), as well as reaction time, and reaction temperature (temp.) employed during the screening of reaction conditions in DMA. The results evidence the crucial role of water content on the reactions' outcome, as we also showed in previous study.[1]

Sample	$ZrCl_4$	MTBC	BzA	Water	Time	Temp.	Yield
MOF_DMA_18h	*$ZrCl_4$_air* 18.2 mg, 0.0781 mmol	16.0 mg, 0.020 mmol	554 mg, 4.54 mmol	0 µl	18 h	120 °C	No product
MOF_DMA_1d	*$ZrCl_4$_air* 18.2 mg, 0.0781 mmol	16.0 mg, 0.020 mmol	554 mg, 4.54 mmol	0 µl	1 d	120 °C	N/A
MOF_DMA_2d	*$ZrCl_4$_air* 18.2 mg, 0.0781 mmol	16.0 mg, 0.020 mmol	554 mg, 4.54 mmol	0 µl	2 d	120 °C	N/A
MOF_DMA_1d_0	*$ZrCl_4$_GB* 18.2 mg, 0.0781 mmol	16.0 mg, 0.020 mmol	554 mg, 4.54 mmol	0 µl	1 d	120 °C	No product
MOF_DMA_1d_4	*$ZrCl_4$_GB* 18.2 mg, 0.0781 mmol	16.0 mg, 0.020 mmol	554 mg, 4.54 mmol	4 µl	1 d	120 °C	<0.1 mg
MOF_DMA_2d_4	*$ZrCl_4$_GB* 18.2 mg, 0.0781 mmol	16.0 mg, 0.020 mmol	554 mg, 4.54 mmol	4 µl	2 d	120 °C	0.5 mg
MOF_DMA_3d_4	*$ZrCl_4$_GB* 18.2 mg, 0.0781 mmol	16.0 mg, 0.020 mmol	554 mg, 4.54 mmol	4 µl	3 d	120 °C	20 mg
MOF_DMA_4d_4	*$ZrCl_4$_GB* 18.2 mg, 0.0781 mmol	16.0 mg, 0.020 mmol	554 mg, 4.54 mmol	4 µl	4 d	120 °C	22 mg
MOF_DMA_5d_4	*$ZrCl_4$_GB* 18.2 mg, 0.0781 mmol	16.0 mg, 0.020 mmol	554 mg, 4.54 mmol	4 µl	5 d	120 °C	20 mg
MOF_DMA_6d_4	*$ZrCl_4$_GB* 18.2 mg, 0.0781 mmol	16.0 mg, 0.020 mmol	554 mg, 4.54 mmol	4 µl	6 d	120 °C	25 mg
MOF_DMA_12d_4	*$ZrCl_4$_GB* 18.2 mg, 0.0781 mmol	16.0 mg, 0.020 mmol	554 mg, 4.54 mmol	4 µl	12 d	120 °C	25 mg

MOF_DMA_1d_8	*ZrCl$_4$_GB* 18.2 mg, 0.0781 mmol	16.0 mg, 0.020 mmol	554 mg, 4.54 mmol	8 µl	1 d	120 °C	5 mg
MOF_DMA_2d_8	*ZrCl$_4$_GB* 18.2 mg, 0.0781 mmol	16.0 mg, 0.020 mmol	554 mg, 4.54 mmol	8 µl	2 d	120 °C	N/A
MOF_DMA_1d_12	*ZrCl$_4$_GB* 18.2 mg, 0.0781 mmol	16.0 mg, 0.020 mmol	554 mg, 4.54 mmol	12 µl	1 d	120 °C	N/A
MOF_DMA_2d_12	*ZrCl$_4$_GB* 18.2 mg, 0.0781 mmol	16.0 mg, 0.020 mmol	554 mg, 4.54 mmol	12 µl	1 d	120 °C	N/A

S2. Scanning Electron Microscopy

S2.1. Instrumental details

Scanning electron microscopy images were acquired by a Merlin Zeiss Scanning Electron Microscope. For the image acquisition, a secondary electron (SE) detector was used in combination with an accelerating voltage of 1.5 kV. No coating was applied on the samples. Particle sizes were measured with the software ImageJ.[3]

S2.2. SEM micrographs

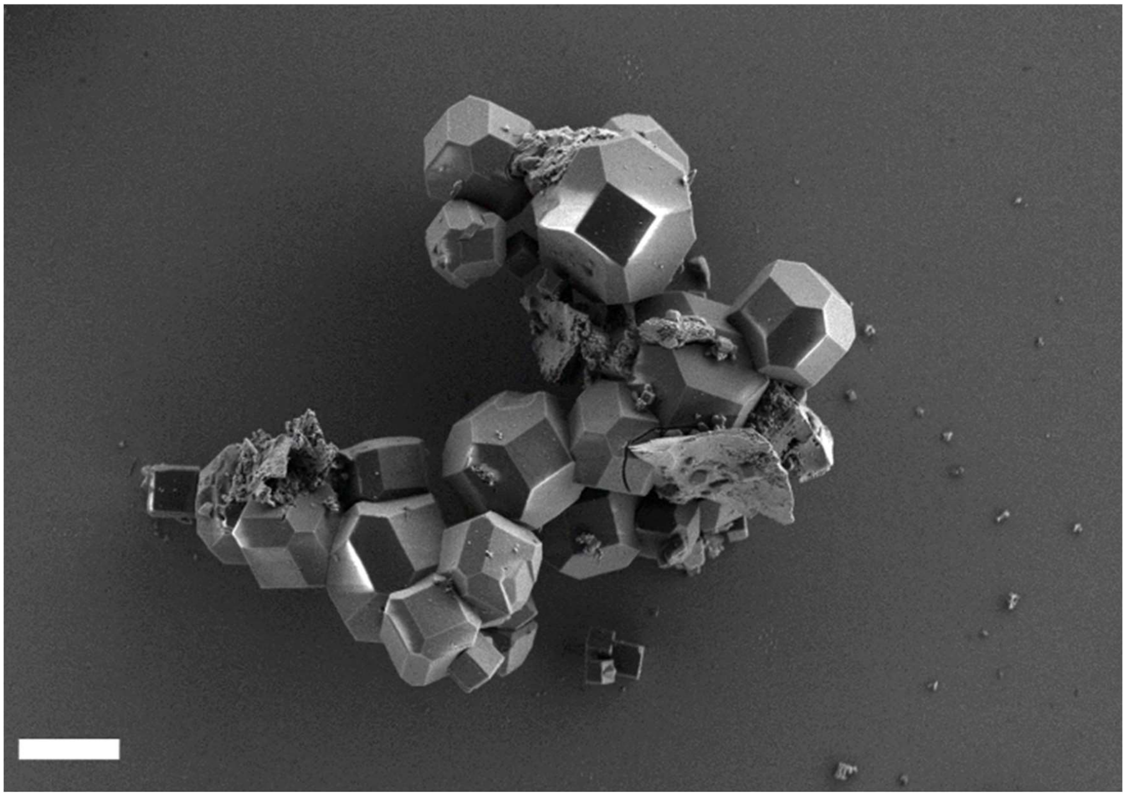

Figure S2.2.1. Scanning electron microscopy image of Zr-MTBC particles synthesized according to literature. Scale bar = 5 µm.

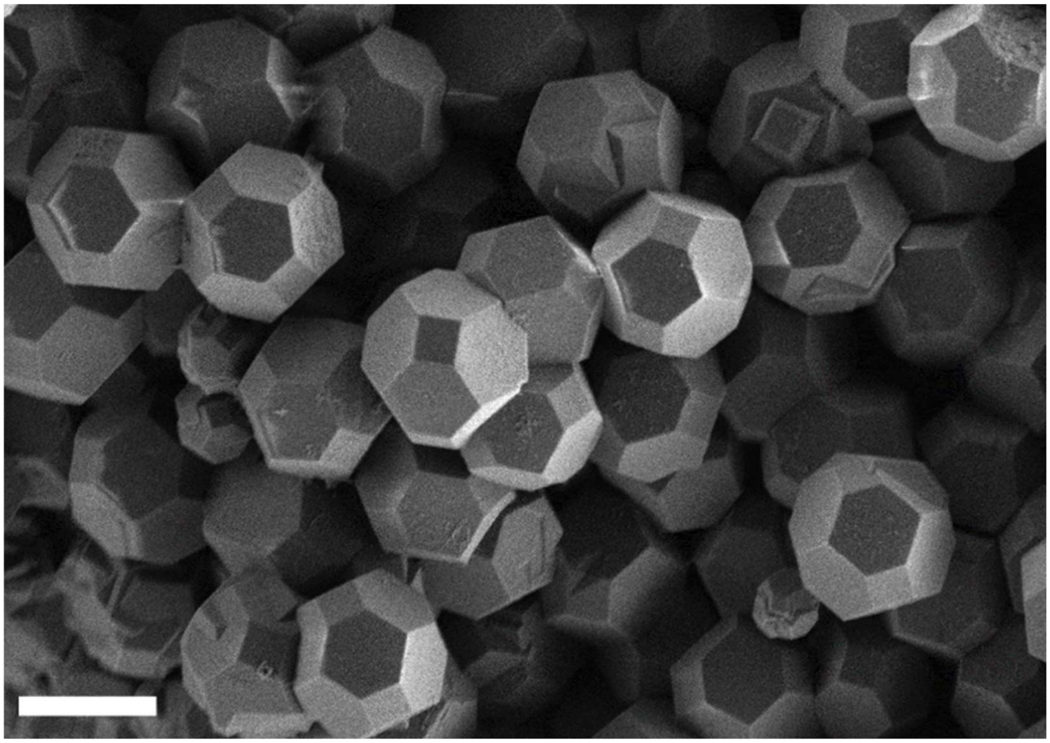

Figure S2.2.2. Scanning electron microscopy image of c-(4,12)MTBC-Zr_6 particles synthesized at 100 °C (MOF_100). Scale bar = 5 μm.

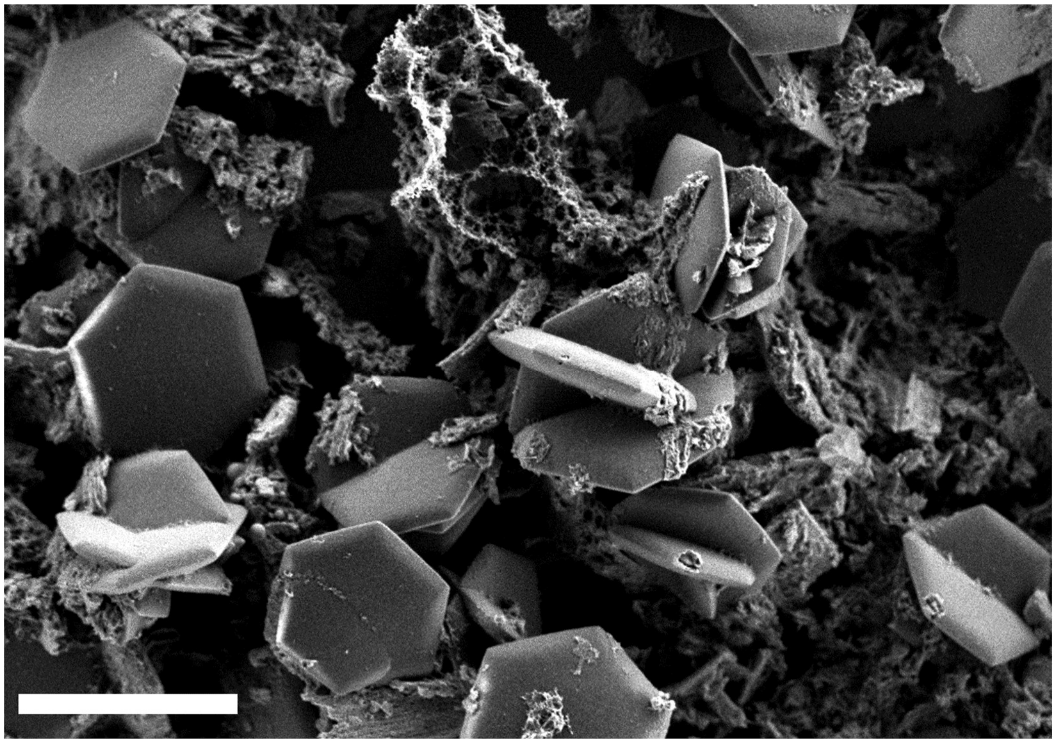

Figure S2.2.3. Scanning electron microscopy image of MOF_DMA_1d_4 obtained after 24 h synthesis at 120 °C in DMA. Scale bar = 5 μm.

S3. Optical microscopy

S3.1. Instrumental details

Optical microscopy images were acquired with a CMOS camera connected to a LEICA DM2500 light microscope, after depositing the crystals on a glass slide, and using the software Stream Essentials v. 1.7.

S3.2. Optical microscopy images

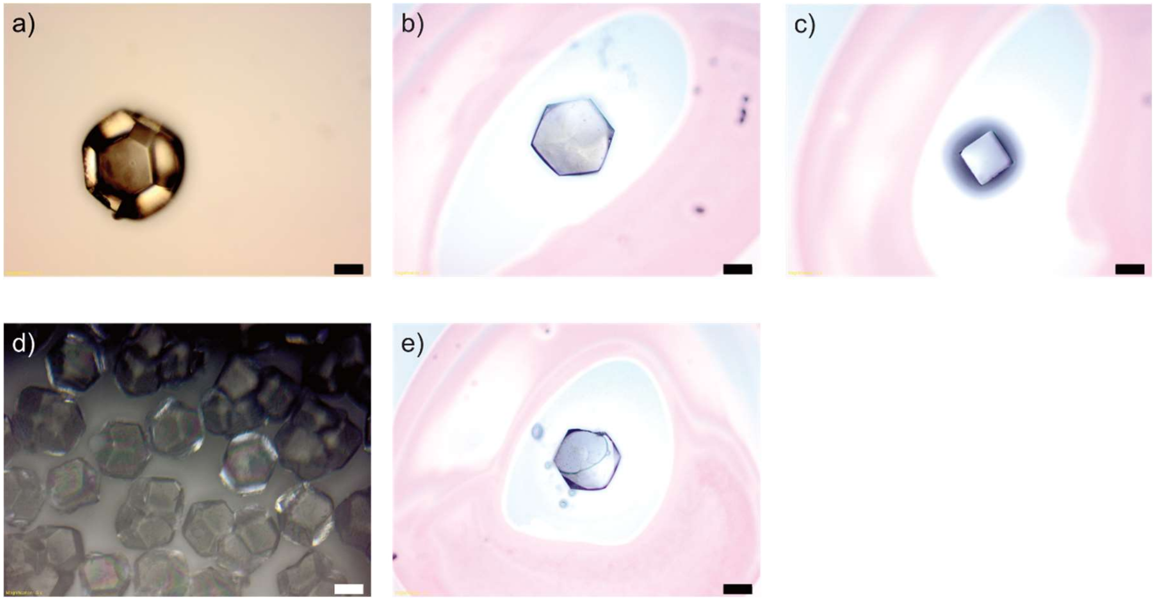

Figure S3.2.1 Microscope images of truncated-octahedral c-(4,12)MTBC-Zr_6 (a), trigonal tr-(4,12)MTBC-Zr_6 (b), cube-shaped c-(4,12)MTBC-Zr_6 (c), truncated-octahedral c-(4,12)MTBC-Hf_6 (d) , and trigonal tr-(4,12)MTBC-Hf_6 singles crystals (e). Scale bar corresponds to 100 µm.

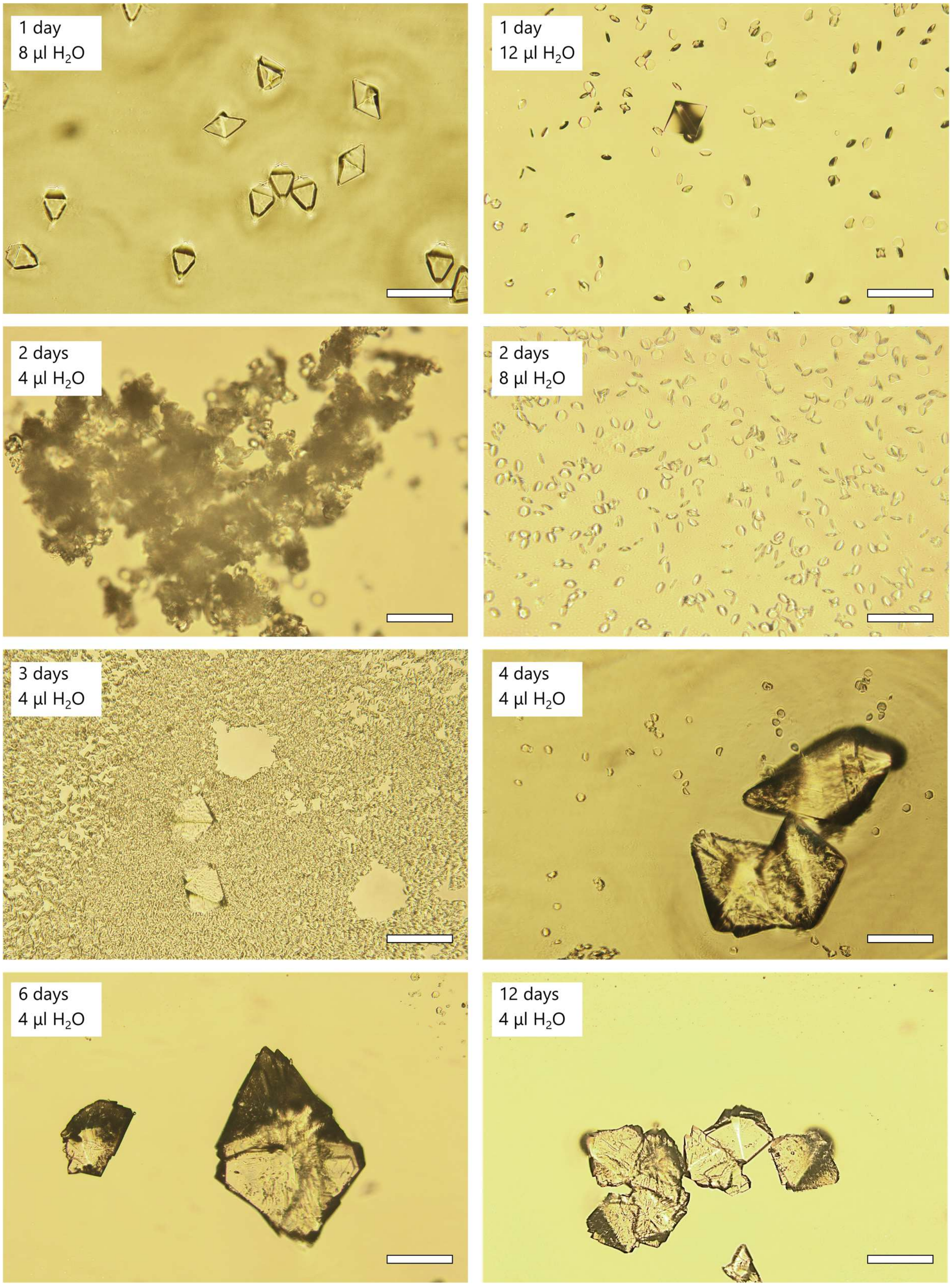

Figure S3.2.2. Optical microscopy images of the products obtained from neat $ZrCl_4$ under addition of various amount of water at different reaction times. All scale bars correspond to 100 µm.

S4. X-ray powder diffraction (XRPD)

S4.1. Data collection and processing details

XRPD patterns were collected at room temperature on a Stoe Stadi-P diffractometer with Cu-Kα_1 radiation (λ = 1.540596 Å) or Co-Kα_1 radiation (λ = 1.78896 Å), a Ge(111) Johann monochromator, and a DECTRIS Mythen 1K detector in Debye-Scherrer geometry. The samples were loaded into 0.5/0.7 mm inner diameter polyimide capillaries and measured over a range of 2θ = 2.000–30.695˚, with 0.015˚ step size and 50 s counting time per step when using Cu-Kα_1 radiation and a range of 2θ = 0.500–115.325˚, with 0.015˚ step size and 200 s counting time per step when using Co-Kα_1 radiation. Rietveld refinements were performed with TOPAS v6.[4] Diffraction data collected using Co-Kα_1 radiation were used for pure phase cubic refinements. A 2θ offset correction, simple axial model, Lorentzian and Gaussian crystallite size broadening convolutions, and either Gaussian/Lorentzian convolution or Stephens model for strain were used to correct for instrumental and morphological peak-shape effects. The background was described using Chebychev polynomials of 11th order and One_on_X term for increased low angle background. Scale factors, lattice parameters, and isotropic atomic displacement parameters were refined over a range of 2.5–80° 2θ. Refinements of mixed phase models used data collected from Cu-Kα_1 radiation. For these refinements, the background was described with 4th/5th order polynomials and One_on_X term. Only scale factors and lattice parameters were refined for the respective phases. A second set of refinements was performed using spherical harmonics corrections of 4th or 6th order for majority phases to account for mismatched relative peak intensities.

S4.2. Rietveld refinement plots

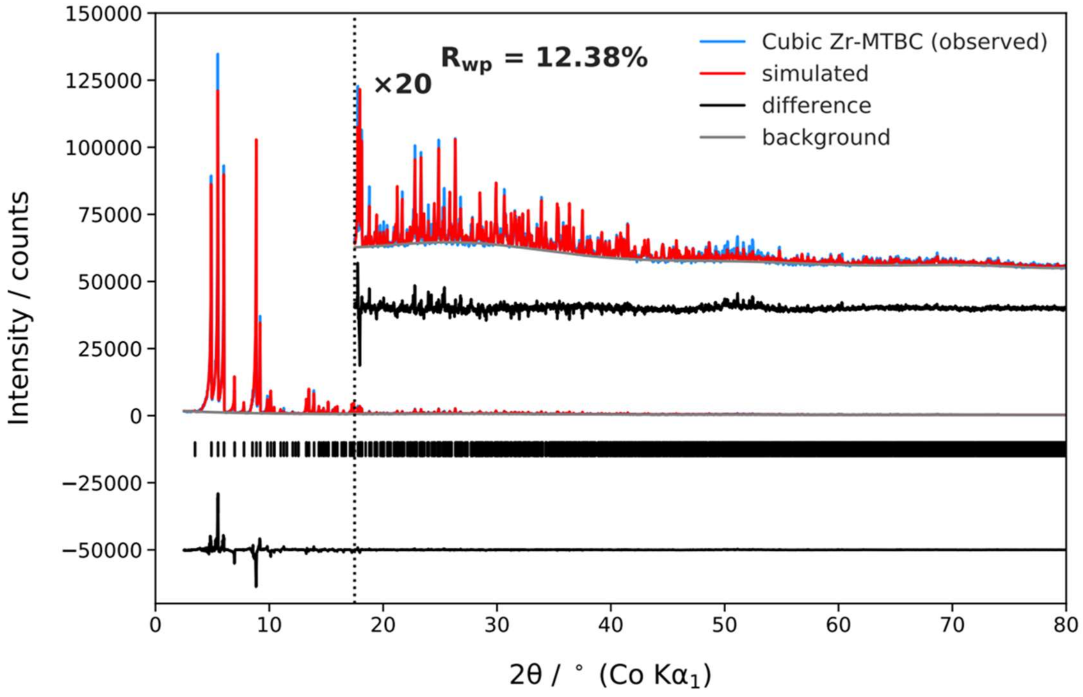

Figure S4.2.1. Rietveld refinement of the new c-(4,12)MTBC-Zr_6 structure model against the observed pattern of experimental truncated-octahedral c-(4,12)MTBC-Zr_6.

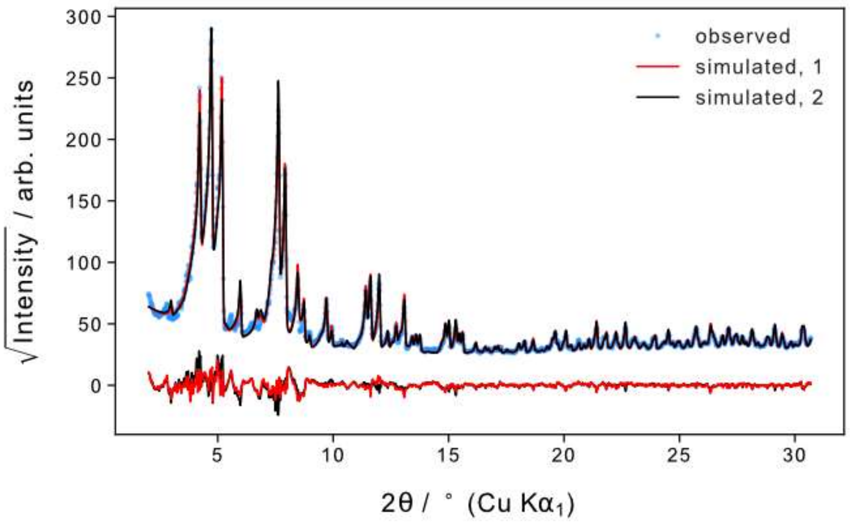

Figure S4.2.2. Rietveld refinement allowing for reflexes of cubic, trigonal, and tetragonal MTBC-Hf_6 against observed PXRD pattern of the product obtained from $HfCl_4$ from synthesis at 120 °C after 48 h reaction time. Simulation 1 includes spherical harmonics corrections of 4-6th order on the predominant phases to account for mismatched relative peak intensities, while simulation 2 does not. Refined composition: 91 % c-(4,12)MTBC-Hf_6 and 9 % tr-(4,12)MTBC-Hf_6.

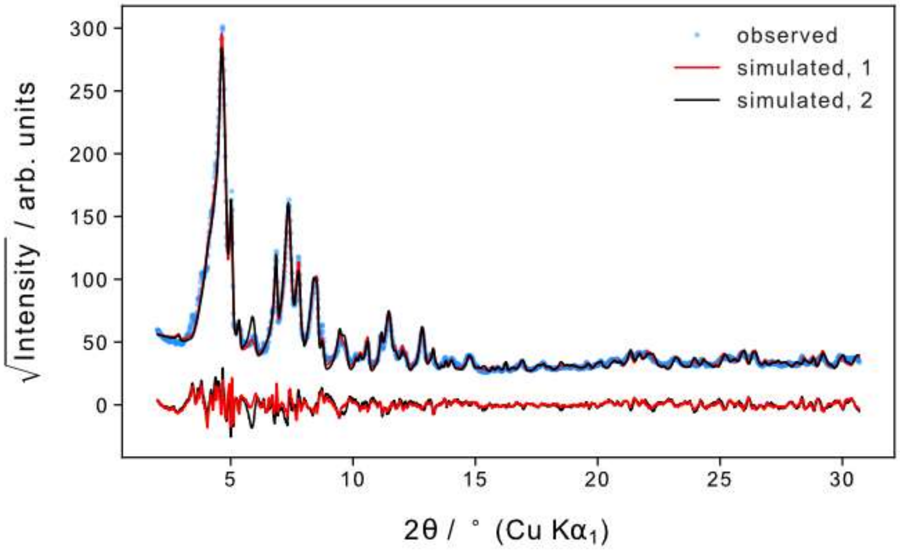

Figure S4.2.3. Rietveld refinement allowing for reflexes of cubic (c), trigonal (t), and tetragonal MTBC-Hf_6 against observed PXRD pattern of the product obtained from $HfCl_4$ from synthesis at 120 °C after 7 d reaction time. Simulation 1 includes spherical harmonics corrections of 4-6th order on the predominant phases to account for mismatched relative peak intensities, while simulation 2 does not. Refined composition: 50 % tetragonal MTBC-Hf_6 (PCN-523), 43 % tr-(4,12)MTBC-Hf_6, and 16 % c-(4,12)MTBC-Hf_6.

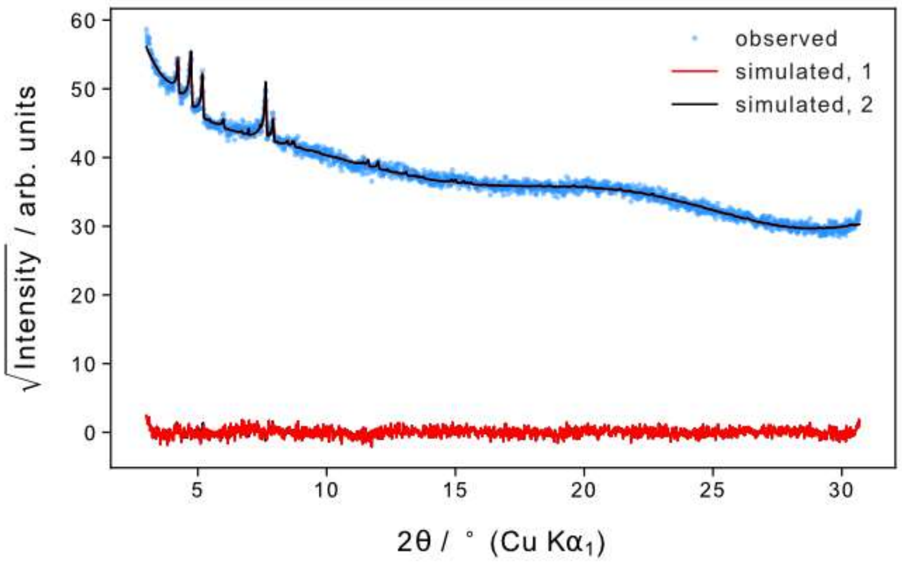

Figure S4.2.4. Rietveld refinement allowing for reflexes of cubic, trigonal, and tetragonal MTBC-Zr_6 against observed PXRD pattern of the product obtained from synthesis at 100 °C. Simulation 1 includes spherical harmonics corrections of 4-6th order on the predominant phases to account for mismatched relative peak intensities, while simulation 2 does not.

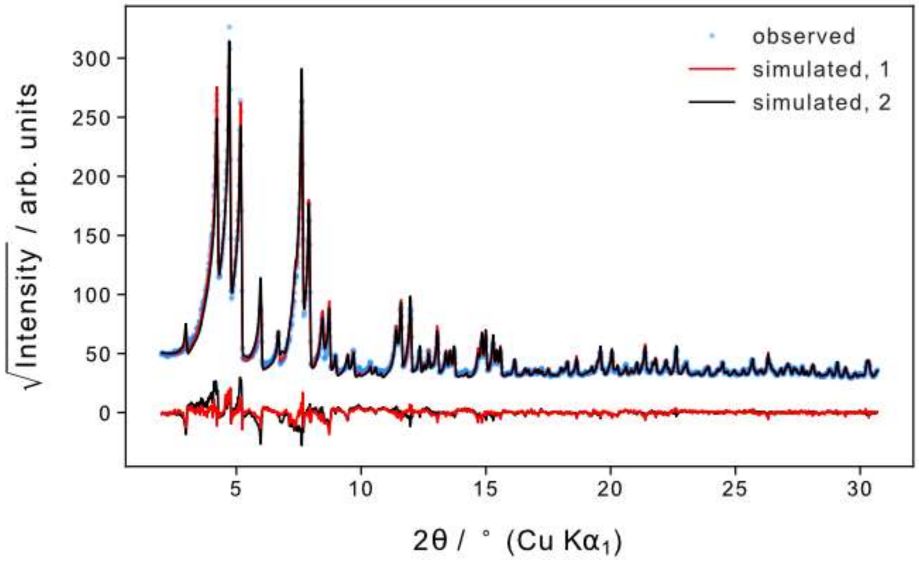

Figure S4.2.5. Rietveld refinement allowing for reflexes of cubic, trigonal, and tetragonal MTBC-Zr_6 against observed PXRD pattern of the product obtained from synthesis at 120 °C. Simulation 1 includes spherical harmonics corrections of 4-6th order on the predominant phases to account for mismatched relative peak intensities, while simulation 2 does not.

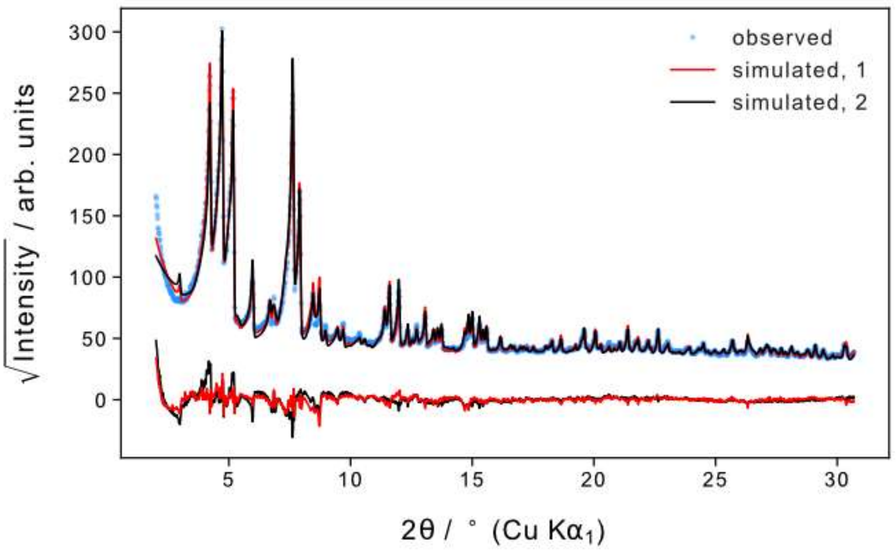

Figure S4.2.6. Rietveld refinement allowing for reflexes of cubic, trigonal, and tetragonal MTBC-Zr_6 against observed PXRD pattern of the product obtained from synthesis with reduced MTBC-concentration (MOF_MTBC_reduced). Simulation 1 includes spherical harmonics corrections of 4-6th order on the predominant phases to account for mismatched relative peak intensities, while simulation 2 does not.

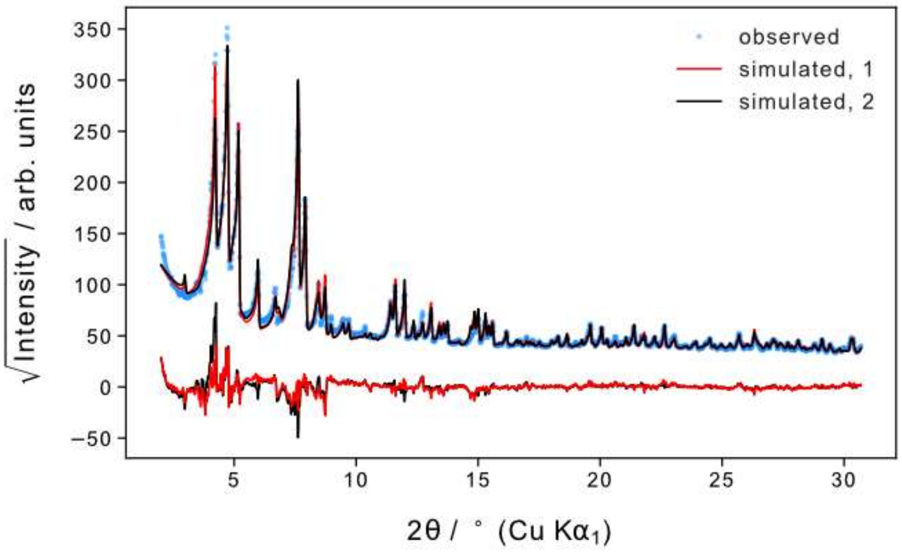

Figure S4.2.7. Rietveld refinement allowing for reflexes of cubic, trigonal, and tetragonal MTBC-Zr_6 against observed PXRD pattern of the product obtained from synthesis with reduced $ZrCl_4$- and MTBC- concentration (MOF_$ZrCl_4$+MTBC_reduced). Simulation 1 includes spherical harmonics corrections of 4-6th order on the predominant phases to account for mismatched relative peak intensities, while simulation 2 does not.

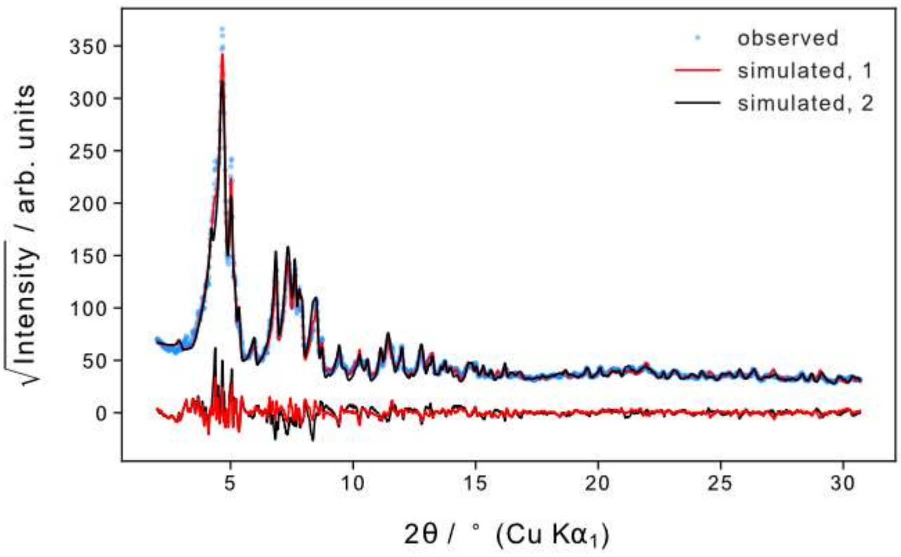

Figure S4.2.8. Rietveld refinement allowing for reflexes of cubic, trigonal, and tetragonal MTBC-Zr_6 against observed PXRD pattern of the product obtained from product after 7d reaction at 120 °C (MOF_7d). Simulation 1 includes spherical harmonics corrections of 4-6th order on the predominant phases to account for mismatched relative peak intensities, while simulation 2 does not.

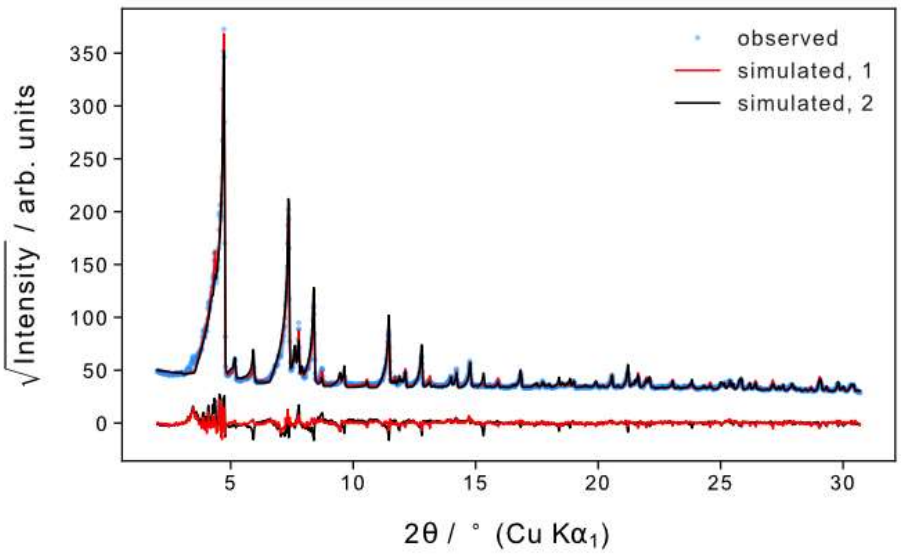

Figure S4.2.9. Rietveld refinement allowing for reflexes of cubic, trigonal, and tetragonal MTBC-Zr_6 against observed PXRD pattern of the product obtained from product after further reaction of the supernatant (MOF_supernatant). Simulation 1 includes spherical harmonics corrections of 4-6th order on the predominant phases to account for mismatched relative peak intensities, while simulation 2 does not.

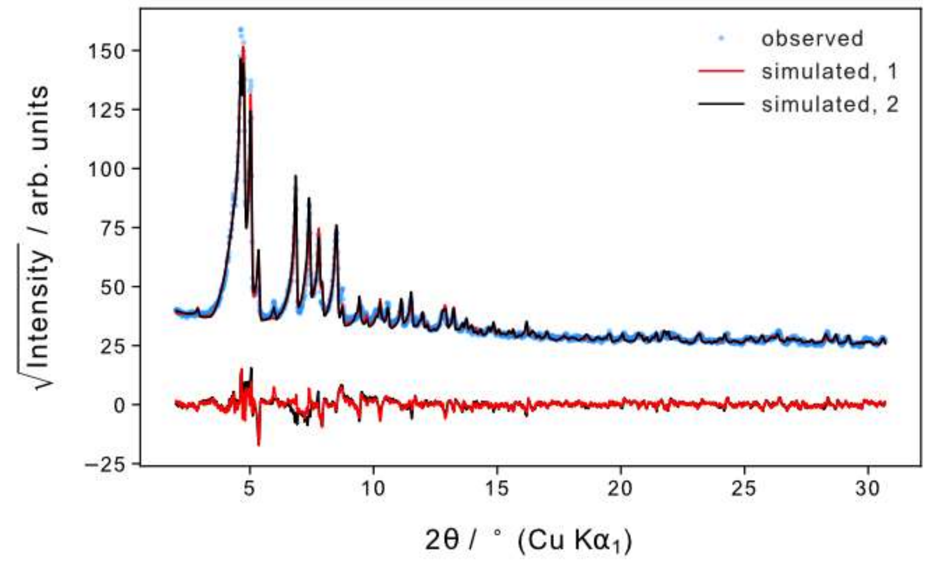

Figure S4.2.10. Rietveld refinement allowing for reflexes of cubic, trigonal, and tetragonal MTBC-Zr_6 against observed PXRD pattern of the product obtained from product from synthesis in DMA for 24 h (MOF_DMA_24h). Simulation 1 includes spherical harmonics corrections of 4-6th order on the predominant phases to account for mismatched relative peak intensities, while simulation 2 does not.

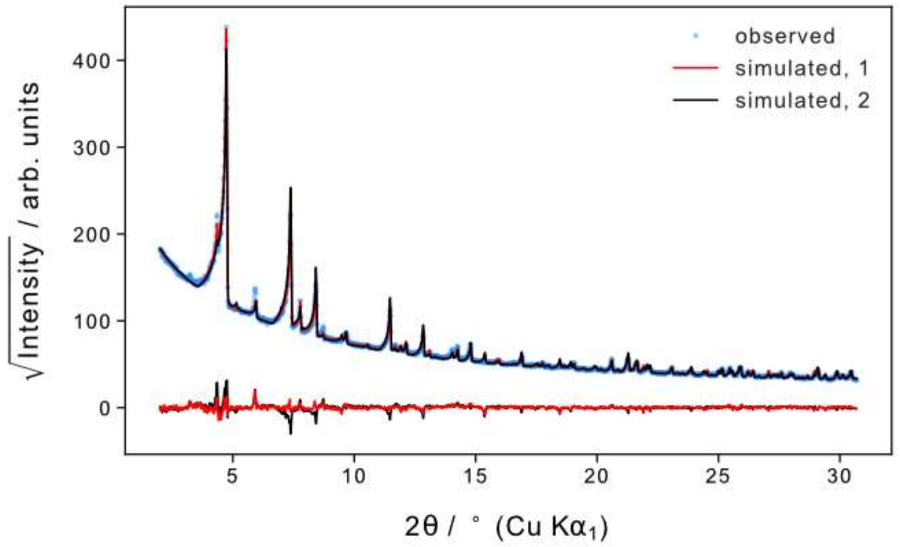

Figure S4.2.11. Rietveld refinement allowing for reflexes of cubic, trigonal, and tetragonal MTBC-Zr_6 against observed PXRD pattern of the product obtained from product from synthesis in DMA for 48 h (MOF_DMA_48h). Simulation 1 includes spherical harmonics corrections of 4-6th order on the predominant phases to account for mismatched relative peak intensities, while simulation 2 does not.

Table S4.2.1. Composition of tetragonal, cubic, and trigonal (4,12)MTBC-Zr_6 obtained from Rietveld refinements from MOF products synthesized under variable reaction conditions.

	100 °C	120 °C	less MTBC	less $ZrCl_4$+MTBC	7d	Super-natant	DMA 24h	DMA 48h
**no spherical harmonics correction**								
**Tetragonal**	2.19	2.2	1.41	4.38	36.18	90.4	31.46	94.73
**Cubic**	90.16	92.91	86.06	86.55	20.09	8.16	3.31	4.03
**Trigonal**	7.65	4.89	12.54	9.07	43.73	1.44	65.24	1.24
**spherical harmonics correction**								
**Tetragonal**	2.44	3.38	2.19	4.44	40.28	93.57	30.78	99.78
**Cubic**	92.33	94.51	90.89	88.64	19.69	6.09	0.95	0
**Trigonal**	5.24	2.11	6.92	6.92	40.03	0.34	68.27	0.22
**average**								
**Tetragonal**	2	3	2	4	38	92	31	97
**Cubic**	91	94	88	88	20	7	2	2
**Trigonal**	6	4	10	8	42	1	67	1
**standard deviation**								
**Tetragonal**	0.4	2	1	0.08	6	4	1	7
**Cubic**	3	2	7	3	1	3	3	6
**Trigonal**	3	4	8	3	5	2	4	1

S4.3. Additional PXRD measurements

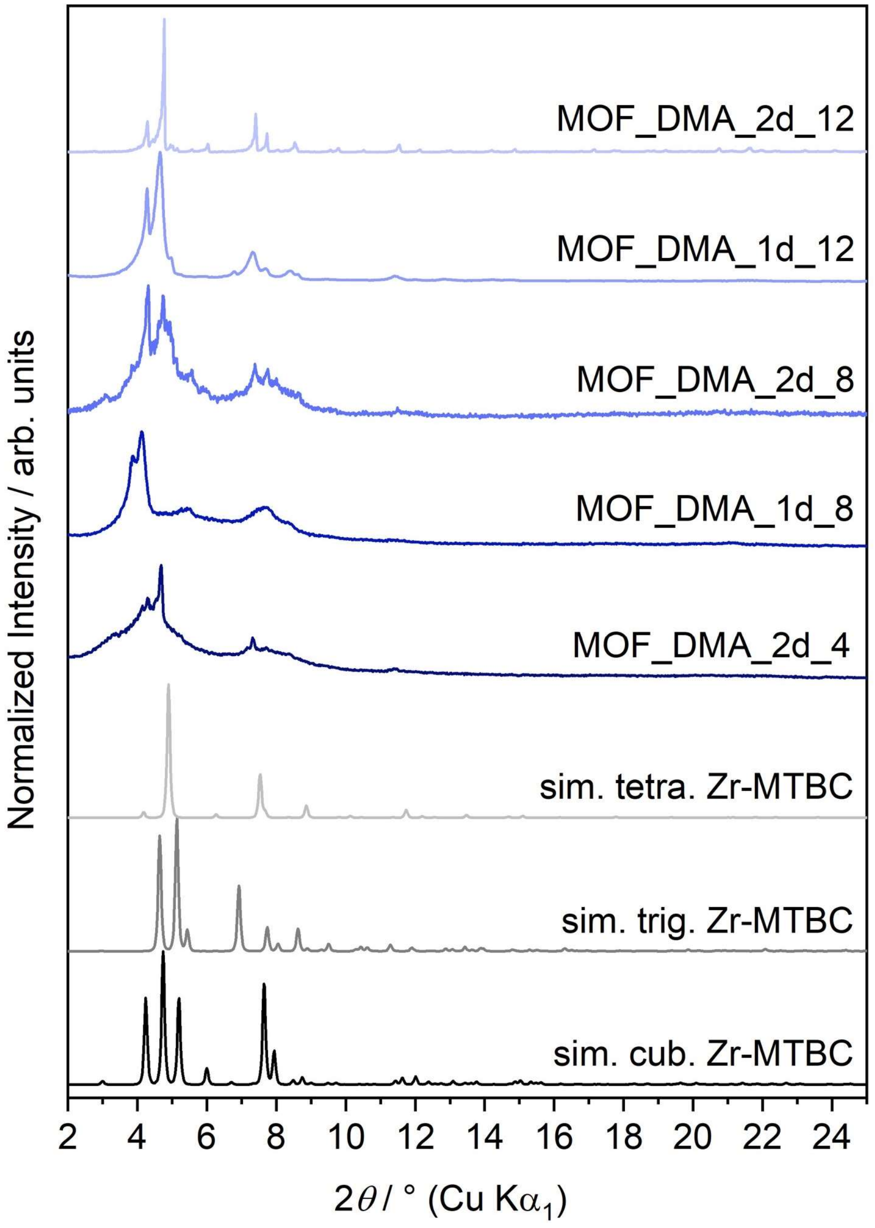

Figure S4.3.1. PXRD pattern of samples obtained from synthesis in DMA with of 4, 8 and 12 µL H_2O compared to simulated patterns (sim.) of cubic Zr-MTBC, trigonal Zr-MTBC, and tetragonal Zr-MTBC (PCN-521). Simulated patterns are computed from structures taken from the single crystal refinements, which used diffraction data at 100K and not at room temperature.

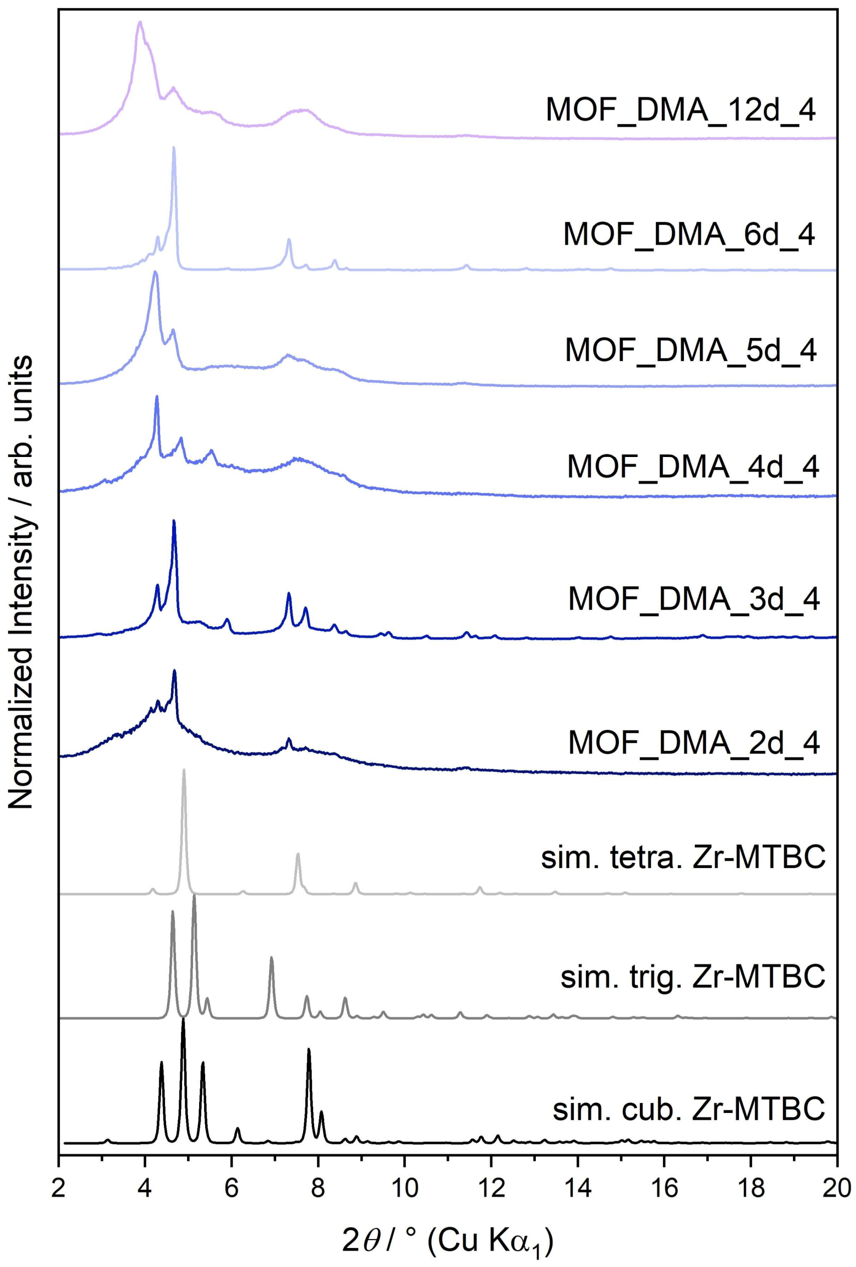

Figure S4.3.2. PXRD pattern of the samples obtained from synthesis in DMA under addition of 4 µL water compared to the simulated patterns (sim.) of cubic Zr-MTBC, trigonal Zr-MTBC, and tetragonal Zr-MTBC (PCN-521). Simulated patterns are computed from structures taken from the single crystal refinements, which used diffraction data at 100K and not at room temperature.

S5. X-ray pair distribution function (XPDF) analysis

S5.1. Data collection and processing details

XPDF analysis was carried out using P02.1, the Powder Diffraction and Total Scattering Beamline, at PETRA III of the Deutsches Elektronen-Synchrotron (DESY). The rapid acquisition PDF method (RAPDF)[5] was used with a large-area 2D PerkinElmer detector (2048×2048 pixels, 200×200 µm^2 each) and sample-to-detector distance of 481.242 mm. The incident energy of the x-rays was 59.795 keV (λ = 0.20735 Å). Samples were loaded into 1 mm inner diameter glass

capillaries. An empty capillary was measured as background and subtracted, and a LaB6 standard was measured at room temperature for calibration of the setup. Calibration, polarization correction, and azimuthal integration to 1D diffraction patterns were performed using the software pyFAI.[6,7]

Additional total scattering measurements were performed on the same samples in-lab using a Stoe Stadi-P diffractometer with Mo Kα_1 radiation (λ = 0.7093 Å), a Ge(111) Johann monochromator, and a DECTRIS Mythen 1K detector in Debye-Scherrer geometry. Measurements were carried out over a range of 2θ = 0.405–110.565˚ and 40.50–110.565˚ with 0.405˚ step size and 150 s count time per step, and 81.00–110.565˚ with 300 s count time. Data ranges were then combined and directly corrected for the 2θ offset of the instrument and polarization. Further correction and normalization of the 1D diffraction intensities were carried out to obtain the total scattering structure function, $F(Q)$, which was Fourier transformed to obtain the PDF, $G(r)$ using PDFgetX3 within xPDFsuite.[8,9] The maximum value used in the Fourier transform of the total scattering data was 21.0 Å^{-1} for the synchrotron and 14.5 Å^{-1} for the Mo-Kα_1 laboratory data. Simulated PDFs were generated from respective models using code from Diffpy-CMI.[10]

S5.2 XPDF analysis of truncated-octahedral c-(4-12)MTBC-Zr_6

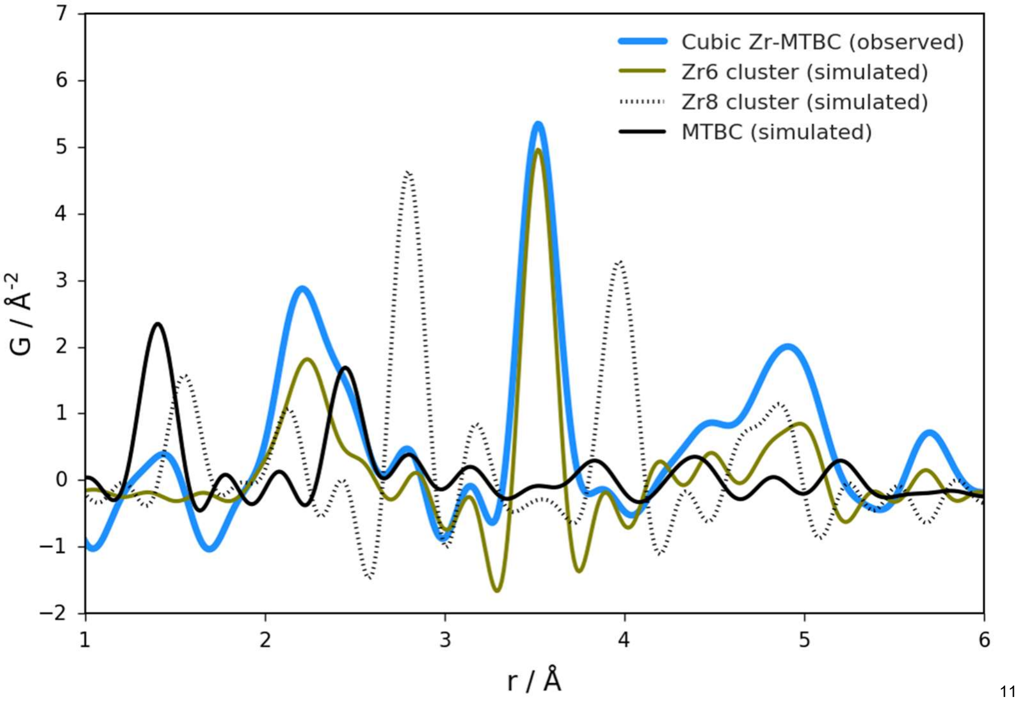

Figure S5.2.1. Comparison of the pair distribution functions (PDFs) of sample c-(4,12)MTBC-Zr_6 (cubic Zr-MTBC) to PDFs simulated for Zr_6 cluster, Zr_8 cluster, and the MTBC linker.[2]

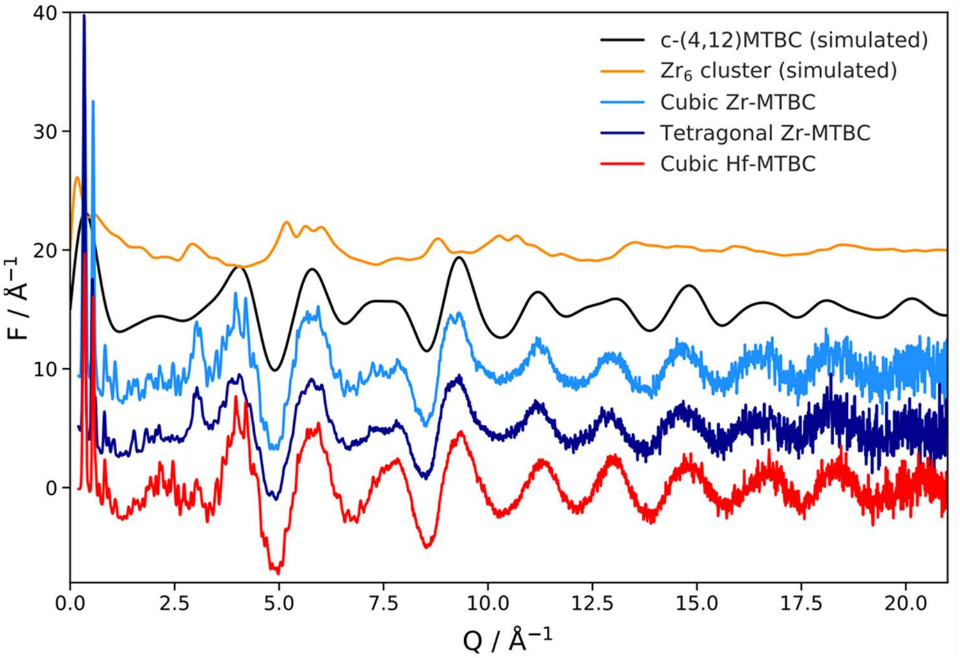

Figure S5.2.2. The reduced total scattering structure functions, $F(Q)$, are shown for several different samples, highlighting the diffuse scattering becoming dominant already around approximately 2.0 Å^{-1}. For comparison, we have simulated the reduced structure functions for a single MTBC linker and a single Zr_6 cluster with all core and surface oxygens present. This shows that the diffuse scattering is dominated by the secondary building units.

S6. Single crystal X-ray diffraction (SCXRD)

S6.1. Data collection, processing and general modelling details

SCXRD data for c-(4,12)MTBC-Zr_6, tr-(4,12)MTBC-Zr_6, c-(4,12)MTBC-Hf_6, and tr-(4,12)MTBC-Hf_6 were collected at the Bessy Synchrotron facility of the Helmholtz Zentrum Berlin (Beamline 14.2). Suitable crystals were isolated and mounted on a MiTeGen loop in a droplet of a perfluoropolyether oil. Diffraction data were acquired using a monochromatic 0.799897 Å radiation at a temperature of 100K, using a cold dry nitrogen stream produced with an Oxford Cryostream. The crystal was placed directly under the Cryostream at 100K without any temperature ramp in going from room temperature to cryogenic conditions. Frames were collected on a Pilatus3S 2M, running a phi scan with oscillation 0.2° and covering an angular range of 360°. Data from c-(4,12)MTBC-Zr_6/Hf_6 were processed using the software XDS,[12] which included indexing, intensities integration and empirical absorption correction. Data from tr-(4,12)MTBC-Zr_6/Hf_6 were processed by the software suite CrysalisPro (ver 41.93a),[13] which was used for unit cell determination, intensities integration, and empirical absorption correction. Structure solution and refinement were conducted by using the software suite Olex2 v1.5,[14] using the programs ShelXT (intrinsic phasing) and ShelXL (least squares) respectively. [15]

As far as structure modelling for refinement is concerned, a few restraints helped maintaining chemically sound bond distances and molecular geometries during the modelling of structural disorder. Specifically, phenyl rings were constrained to a flat hexagonal geometry by AFIX66, and carboxylate moieties of the linkers were modelled in reference geometries using DFIX and DANG commands. Similarly, ISOR and SIMU ADP constraints were used to guide misbehaving displacement parameters towards more physically sound values, as it was observed that free refinement of displacement ellipsoids led to behaviour inconsistent with a meaningful physically sound situation. Furthermore, hydrogen atoms belonging to OH groups on the clusters or aromatic CH groups were fixed by appropriate ShelX commands and were not refined freely.

Every sample was found as a single crystal, and no twinning was detected in the data or in the reciprocal space maps. In the final stages of the refinement, the tool "Mask" of Olex2 (alternative to SQUEEZE which is implemented in Platon)

was used to account for solvent molecules, assumed as N,N'-dimethylformamide, which were not possible to recognise and model satisfactorily in the residual densities.

Symmetry-independent building blocks were refined with free occupancies to account for missing-building-block defects. When occupancy factors exceeded unity, they were fixed to full occupancy. Additional details for each single crystal are reported below in dedicated sections. Partial linker occupancies in the cubic phases were found to coincide with the presence of unaccounted electron density residues in proximity to metal centres, consistent with the presence of water molecules and OH- groups to ensure overall charge balance of the framework. Therefore, these species were modelled and their occupancy was set to complement those of the linker molecules, so that the sum of the occupancies of linkers and H_2O/OH pairs ensures full occupancy on each coordination position around the metal clusters.

Disordered $Zr_6O_4(OH)_4$ SBUs were modelled without oxo- or hydroxy groups due to excessive overlap of electron densities in the observed data. This created smeared electron density maxima in positions that were not compatible with chemically sound presence of these species. On the other hand, fixing the presence of oxygen and hydroxy groups in idealised positions resulted in strong increase of R1% factors, further signal of inconvenient overfitting. Thus, we opted for not modelling these atoms and instead providing a more clear model of the only metallic component of the clusters.

Difference Fourier maps were computed by using the software Vesta[16] by the following routine:

1) A complete model including structure disorder was refined until convergence, until the structure used for deposition into the Cambridge Structural Database is obtained, which includes the use of solvent mask.
2) The disordered atoms relative to the electron density of interest are removed.
3) A single least square ShelXL refinement cycle is ran, using the command list3 in the ".ins" file, which allows to obtain a ".fcf" file compatible with Vesta.
4) The structure model is loaded in vesta, and the Fourier difference map is generated by the function "Fourier synthesis" and the option "Fo-Fc".
5) Positive residues only are selected in the graphical options, and multi-level isosurfaces are coloured to obtain an informative and clear qualitative picture of the electron density residues due to unmodelled disordered building blocks.

S6.2. SCXRD refinement details

Truncated-octahedral c-(4,12)MTBC-Zr_6 crystal nr.1
Linker occupancy: 0.901(3); **Ordered cluster occupancy**: 1; **Disordered cluster occupancy**: 0.25

Solvent masking accounted for electron density peaks that were not possible to assign to discrete solvent molecules without deteriorating the quality of the refinements, due to the overlap of symmetry-equivalent positions of solvent species (likely *N,N'*-dimethylformamide). Difference Fourier maps were computed for displaying the density residues, as shown in Figure S6.2.2.

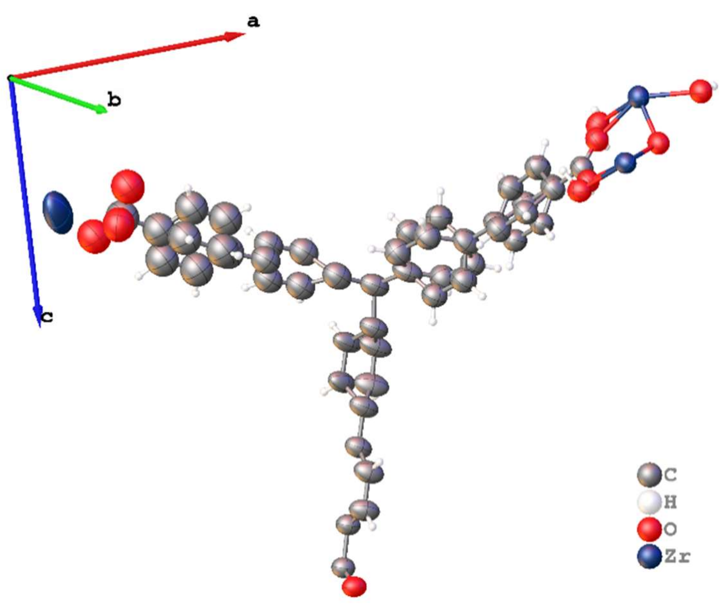

Figure S6.2.1. Asymmetric unit of truncated-octahedral c-(4,12)MTBC-Zr_6 crystal nr.1 (ellipsoids corresponding to 30% probability).

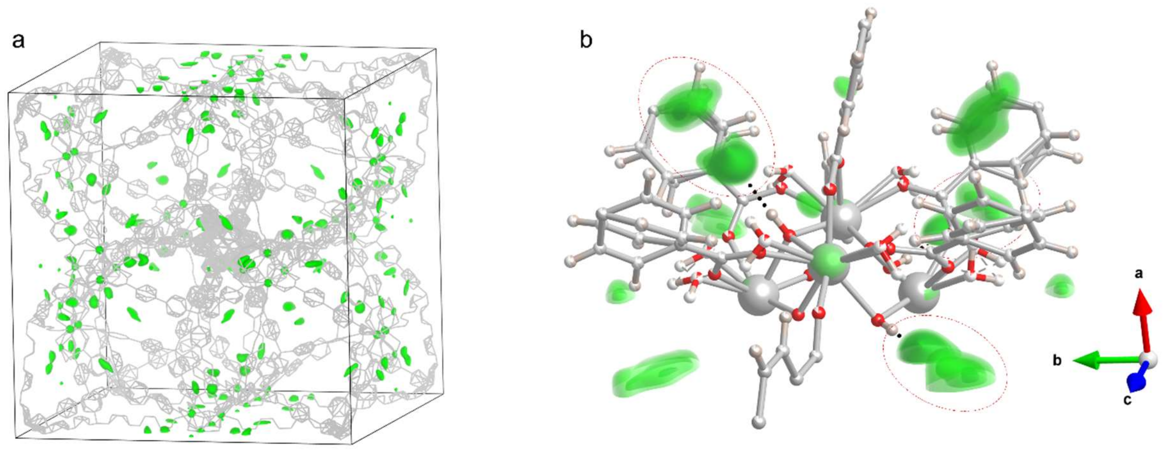

Figure S6.2.2. Difference electron density maps (Fobs-Fcalc) obtained by using the option "Fourier Synthesis" of the software Vesta, based on the ".fcf" file produced by structure refinements without solvent mask. In detail, (a) view of the electron density residues in the unit cell (isosurface level = 1 $e^-/Å^3$), and (b) main location where these densities are found, i.e., around the ordered metal clusters (isosurface levels = 0.8, 1, and 1.2 $e^-/Å^3$). Note the circled areas, where density maxima suggest the presence of solvent molecules interacting with the cluster's OH groups via hydrogen bonding (dotted black line; H–density maximum distance: 1.939(4) Å).

Truncated-octahedral c-(4,12)MTBC-Zr_6 crystal nr.2
Linker occupancy: 0.946(3); **Ordered cluster occupancy**: 1; **Disordered cluster occupancy**: 0.2365(7)

Solvent masking produced electron densities without significant differences compared to those shown for crystal nr.1 in Figure S6.2.2.

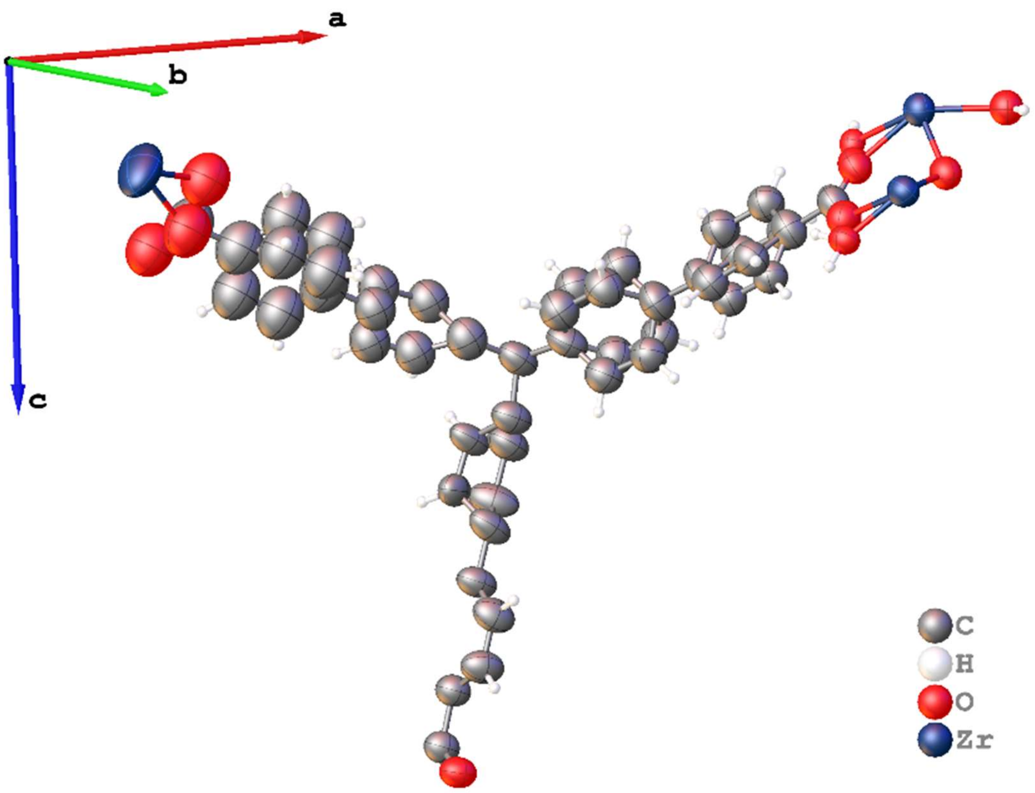

Figure S6.2.3. Asymmetric unit of truncated-octahedral c-(4,12)MTBC-Zr_6 crystal nr.2 (ellipsoids corresponding to 30% probability).

Table S6.2.1. Crystal data and structure refinement for truncated-octahedral c-(4,12)MTBC-Zr_6 crystals.

	**Crystal nr. 1**	**Crystal nr. 2**
**Empirical formula**	$C_{143.3}H_{88.3}O_{28.82}Zr_6$	$C_{150.39}H_{94.78}O_{29.35}Zr_6$
**Formula weight**	2818.44	2911.27
**Temperature/K**	100	100
**Crystal system**	cubic	cubic
**Space group**	Pm-3n	Pm-3n
**a/Å**	41.510(10)	41.48(5)
**Volume/Å^3**	71525(52)	71344(258)
**Z**	8	8
**ρ_{calc}g/cm^3**	0.523	0.542
**μ/mm^{-1}**	0.270	0.269
**F(000)**	11349.0	11750.0
**Crystal size/mm^3**	0.15 × 0.15 × 0.15	0.15 × 0.15 × 0.15
**Radiation**	synchrotron (λ = 0.79999)	Synchrotron (λ = 0.79999)
**2Θ range /°**	3.124 to 64.352	2.708 to 64.4
**Index ranges**	-55 ≤ h ≤ 54, -55 ≤ k ≤ 55, -55 ≤ l ≤ 55	-52 ≤ h ≤ 52, -55 ≤ k ≤ 55, -55 ≤ l ≤ 55
**Reflections collected**	992517	1001027
**Independent reflections**	15063 [R_{int} = 0.0516, R_{sigma} = 0.0099]	14973 [R_{int} = 0.0560, R_{sigma} = 0.0111]

**Data/restraints/param.**	15063/368/519	14973/383/522
**Goodness-of-fit on F^2**	1.256	1.260
**Final R indexes [I>=2σ (I)]**	R_1 = 0.0979, wR_2 = 0.3051	R_1 = 0.1066, wR_2 = 0.3287
**Final R indexes [all data]**	R_1 = 0.1156, wR_2 = 0.3497	R_1 = 0.1318, wR_2 = 0.3831
**Largest diff. peak/hole / e Å^{-3}**	1.77/-0.71	1.79/-0.76

Cube-shaped c-(4,12)MTBC-Zr_6 crystal nr.1

Linker occupancy: 0.932(2); **Ordered cluster occupancy**: 1, **Disordered cluster occupancy, Zr3**: 0.1810(16); **Disordered cluster occupancy, Zr4**: 0.167(3)

The two Zr occupancies were refined to have an overall occupancy of the disordered cluster of 1, since free occupancy refinements resulted in an overall site occupancy >1.

Solvent masking produced electron densities without significant differences compared to those shown in Figure S6.2.2.

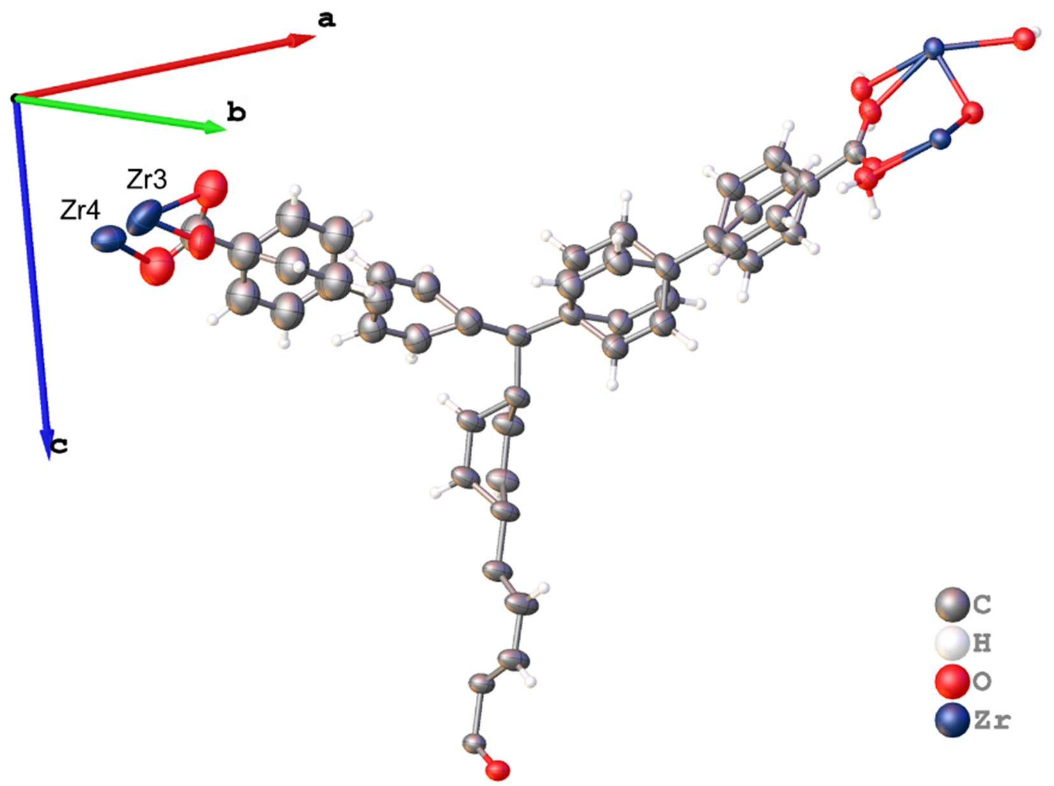

Figure S6.2.4. Asymmetric unit of cubic c-(4,12)MTBC-Zr_6 crystal nr.1 (ellipsoids corresponding to 30% probability).

Cube-shaped c-(4,12)MTBC-Zr_6 crystal nr.2

Linker occupancy: 0.931(2); **Ordered cluster occupancy**: 1; **Disordered cluster occupancy**, **Zr3**: 0.1851(16) **Disordered cluster occupancy**, **Zr4**: 0.167(3)

The two Zr occupancies were refined to have an overall occupancy of the disordered cluster of 1, since free occupancy refinements resulted in an overall site occupancy >1. Solvent masking produced electron densities without significant differences compared to those shown in Figure S6.2.2.

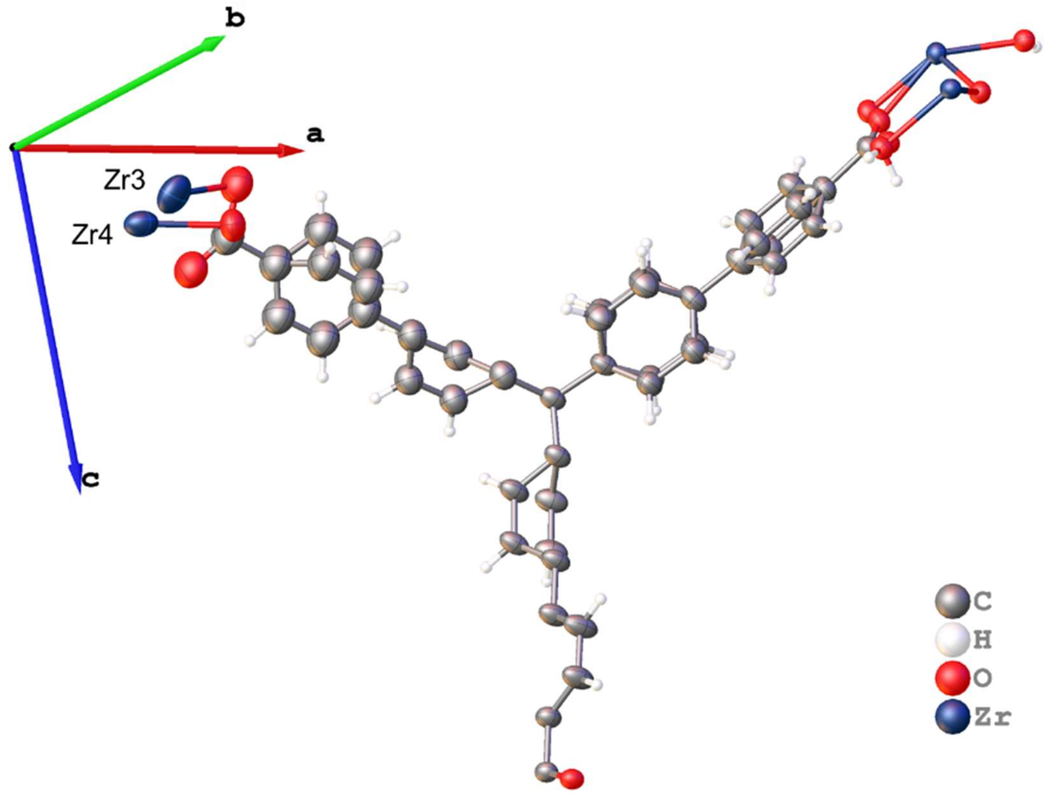

Figure S6.2.5. Asymmetric unit of cubic c-(4,12)MTBC-Zr_6 crystal nr.2 (ellipsoids corresponding to 30% probability).

Table S6.2.2. Crystal data and structure refinement for cubic c-(4,12)MTBC-Zr_6 crystals.

	**Crystal nr. 1**	**Crystal nr. 2**
**Empirical formula**	$C_{147.64}H_{93.43}O_{29.14}Zr_6$	$C_{147.44}H_{93.33}O_{29.13}Zr_6$
**Formula weight**	2879.73	2878.20
**Temperature/K**	100	100
**Crystal system**	cubic	cubic
**Space group**	Pm-3n	Pm-3n
**a/Å**	41.630(10)	41.646(4)
**Volume/Å^3**	72147(52)	72232(22)
**Z**	8	8
**ρ_{calc}g/cm^3**	0.530	0.529
**μ/mm^{-1}**	0.269	0.268
**F(000)**	11614.0	11608.0
**Crystal size/mm^3**	0.15 × 0.15 × 0.15	0.15 × 0.15 × 0.15
**Radiation**	Synchrotron (λ = 0.79999)	Synchrotron (λ = 0.79999)
**2Θ range /°**	2.698 to 64.426	2.696 to 64.422
**Index ranges**	-52 ≤ h ≤ 52, -55 ≤ k ≤ 55, -55 ≤ l ≤ 55	-52 ≤ h ≤ 52, -55 ≤ k ≤ 55, -55 ≤ l ≤ 55
**Reflections collected**	1022290	1004180

**Independent reflections**	15138 [R_{int} = 0.0431, R_{sigma} = 0.0083]	15024 [R_{int} = 0.0398, R_{sigma} = 0.0085]
**Data/restraints/parameters**	15137/816/565	15024/816/565
**Goodness-of-fit on F^2**	1.072	1.067
**Final R indexes [I>=2σ (I)]**	R_1 = 0.0731, wR_2 = 0.2390	R_1 = 0.0736, wR_2 = 0.2393
**Final R indexes [all data]**	R_1 = 0.0788, wR_2 = 0.2536	R_1 = 0.0790, wR_2 = 0.2530
**Largest diff. peak/hole / e Å^{-3}**	1.81/-1.76	1.82/-1.78

Truncated-octahedral c-(4,12)MTBC-Hf_6 crystal nr.1

Linker occupancy: 0.885(5); **Ordered cluster occupancy**: 1; **Disordered cluster occupancy**: 0.25
Solvent masking produced electron densities without significant differences compared to those shown in Figure S6.2.2.

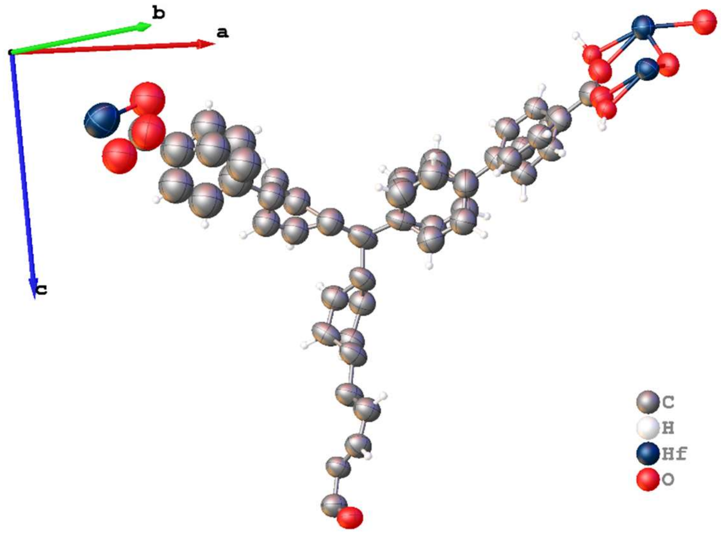

Figure S6.2.6. Asymmetric unit of truncated-octahedral c-(4,12)MTBC-Zr_6 crystal nr.1 (ellipsoids corresponding to 30% probability).

Table S6.2.3. Crystal data and structure refinement for c-(4,12)MTBC-Hf_6 crystals.

	**Crystal nr. 1**	**Crystal nr. 2**
**Empirical formula**	$C_{145.16}H_{92.21}Hf_6O_{28.96}$	$C_{138.65}H_{89.02}Hf_6O_{28.46}$
**Formula weight**	3374.63	3281.48
**Temperature/K**	100.00	100.00
**Crystal system**	cubic	cubic
**Space group**	Pm-3n	Pm-3n
**a/Å**	41.41(5)	41.46(6)
**Volume/Å^3**	71030(247)	71257(320)
**Z**	8	8
**ρ_{calc}g/cm^3**	0.631	0.612
**μ/mm^{-1}**	2.406	2.388
**F(000)**	13028.0	12646.0
**Crystal size/mm^3**	0.15 × 0.15 × 0.15	0.15 × 0.15 × 0.15

**Radiation**	Synchrotron (λ = 0.79999)	Synchrotron (λ = 0.79999)
**2Θ range for data collection/°**	2.712 to 47.082	3.128 to 59.942
**Index ranges**	-40 ≤ h ≤ 40, -41 ≤ k ≤ 41, -41 ≤ l ≤ 41	-51 ≤ h ≤ 51, -51 ≤ k ≤ 51, -51 ≤ l ≤ 51
**Reflections collected**	445098	856804
**Independent reflections**	6136 [R_{int} = 0.0477, R_{sigma} = 0.0102]	11997 [R_{int} = 0.0436, R_{sigma} = 0.0077]
**Data/restraints/parameters**	6136/872/544	11997/824/553
**Goodness-of-fit on F^2**	1.526	1.052
**Final R indexes [I>=2σ (I)]**	R_1 = 0.0843, wR_2 = 0.2911	R_1 = 0.0587, wR_2 = 0.1961
**Final R indexes [all data]**	R_1 = 0.0923, wR_2 = 0.3239	R_1 = 0.0721, wR_2 = 0.2283
**Largest diff. peak/hole / e Å^{-3}**	2.17/-1.23	1.11/-1.01

Truncated-octahedral c-(4,12)MTBC-Hf_6 crystal nr.2

Linker occupancy: 0.872(3); **Ordered cluster occupancy**: 1; **Disordered cluster occupancy**: 0.25
Solvent masking produced electron densities without significant differences compared to Figure S6.2.2.

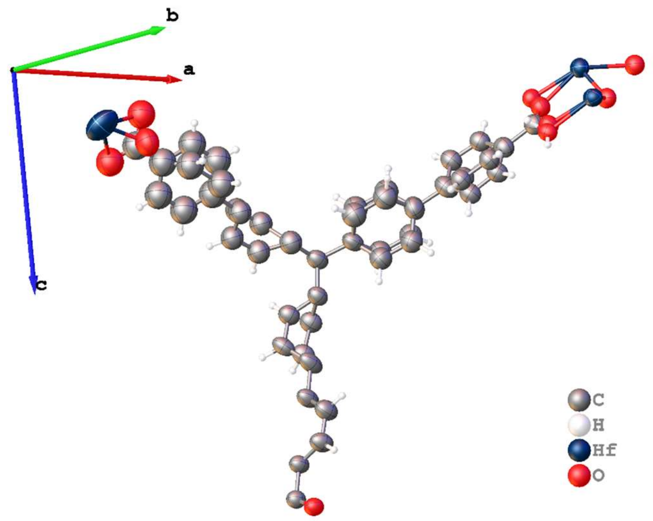

Figure S6.2.7. Asymmetric unit of truncated-octahedral c-(4,12)MTBC-Zr_6 crystal nr.2 (ellipsoids corresponding to 30% probability).

tr-(4,12)MTBC-Zr_6

The structure is affected by disorder in the form of two alternative framework structures, whose combination is observed in the average structure. Consistently, reciprocal space reconstructions reported in **Figure S6.2.10** show streaks and additional intensities along a* and b*, attributable to loss of periodicity in the ab plane of the crystal. To rule out artefacts due to pseudomerohedral twinning or space group symmetry, the structure has been solved and refined in P1, showing the same framework disorder observed in the average structure in P-3c1 settings. An inversion twinning law has been tested on the P1 model, providing refinements where electron densities due to the two alternative framework components were unaffected.

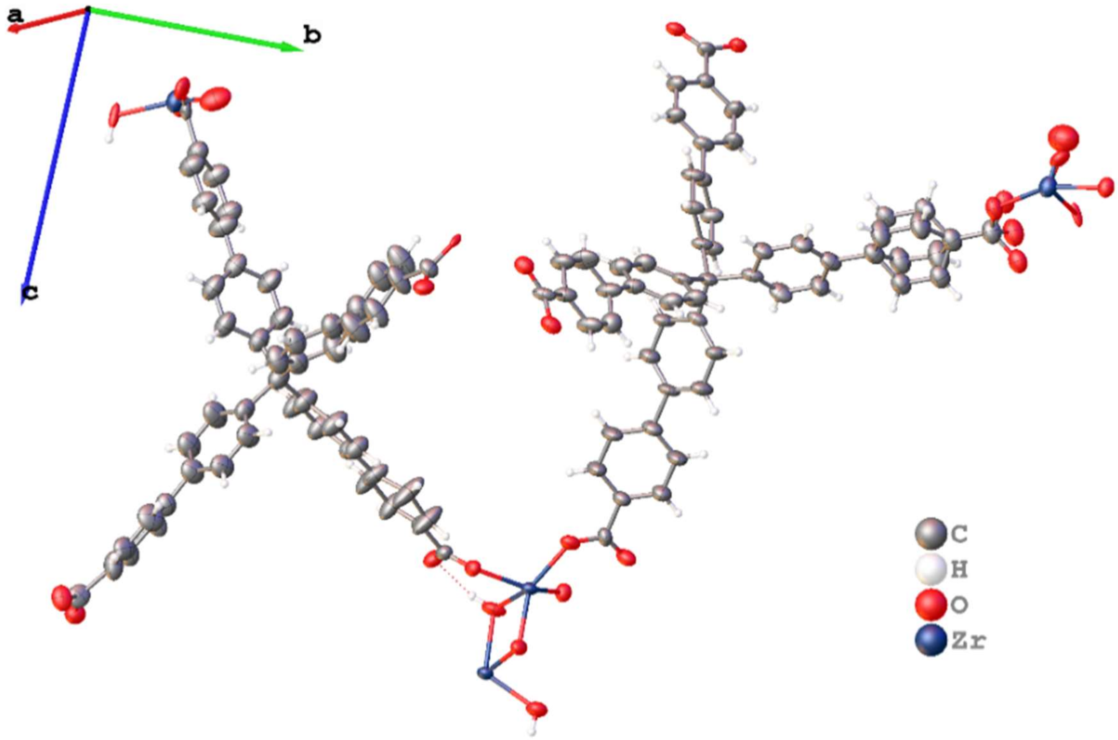

Figure S6.2.8. Asymmetric unit of tetragonal tr-(4,12)MTBC-Zr_6 (ellipsoids corresponding to 30% probability).

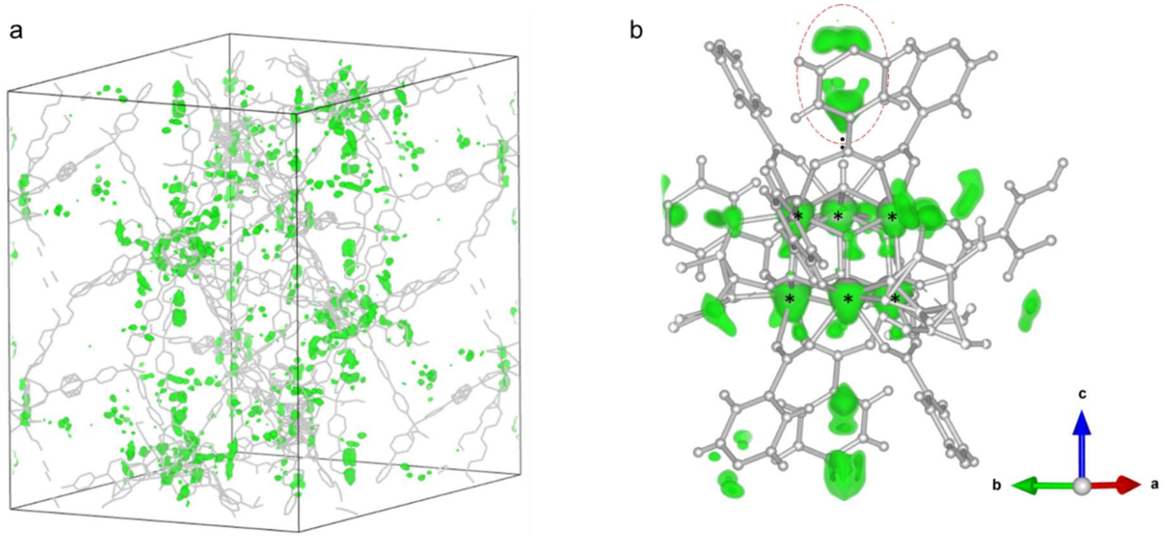

Figure S6.2.9. Difference electron density maps (Fobs-Fcalc) obtained by using the option "Fourier Synthesis" of the software Vesta, based on the ".fcf" file produced by structure refinements without solvent mask. In detail, (a) view of the electron density residues in the unit cell (isosurface level = 1 $e^-/Å^3$), and (b) main location where these densities are found, i.e., around the ordered metal clusters (isosurface levels = 1, 1, and 1.3 $e^-/Å^3$). Note the circled area, where density maxima suggest the presence of solvent molecules interacting with the cluster's OH groups via hydrogen bonding (dotted black line; H–density maximum distance: 1.95(2) Å).

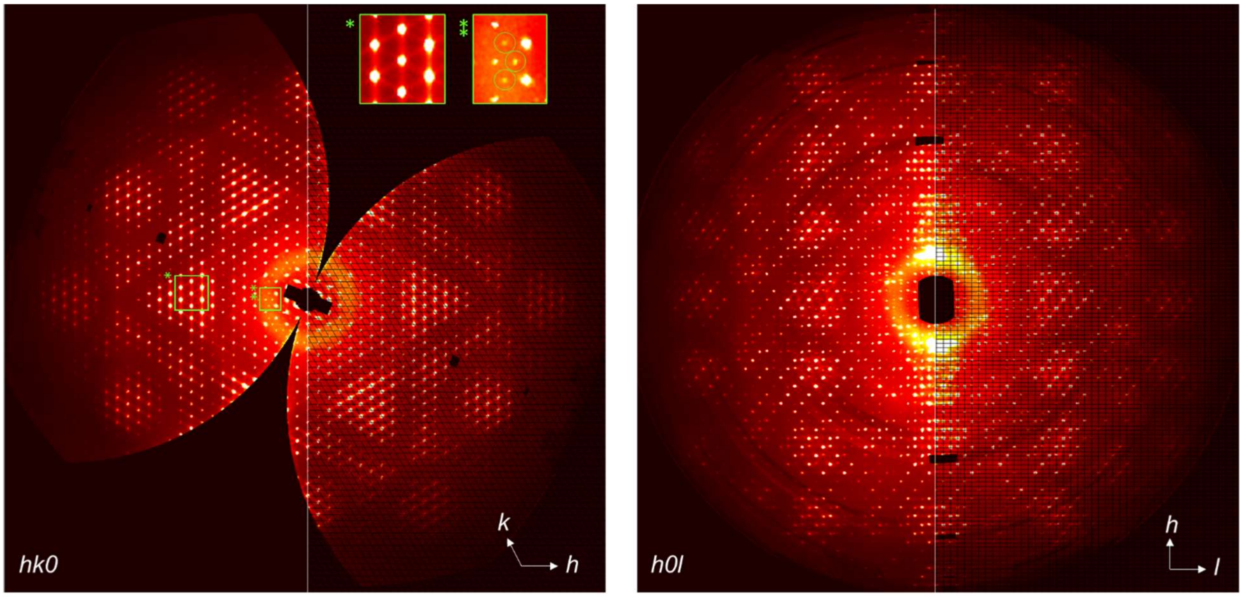

Figure S6.2.10. Reciprocal space reconstructions of the *hk*0 and h0l planes of tr-(4,12)MTBC-Zr_6. Showing diffuse streaks along h and k (*) due to loss of periodicity across the ab plane of the crystal, violation of systematic absences attributable to substitutional disorder (**), and a peculiar Bragg peaks distribution which does not match perfectly the unit cell metric, akin to an incommensurately modulated structure.

tr-(4,12)MTBC-Hf_6

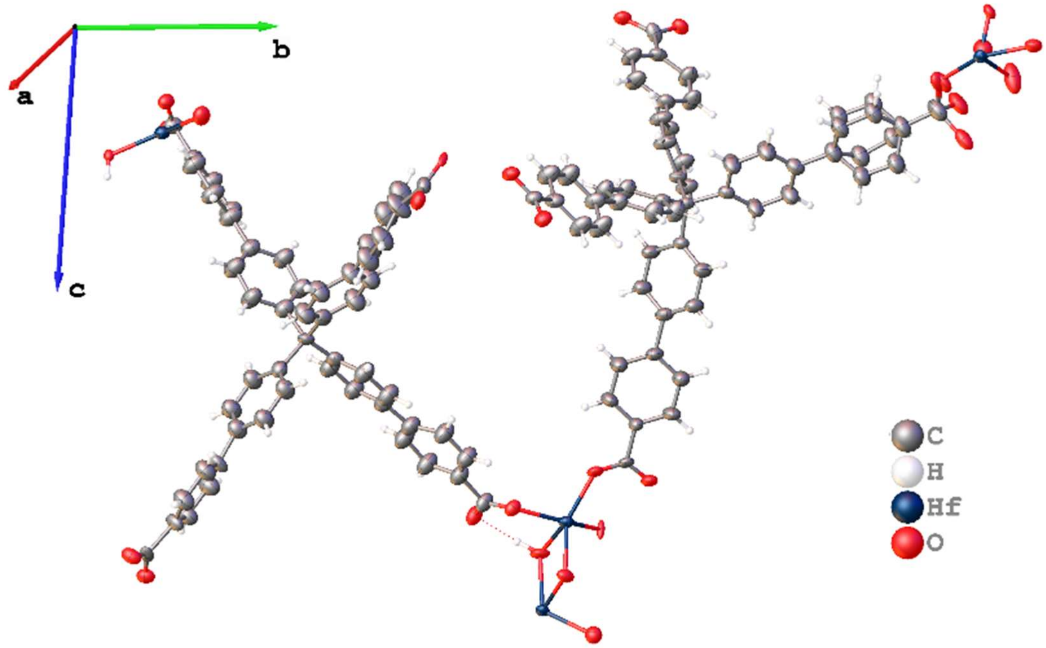

Figure S6.2.11. Asymmetric unit of tetragonal tr-(4,12)MTBC-Hf_6 (ellipsoids corresponding to 30% probability).

Table S6.2.4. Crystal data and structure refinement for tr-(4,12)MTBC-Zr_6 and tr-(4,12)MTBC-Hf_6.

**Empirical formula**	$C_{159}H_{97}O_{33.5}Zr_6$	$C_{159}H_{98.33}O_{34}Hf_6$
**Formula weight**	3090.68	3623.64
**Temperature/K**	100	100
**Crystal system**	trigonal	trigonal
**Space group**	P-3c1	P-3c1
**a/Å**	34.3901(4)	35.0368(4)
**c/Å**	49.4392(4)	48.7688(4)
**α/°**	90	90
**γ/°**	120	120
**Volume/Å^3**	50637.1(12)	51846.7(13)
**Z**	6	6
**ρ_{calc}g/cm^3**	0.608	0.696
**μ/mm^{-1}**	0.291	0.897
**F(000)**	9354.0	10538.0
**Crystal size/mm^3**	0.15 × 0.15 × 0.04	0.15 × 0.15 × 0.04
**Radiation**	Synchrotron (λ = 0.79999)	Synchrotron (λ = 0.534)
**2Θ range for data collection/°**	3.854 to 56.142	2.948 to 38.992
**Index ranges**	-40 ≤ h ≤ 40, -37 ≤ k ≤ 37, -58 ≤ l ≤ 58	-43 ≤ h ≤ 43, -42 ≤ k ≤ 43, -60 ≤ l ≤ 60
**Reflections collected**	507581	685321
**Independent reflections**	28787 [R_{int} = 0.0930, R_{sigma} = 0.0251]	35355 [R_{int} = 0.0807, R_{sigma} = 0.0299]
**Data/restraints/parameters**	28787/570/1074	35355/1290/1151
**Goodness-of-fit on F^2**	1.697	1.034
**Final R indexes [I>=2σ (I)]**	R_1 = 0.1343, wR_2 = 0.3955	R_1 = 0.0654, wR_2 = 0.1769
**Final R indexes [all data]**	R_1 = 0.1671, wR_2 = 0.4440	R_1 = 0.1003, wR_2 = 0.2126
**Largest diff. peak/hole / e Å^{-3}**	4.58/-1.40	2.40/-1.65

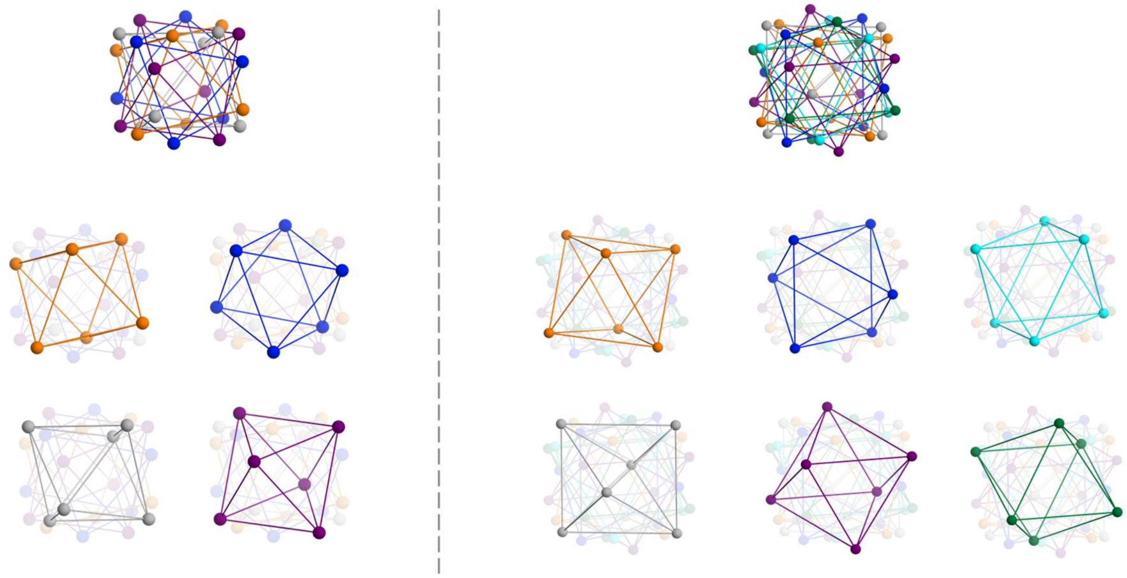

Figure S6.2.12. Deconstruction of the two kinds of cluster disorder found in truncated-octahedral and cube-shaped c-(4,12)MTBC-Zr_6 and (left and right, respectively).

S6.3. 3D electron density maps availability

Electron density maps can be consulted as Vesta files, which are provided as supplementary material. Each deposited structure was used to compute an electron density map by the function "*Fourier synthesis…*" in the software Vesta using the observed structure factors (Fo). For each structure, two necessary files are provided: a "*.vesta*" and a "*.pgrid*" file. Both files need to be present in the same folder, in which case it is possible to open the "*.vesta*" file using Vesta. At this point, an electron density map will be visible as displayed in the figure S6.3.1. By appropriately selecting data cut-off planes, it is possible to limit the view to specific features of interest, as shown in Figure S6.3.2. It is also possible to change the visualisation of the electron densities by changing the values of the isosurface levels in the dedicated tab (*style* -> *properties* -> *isosurfaces*). The atomic model used to fit the electron densities can be overlayed by simple ticking the "*show models*" option at the top of the "*Styles*" tab in the main window.

We highly recommend to consider inspecting such maps when conducting crystallographic modelling, and we encourage to provide these files to allow the scientific community to get access to the crystallographic information obtained from X-ray diffraction experiments, which is the electron densities and not the atomic model introduced to fit them.

To produce these files, one can simply load the *.cif* file into Vesta, then select "*Fourier synthesis…*" in the panel *"Utilities"*, click "*Import…*" and select the *.fcf* file produced by structure refinements. At this point, it is necessary to select Fo to visualise the observed densities and not the residues or the fitted ones, the latter being Fo–Fc and Fc respectively. Normally, the table is immediately filled with numbers. If it did not, it means that the fcf file was not suited for Vesta. In this case, one should use the LIST 3 command during ShelXL refinements to produce *.fcf* files compatible with Vesta. If the table is filled correctly, check that there are no non-numerical values associated to some of the reflections, which could cause errors in the calculation of the maps. If present, those reflections should be excluded by selecting and deleting their lines. At this point, clicking "Calculate" will produce the electron density map, which will overlay onto the loaded structure.

This practice can also be used on published structures, provided that the authors included the *hkl* data (reflections list) in the deposited *.cif* files as required by the F.A.I.R. principles of scientific research (findability, accessibility, interoperability and reusability of scientific data). Loading a *.cif* file with embedded reflections in a software for structure solution and refinement such as Olex2 allows to run refinement cycles on the crystallographic model, thereby producing the *.fcf* file necessary to compute the electron density maps.

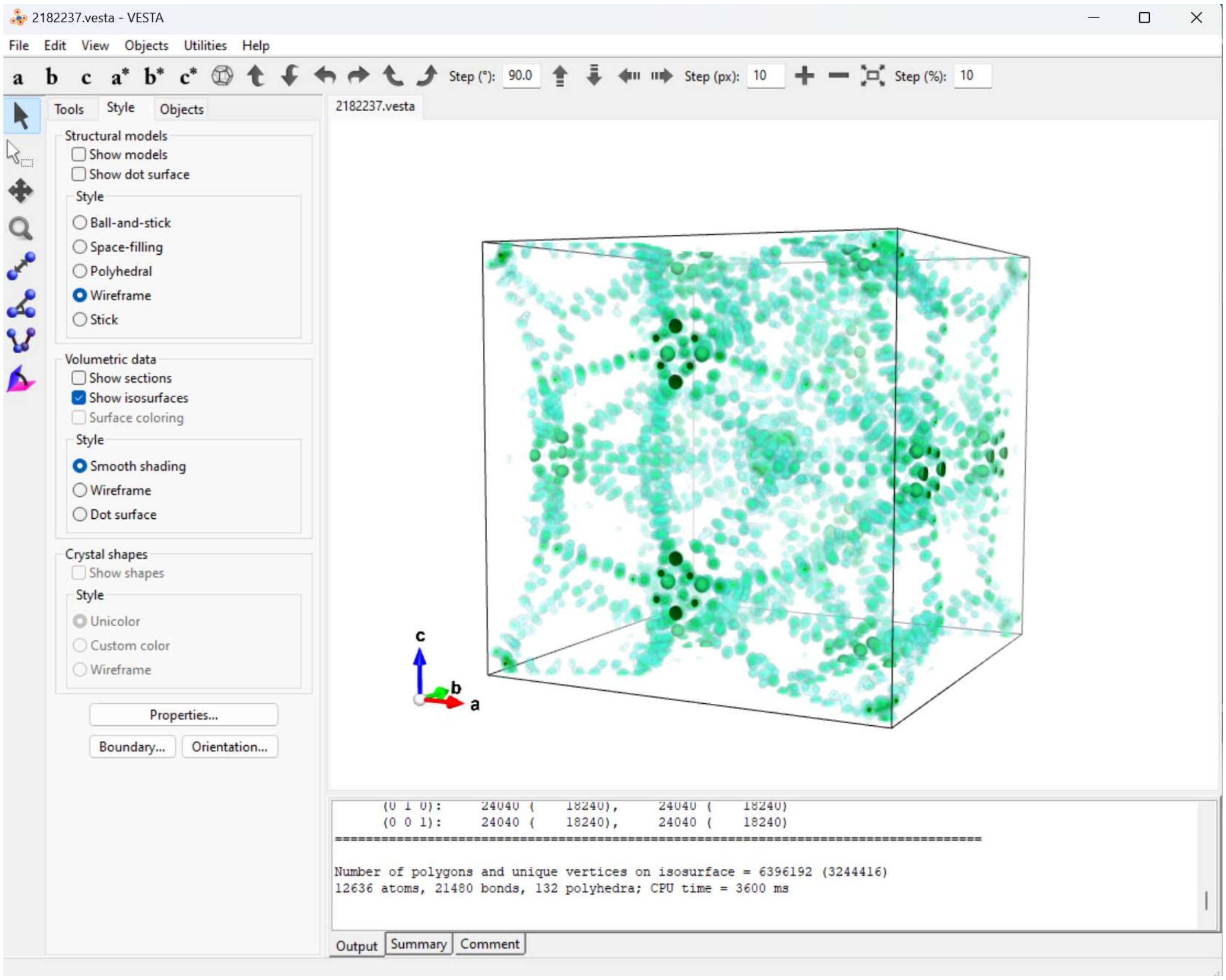

Figure S6.3.1. The Vesta window once the file *2182237.vesta* is loaded (in this case, containing the structure of c-(4,12)MTBC-Zr_6 with cubic crystal morphology).

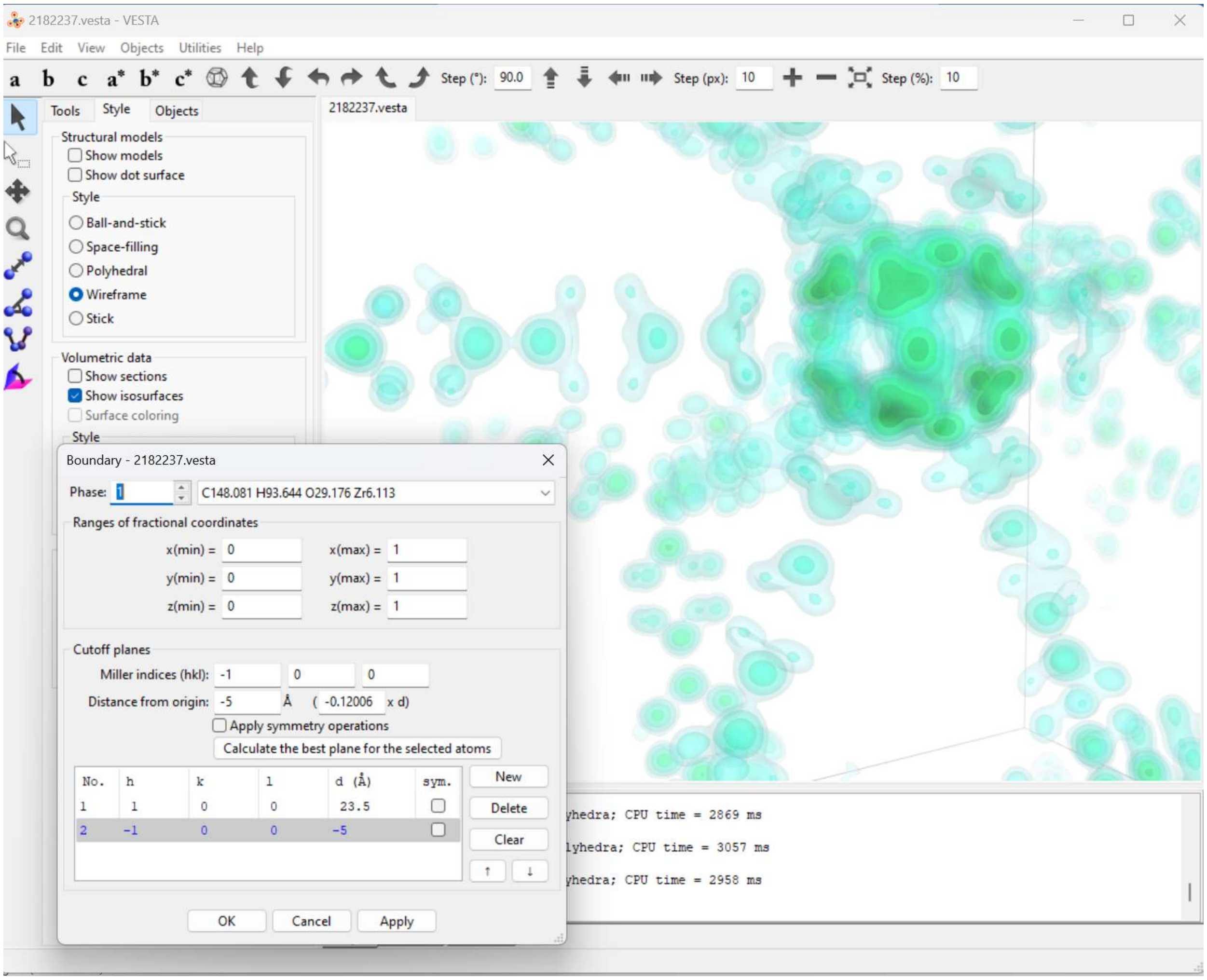

Figure S6.3.2. Vesta allows to set up cutoff planes to ease the visualization of features of interest in electron density maps.

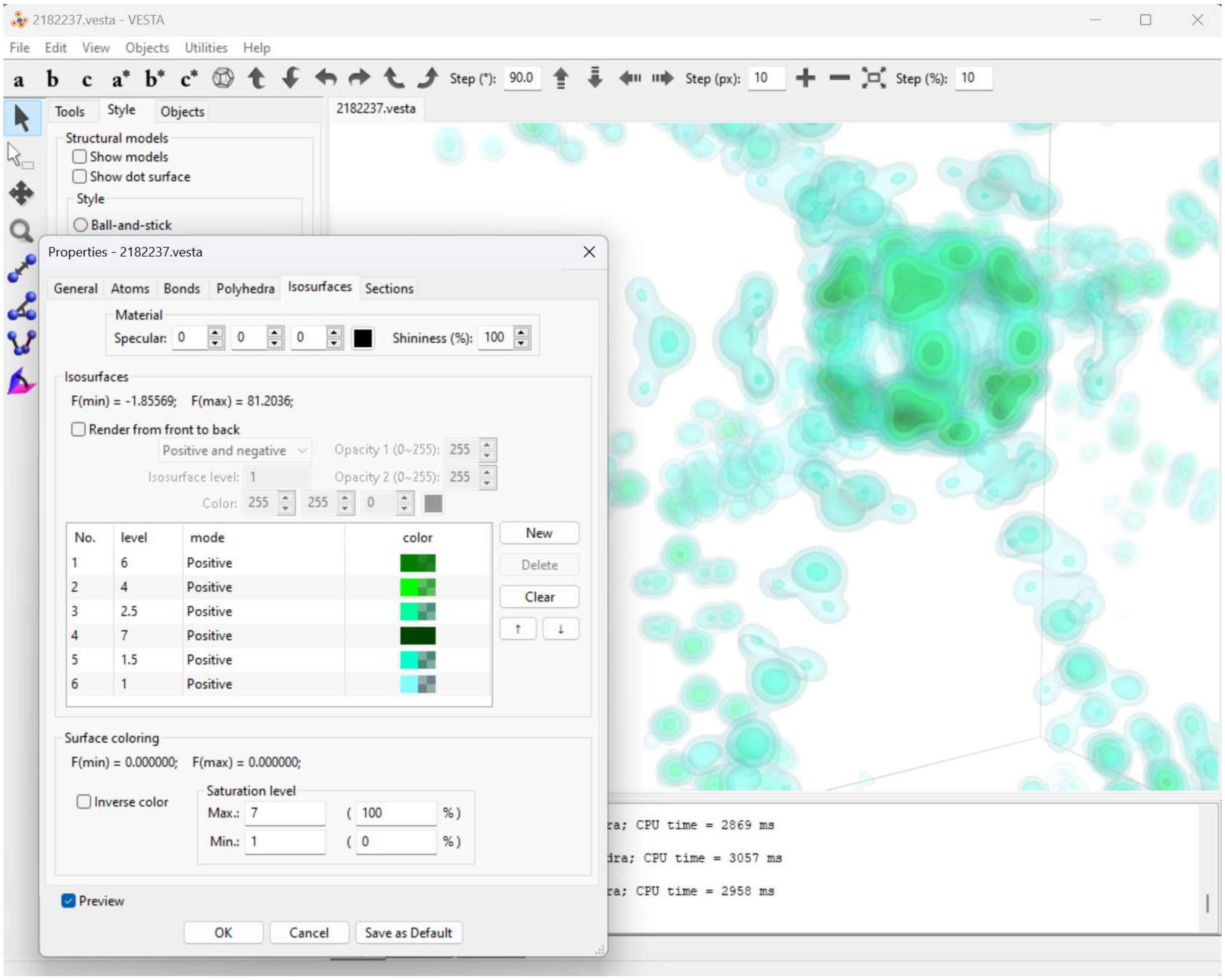

Figure S6.3.3. The isosurfaces' levels and colors can be changed at will, so that features of interest can be enhanced and better inspected. Note that the only positive fraction of Fo was considered to allow for a cleaner visualization of the map. Normally, inspecting both positive and negative components at the same time allows for a more critical estimate of the real crystallographic information, revealing the presence of artefacts that should not be mistaken for meaningful information.

S7. Single crystal diffuse scattering analysis

S7.1. 3D reciprocal space reconstructions and processing

Reciprocal space reconstructions were generated by the following routine:

1) Each dataset was processed by the software XDS[12] to produce a corrected parameter file (GXPARM) containing unit cell and orientation matrix of the crystal.
2) A suitable mask to cover beamstop and undesired regions of the diffraction frames was created by using the calibration tool of the azimuthal integration library pyFAI (pyFAI-calib2),[6] and used for all datasets collected with the same instrumental configuration and settings.
3) Starting, unedited reconstructions were produced by the software Meerkat[17] by setting as maximum h, k, and l 58.5, and number of pixels 1171 to achieve a sampling step of 0.1 reciprocal lattice units.
4) A series of corrections were adopted by using a custom-made python program:
    a) Symmetry averaging has been applied on each reconstruction to improve statistics, decrease noise, and cover missing volumes, using the Laue symmetry of the average structure model (m-3m).
    b) A spherically symmetric background was extracted from the reconstructed diffraction pattern and then subtracted from the symmetry-averaged reconstruction. The background was estimated by calculating, for each Q value of the reconstructed reciprocal space, the mean value of the 1% weakest intensities observed at that Q-value. The choice of the 1% threshold was obtained by comparing the background subtracted patterns obtained using different threshold values, such as 0.5%, 1%, 2%, 5%. The judged the 1% threshold value as sufficient for eliminating most of the visible amorphous background. As no quantitative usage of the diffuse intensities was intended, we did not proceed further with background subtraction optimization. We note, in passing, that this procedure provides a semi-quantitative self-consistent method for the correction of extrinsic as well as Compton scattering contributions to the total scattering.
    c) To prevent artefacts in the 3D PDF function, the intensities below 2.5 and above 58 reciprocal lattice units along each of the main axis were set to zero. This procedure produces a spherical reconstructed reciprocal space.
    d) Each reconstruction was normalized by dividing the value in each voxel by the total integral of the reconstruction. A multiplier (10^{12}) was then applied post-normalization to each reconstruction.

Bragg peaks removal was based on a “punch-and-fill” routine.[18] Since the diffuse intensities are mostly present around the Bragg positions, a spherical mask with fixed radius was applied to a voxel around each Bragg position (effectively “punch” the Bragg intensities). The new intensities on the masked voxels were calculated by interpolating[19] the intensities within a spherical volume centred on the same Bragg position but with a radius twice the mask radius.

3D PDF maps were calculated by applying a Fast Fourier transform (FFT) to the corrected reciprocal space reconstructions, while 3DΔPDF maps were calculated by the same procedure, but on the Bragg-subtracted reconstructions after the punch-and-fill procedure. No further normalization was conducted after Bragg peaks removal or on the PDF maps.

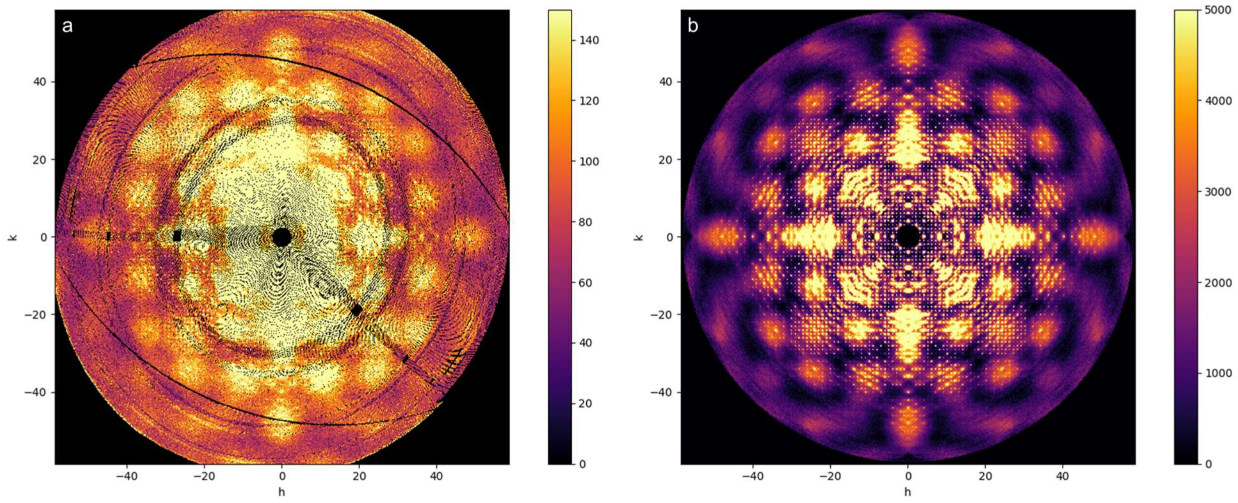

Figure S7.1.1. Pristine (a) and corrected (b) reciprocal space volume of truncated-octahedral c-(4,12)MTBC-Zr_6 crystal nr. 1 (*hk*0 plane).

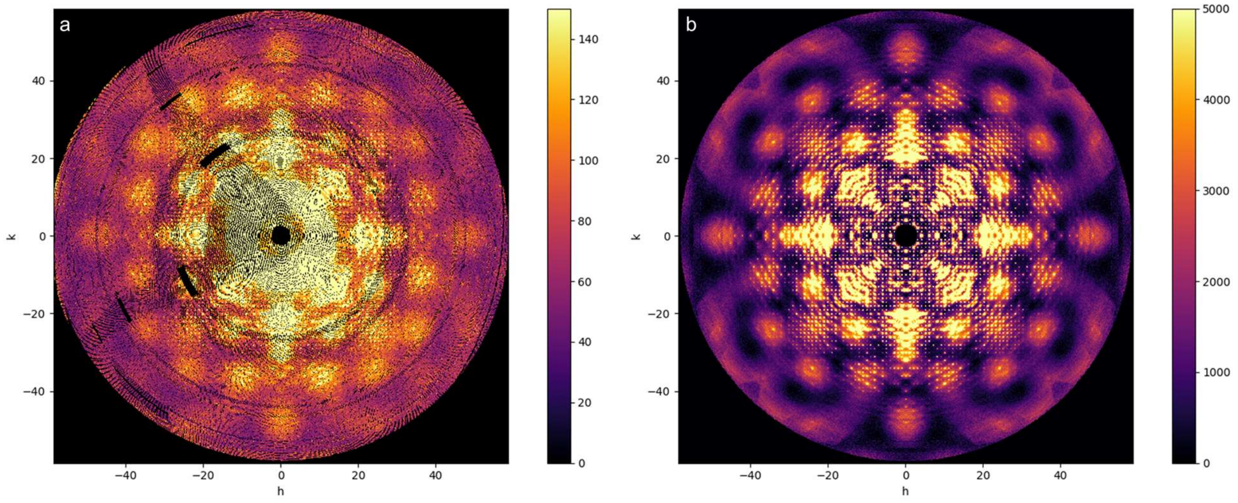

Figure S7.1.2. Pristine (a) and corrected (b) reciprocal space volume of truncated-octahedral c-(4,12)MTBC-Zr_6 crystal nr. 2 (*hk*0 plane).

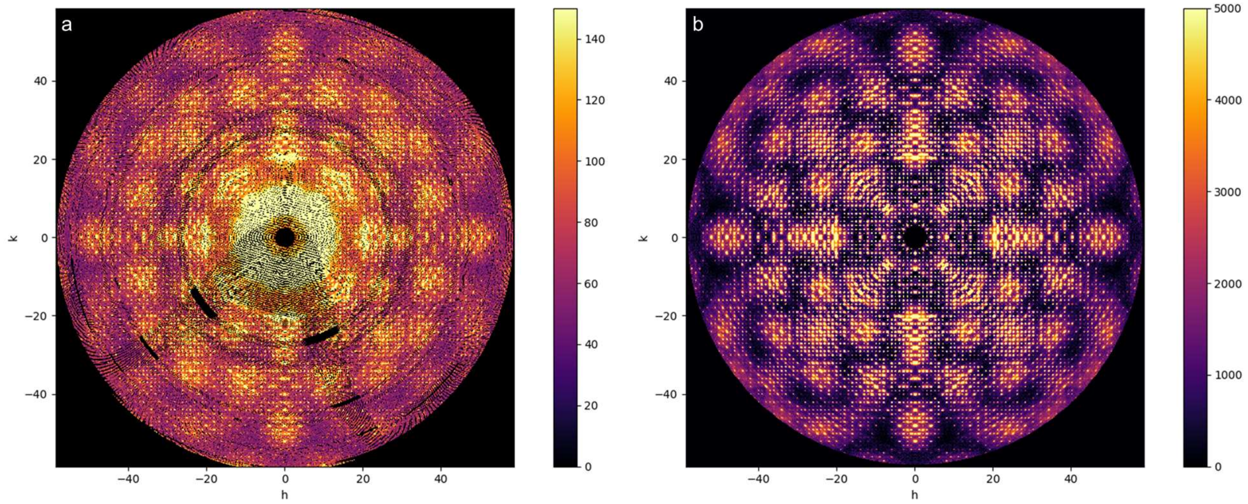

Figure S7.1.3. Pristine (a) and corrected (b) reciprocal space volume of cubic c-(4,12)MTBC-Zr_6 crystal nr. 1 (*hk*0 plane).

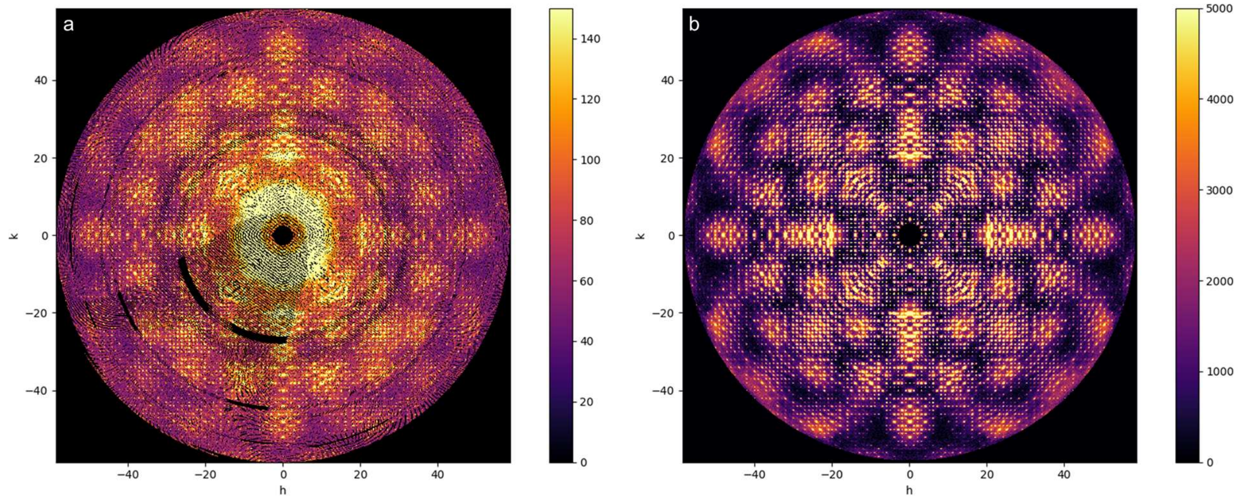

Figure S7.1.4. Pristine (a) and corrected (b) reciprocal space volume of cubic c-(4,12)MTBC-Zr_6 crystal nr. 2 (*hk*0 plane).

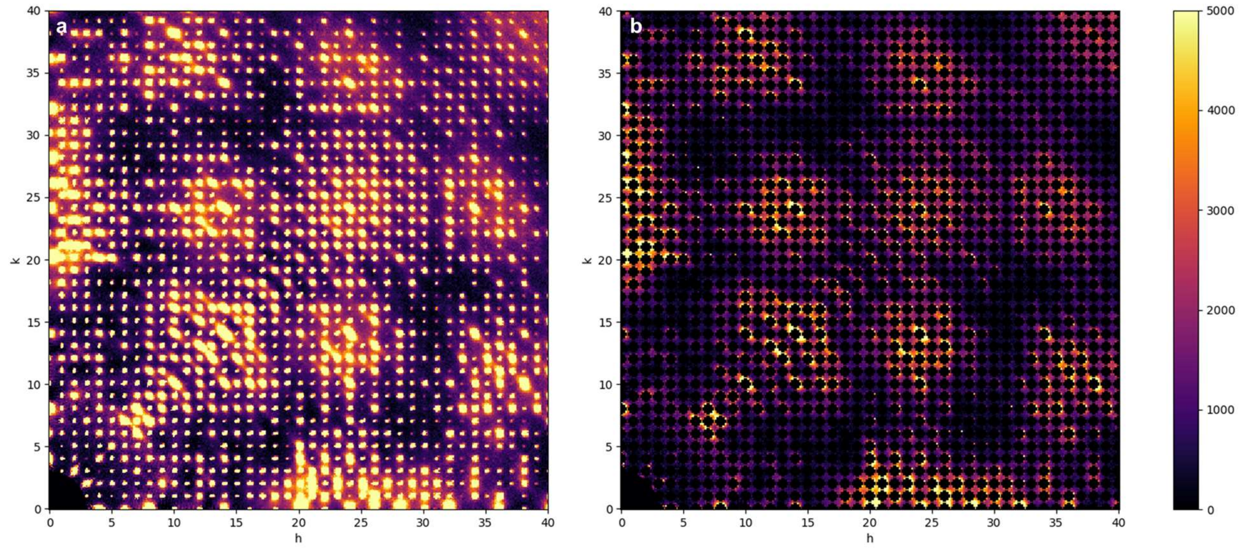

Figure S7.1.5. Exemplary display of the adopted punch radius used in every punch-and-fill procedure, shown in in a selected portion of the *hk*0 plane before (a) and after (b) the punch-only procedure.

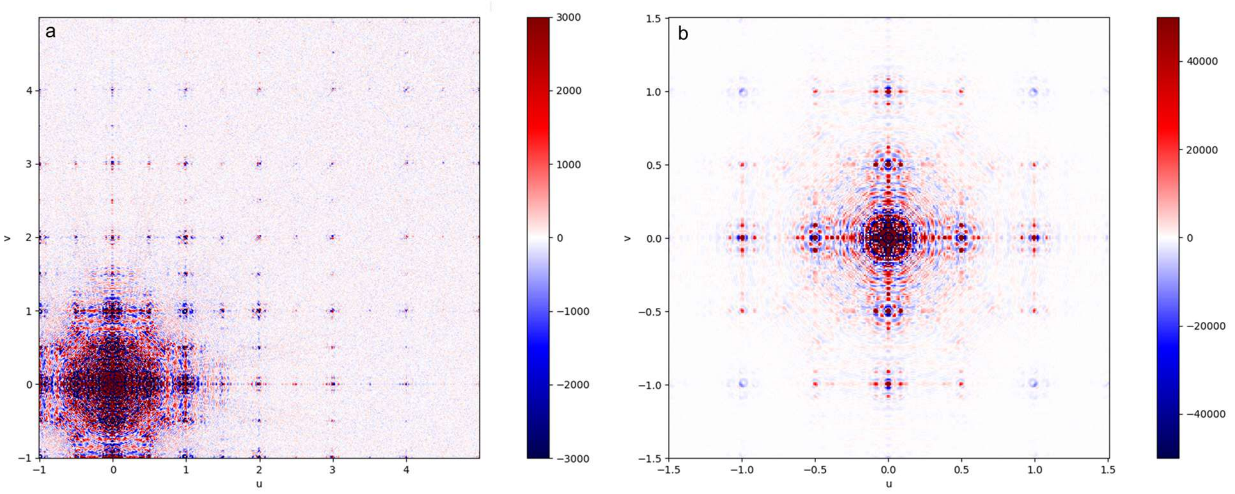

Figure S7.1.6. 3DΔPDF function extracted for truncated-octahedral c-(4,12)MTBC-Zr_6 crystal 1. The uv0 plane with appropriate scales up to 5 unit cell radius (a) or up to 1.5 unit cells radius (b) with appropriate intensity scales.

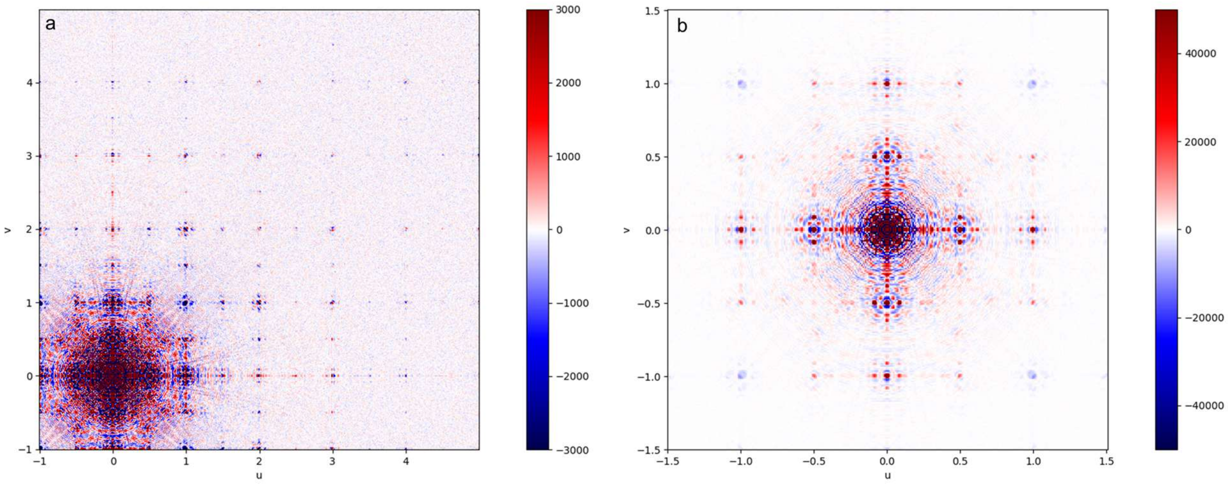

Figure S7.1.7. 3DΔPDF function extracted for truncated-octahedral c-(4,12)MTBC-Zr_6 crystal 2. The uv0 plane with appropriate scales up to 5 unit cell radius (a) or up to 1.5 unit cells radius (b) with appropriate intensity scales.

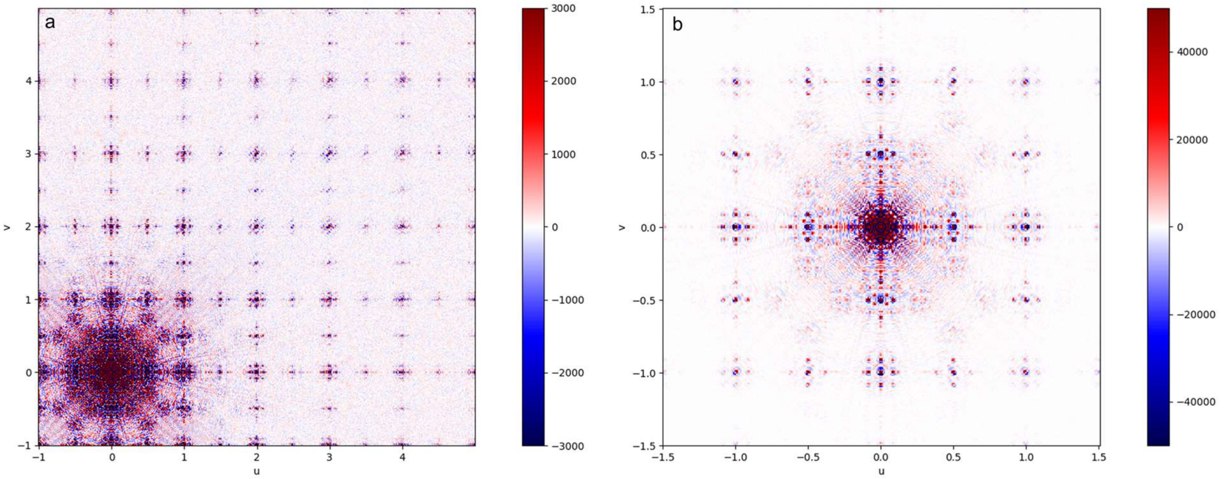

Figure S7.1.8. 3DΔPDF function extracted for cubic c-(4,12)MTBC-Zr_6 crystal 1. The uv0 plane with appropriate scales up to 5 unit cell radius (a) or up to 1.5 unit cells radius (b) with appropriate intensity scales.

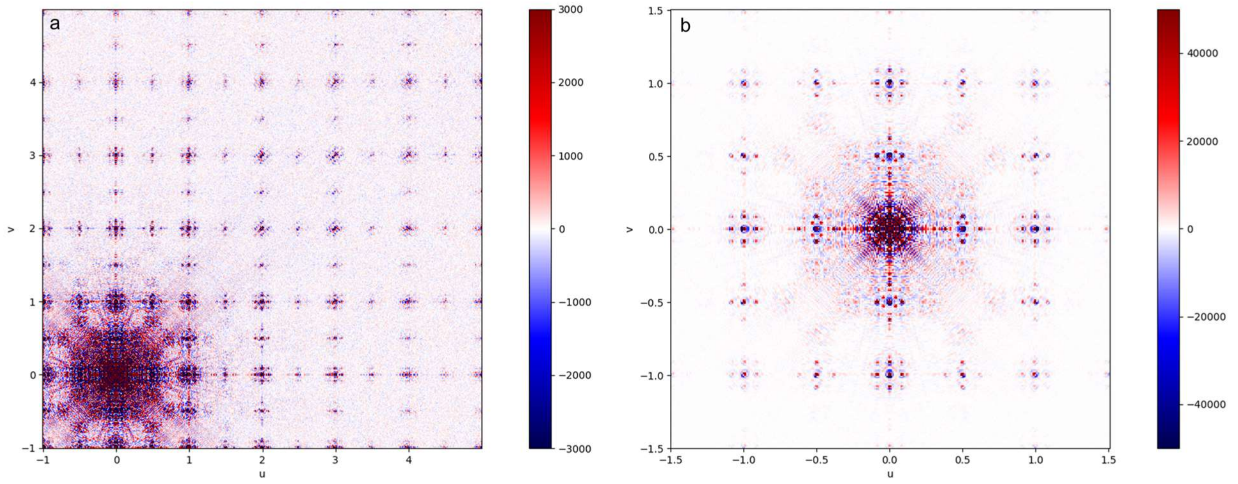

Figure S7.1.9. 3DΔPDF function extracted for cubic c-(4,12)MTBC-Zr_6 crystal 2. The uv0 plane with appropriate scales up to 5 unit cell radius (a) or up to 1.5 unit cells radius (b) with appropriate intensity scales.

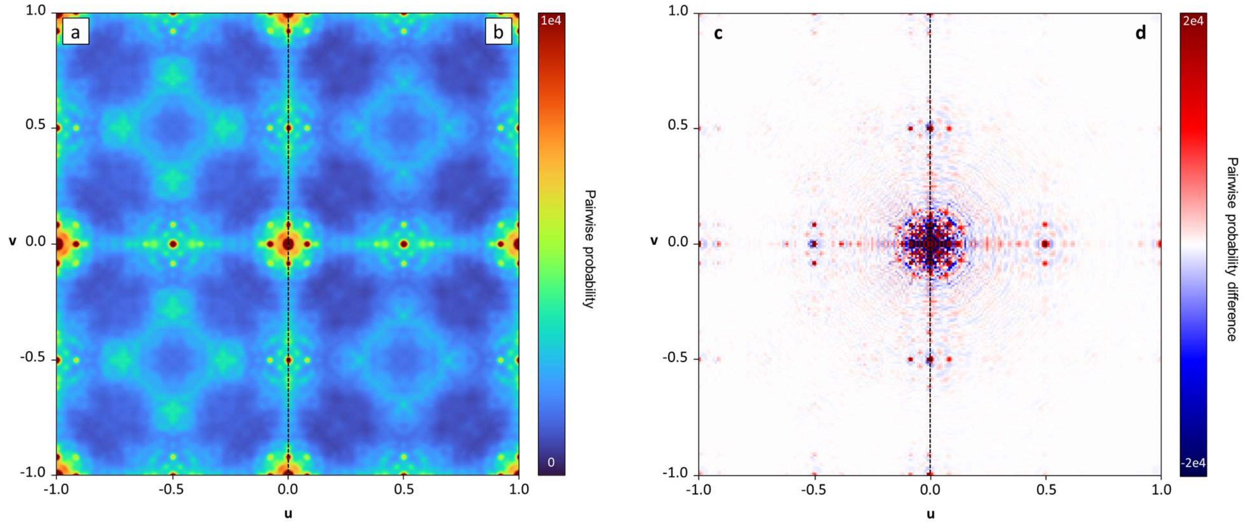

Figure S7.1.10. Left: Patterson function calculated by the software Vesta by using the average structure model of c-(4,12)MTBC-Zr_6 (cubic crystal nr. 1) with and without the Zr atoms belonging to the disordered cluster (a and b respectively). Right, reconstructed 3DΔPDF obtained from c-(4,12)MTBC-Zr_6 crystals with cubic and truncated octahedral morphology (c and d respectively). All the most prominent pairwise probability maxima observed in the 3DΔPDF functions can be related to those found in the Patterson functions regardless of which is considered, a or b. The correlation range is one unit cell length in each of the shown PDF and ΔPDF maps.

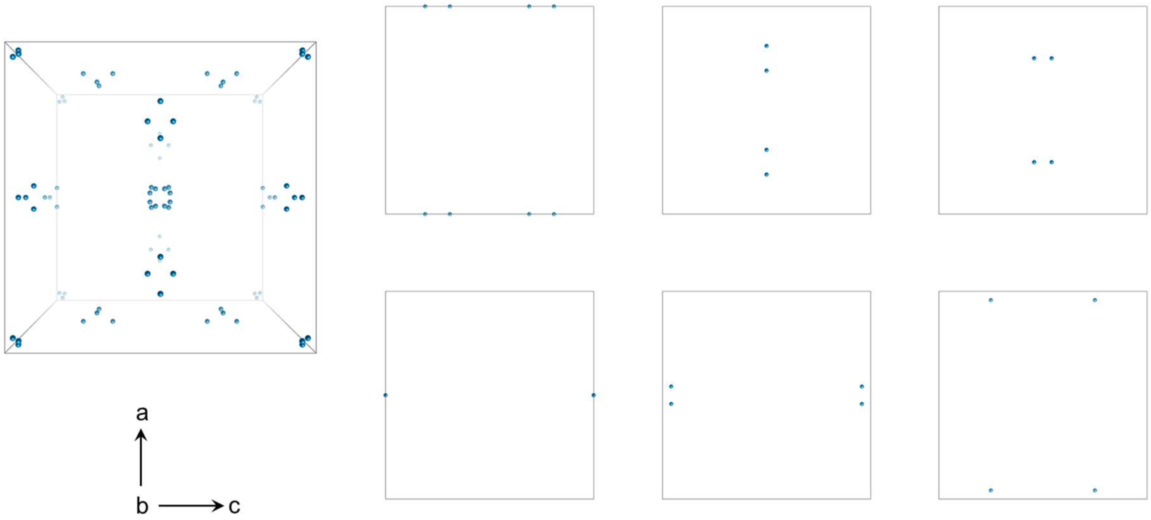

Figure S7.1.11. Unit cell containing every Zr atom of the truncated-octahedral c-(4,12)MTBC-Zr_6 structure (left), and the six types of planes parallel to ab containing Zr atoms, excluding those belonging to the disordered clusters. As can be seen, the variety of possible Zr–Zr interatomic vectors include numerous vectors lying along the main axes, i.e., horizontal or vertical.

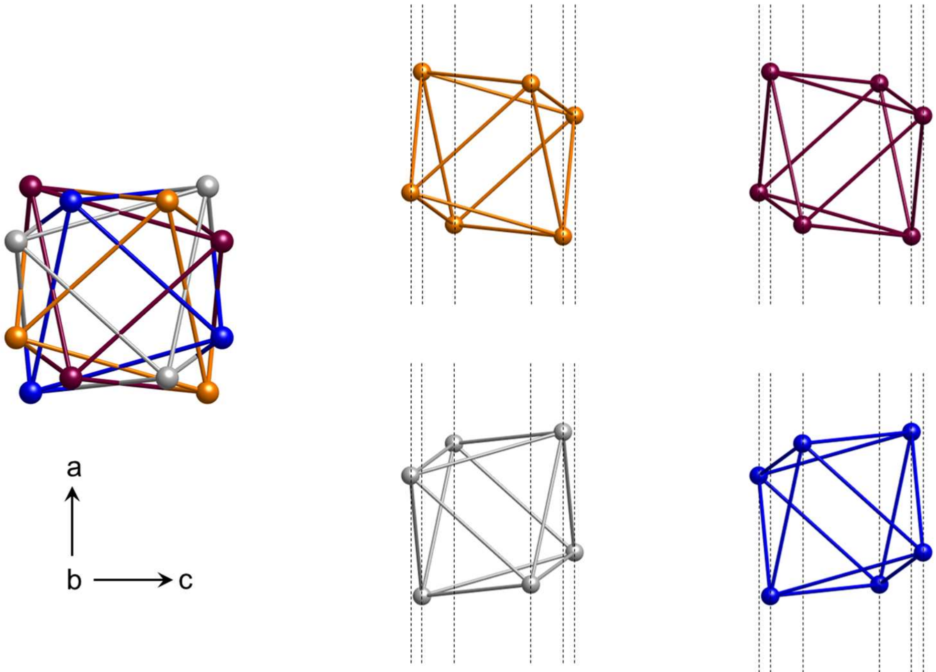

Figure S7.1.12. The absence of Zr–Zr correlations in the main planes (such as the ab plane, and the symmetry equivalent bc and ac) is shown by considering the projection of the disordered cluster of truncated-octahedral c-(4,12)MTBC-Zr_6 along the *c* axis. On the left: complete site with the four different cluster orientation. On the right: each cluster is separately shown, and the ab planes are highlighted as dashed lines. The absence of Zr–Zr correlations is evident from noticing that any dashed line crosses only one Zr atom. Therefore, for any given plane parallel to the main planes (ab, ac or bc) there is only one Zr atom belonging to a single cluster.

S7.2. Diffuse scattering simulations

To simulate the diffuse scattering patterns originating from a disordered c-(4,12)MTBC-Zr_6 crystals with random cluster disorder, we used the program Zürich Oak-Ridge Disorder Simulation (ZODS).[20]

As average structure model, we used the structure refined from the truncated-octahedral crystals, and kept all atomic positions fixed in their average sites. In the disordered sites, 4 mutually exclusive chemical species were defined, corresponding to a $Zr_6O_4(OH)_4$ cluster oriented in one of the four possible alternatives. As we did not define any correlation parameters among those sites, in each site the selection of the cluster orientation to be used was completely random, resulting in what we refer to as “random cluster disorder”. Since no refinement of computed diffuse pattern against experimental one was conducted, we used only an arbitrary number of 100 MC cycles, and simulated 10 single crystal of 20x20x3 unit cells.

For the intensity calculation, we used the Fourier section of the software suite Discus (ver. 6.07.00) and instructed the program to subtract the average structure factor to eliminate Bragg reflections from the computed patterns. Intensities were calculated for the only *hk0* plane, from -58.5 to +58.5 of h and k, 1171 steps for each reciprocal space direction, and using 10 lots of 10x10 unit cells for each of the crystals generated by ZODS. In this way, 10 calculated *hk0* reciprocal space slabs were obtained, and averaged to produce the final pattern shown in the manuscript.

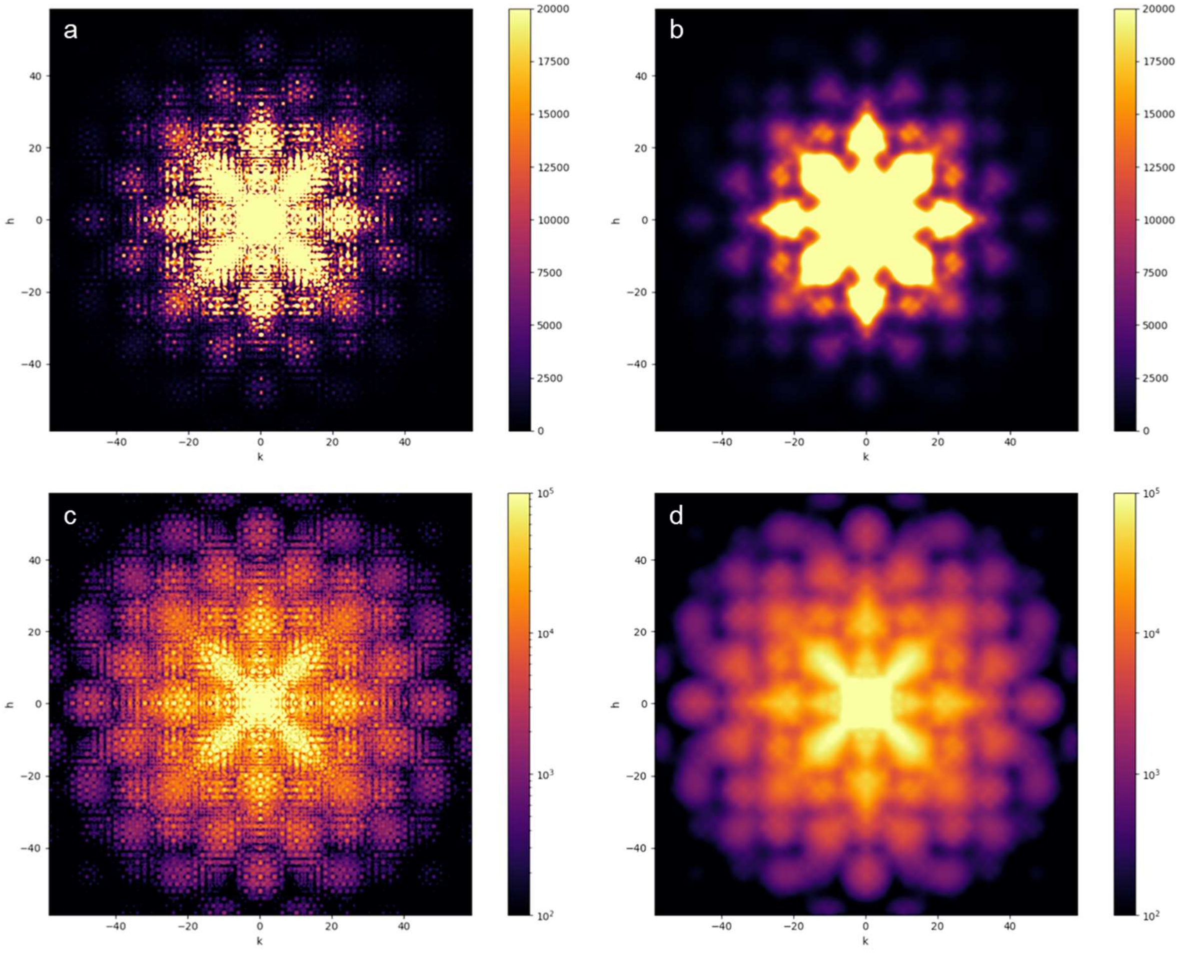

Figure S7.2.1. Simulated *hk*0 plane of the reciprocal space of a **single unit cell** of c-(4,12)MTBC-Zr_6 calculated without considering the disordered clusters in the structural model. From above, pattern in linear intensity scale with and without

the application of a gaussian frequency filter (a and b respectively), and the same patterns displayed with a logarithmic intensity scale (c and d).

S8. Topological analysis of tr-(4-12)MTBC-M_6 and c-(4-12)MTBC-M_6

Topological analysis was conducted by using the programs ToposPro[24] and Systre[25] freely available at https://topospro.com and http://gavrog.org/. The average model containing both disordered fractions was considered, together with two simplified ordered models.

The latter correspond to the models shown on the right side of Figure 6 in violet and turquoise. The underlying nets[26] are defined for octahedral M_6 SBU with tetrahedral ligand **tet**, MTBC and correspond to a general formula (SBU)(**tet**)$_n$. The SBU assume three different geometries: cubic **cub**, icosahedral **ico** or cuboctahedral **cuo.** We use the three letter symbols from polyhedra and nets from RCSR (see http://rcsr.net/).[27]

Table S8.1. Summary of the key compound considered in our analysis, with particular emphasis on the stoichiometric ratio of building blocks. Refcodes refer to the Cambridge Structural Database.

REFCODE (name)	Names used in the text	Simplified formula	Crystal system	Net	Unit cell content
DITJOB (PCN-521)<br>DITJIB (PCN-523)	tetragonal PCN-521/3	(SBU)(**tet**)$_2$	tetragonal	(4,8)-**flu**	(**cub**)$_4$(**tet**)$_8$
EWEDIV, EWEDOB, EWEDUH<br>ARUHUS (NPF-200)<br>ARUJAA (NPF-201)	c-(4,12)MTBC-M_6<br>M= Zr, Hf<br>Zr-MTBC	(SBU)(**tet**)$_3$	cubic	(4,12,12)-**buh**	(**ico**)$_2$(**cuo**)$_6$(**tet**)$_{24}$
This work	tr-(4,12)MTBC-M_6	(SBU)(**tet**)$_3$	Trigonal	Ordered model<br>4,4,12,12-c	(**tet**)$_{18}$(**ico**)$_4$(**cuo**)$_2$
This work	tr-(4,12)MTBC-M_6	(SBU)(**tet**)$_3$	Trigonal	Average model<br>4,4,15,18-c	(**tet**)$_{24}$(15-hedra)$_4$(18-hedra)$_2$

Ordered model: Point symbol for the new tetranodal 4,4,12,12-c net: $(4^6)_9(4^{24}.6^{36}.8^6)(4^{24}.6^{39}.8^3)_2$
Average model: Point symbol for the new tetranodal 4,4,15,18-c net: $(4^6)_{12}(4^{63}.6^{78}.8^{12})(4^{42}.6^{54}.8^9)_2$

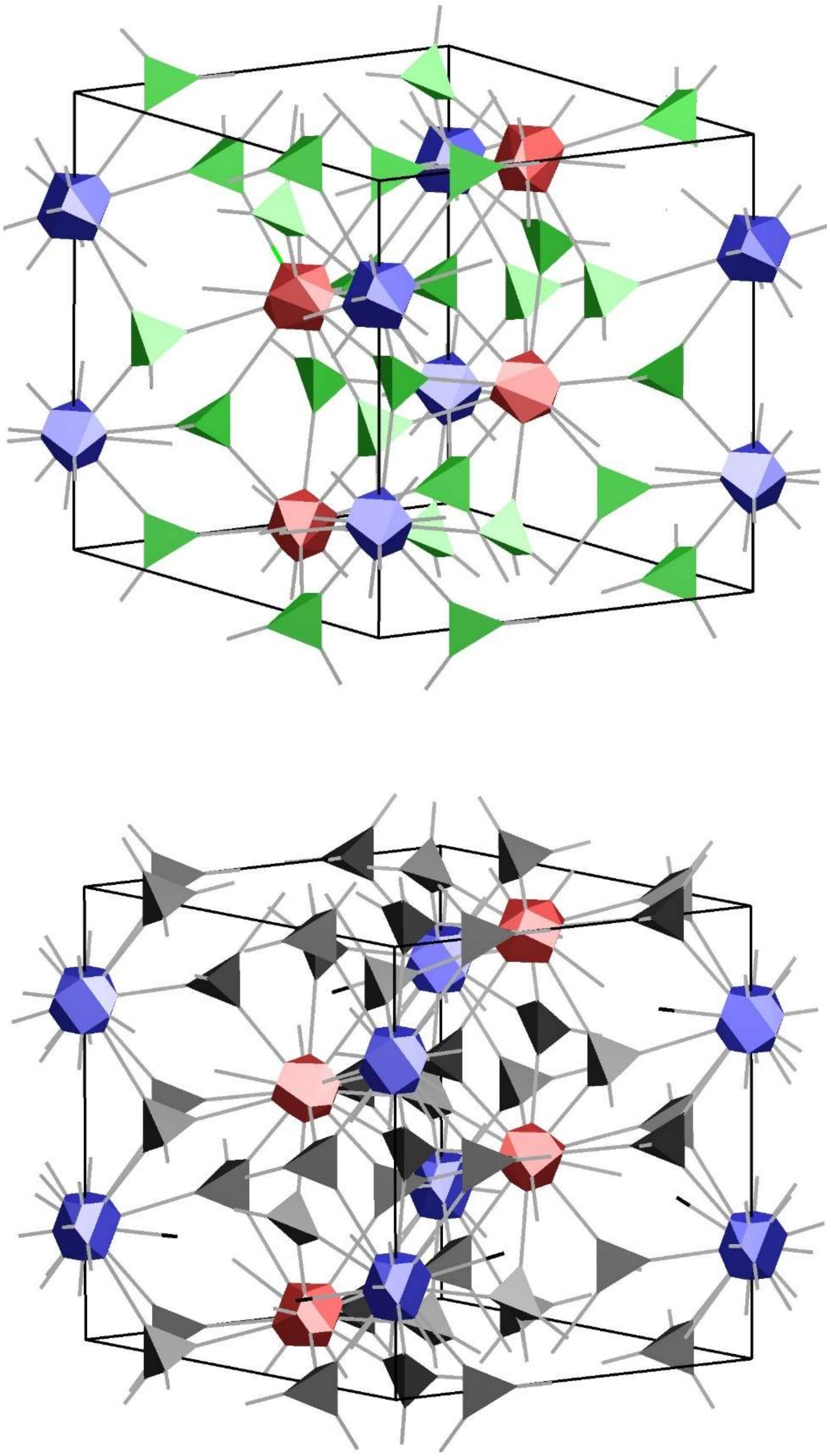

Figure S8.1. Views of the two new nets shown in the augmented form in order to see the different local coordination of the SBU (top, ordered model; bottom, average disordered model). **Blue**: cuboctahedral coordination and derived 18-hedral, **red**: icosahedral and derived 15-hedral, and for the ligand **green** and **black** tetrahedra. The average model shows more **black** tetrahedrons per unit cell respect to the ordered model with **green** tetrahedra.

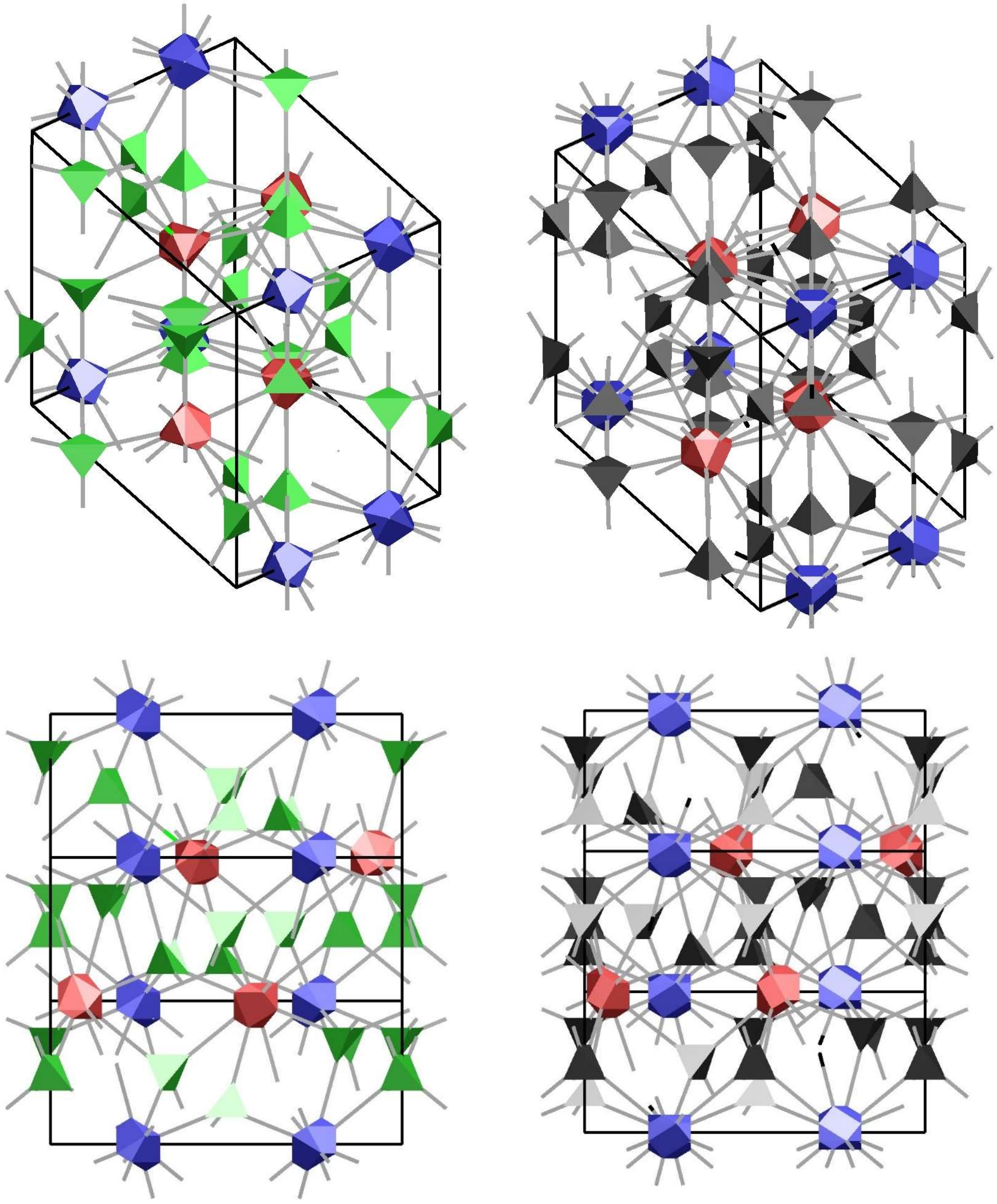

Figure S8.2. Side by side views of the the two new nets along [1 1 1] (top pictures) and [2 0 1] (bottom pictures). The average model (right-hand side) shows more **black** tetrahedra per unit cell respect to the ordered model with **green** tetrahedra.

Table S8.1. Systre files for the two nets, readable by ToposPro. This file format is described here: http://gavrog.org/Systre-Help.html#file_formats

name 4,4,12,12-c **ordered model**<br>cell 2.5298 2.5298 3.0984 90.000 90.000 120.000<br>group P-3c1<br>atom ZA1 12 0.66667 0.33333 0.16667<br>edge ZA1 0.66667 0.08333 -0.08333<br>edge ZA1 0.41667 0.33333 -0.08333<br>edge ZA1 0.91667 0.33333 0.41667<br>edge ZA1 0.41667 0.08333 0.41667<br>edge ZA1 0.33333 -0.08333 0.08333<br>edge ZA1 0.58333 0.66667 0.08333<br>edge ZA1 1.00000 0.25000 0.25000<br>edge ZA1 0.25000 0.00000 0.25000<br>edge ZA1 0.75000 0.75000 0.25000<br>edge ZA1 1.08333 0.41667 0.08333<br>edge ZA1 0.66667 0.58333 0.41667<br>edge ZA1 0.91667 0.58333 -0.08333<br>atom ZB1 12 0.00000 0.00000 0.00000<br>edge ZB1 -0.25000 0.00000 -0.25000<br>edge ZB1 0.25000 0.25000 -0.25000<br>edge ZB1 0.00000 -0.25000 -0.25000<br>edge ZB1 0.25000 0.00000 0.25000<br>edge ZB1 -0.25000 -0.25000 0.25000<br>edge ZB1 0.41667 0.33333 -0.08333<br>edge ZB1 -0.08333 -0.41667 -0.08333<br>edge ZB1 -0.33333 0.08333 -0.08333<br>edge ZB1 -0.41667 -0.33333 0.08333<br>edge ZB1 0.08333 0.41667 0.08333<br>edge ZB1 0.33333 -0.08333 0.08333<br>edge ZB1 0.00000 0.25000 0.25000<br>atom ZC1 4 0.00000 0.25000 0.25000<br>edge ZC1 0.00000 0.00000 0.50000<br>edge ZC1 -0.33333 0.33333 0.16667<br>edge ZC1 0.33333 0.66667 0.33333<br>edge ZC1 0.00000 0.00000 0.00000<br>atom ZD1 4 0.08333 0.41667 0.08333<br>edge ZD1 0.00000 0.00000 0.00000<br>edge ZD1 -0.33333 0.33333 0.16667<br>edge ZD1 0.33333 0.66667 0.33333<br>edge ZD1 0.33333 0.66667 -0.16667<br>end	name 4,4,15,18-c **average model**<br>cell 34.3901 34.3901 49.4392 90.000 90.000 120.000<br>group P-3c1<br>atom ZA1 15 0.33333 0.66667 0.58870<br>edge ZA1 -0.09424 0.33654 0.66027<br>edge ZA1 0.43078 1.09424 0.66027<br>edge ZA1 0.66346 0.56922 0.66027<br>edge ZA1 -0.00094 0.76313 0.51819<br>edge ZA1 0.76407 1.00094 0.51819<br>edge ZA1 0.23687 0.23593 0.51819<br>edge ZA1 0.23593 0.99906 0.48181<br>edge ZA1 0.00094 0.23687 0.48181<br>edge ZA1 0.76313 0.76407 0.48181<br>edge ZA1 0.56922 0.90576 0.33973<br>edge ZA1 0.33654 0.43078 0.33973<br>edge ZA1 0.09424 0.66346 0.33973<br>edge ZA1 0.33654 0.90576 0.83973<br>edge ZA1 0.56922 0.66346 0.83973<br>edge ZA1 0.09424 0.43078 0.83973<br>atom ZB1 4 0.09424 0.66346 0.33973<br>edge ZB1 -0.33333 0.33333 0.41130<br>edge ZB1 0.00000 1.00000 0.25000<br>edge ZB1 0.33333 0.66667 0.58870<br>edge ZB1 0.33333 0.66667 0.08870<br>atom ZC1 4 0.00094 0.23687 0.48181<br>edge ZC1 -0.33333 0.33333 0.41130<br>edge ZC1 0.00000 0.00000 0.25000<br>edge ZC1 0.33333 0.66667 0.58870<br>edge ZC1 0.00000 0.00000 0.75000<br>atom ZD1 18 0.00000 0.00000 0.25000<br>edge ZD1 0.23593 -0.00094 0.48181<br>edge ZD1 0.00094 0.23687 0.48181<br>edge ZD1 0.23687 0.00094 0.01819<br>edge ZD1 -0.23593 -0.23687 0.01819<br>edge ZD1 -0.23687 -0.23593 0.48181<br>edge ZD1 -0.00094 0.23593 0.01819<br>edge ZD1 -0.09424 -0.43078 0.16027<br>edge ZD1 0.09424 -0.33654 0.33973<br>edge ZD1 0.33654 0.43078 0.33973<br>edge ZD1 0.43078 0.33654 0.16027<br>edge ZD1 -0.33654 0.09424 0.16027<br>edge ZD1 -0.43078 -0.09424 0.33973<br>edge ZD1 0.00094 -0.23593 -0.01819<br>edge ZD1 -0.23687 -0.00094 -0.01819<br>edge ZD1 0.23593 0.23687 -0.01819<br>edge ZD1 0.23687 0.23593 0.51819<br>edge ZD1 -0.23593 0.00094 0.51819<br>edge ZD1 -0.00094 -0.23687 0.51819<br>end

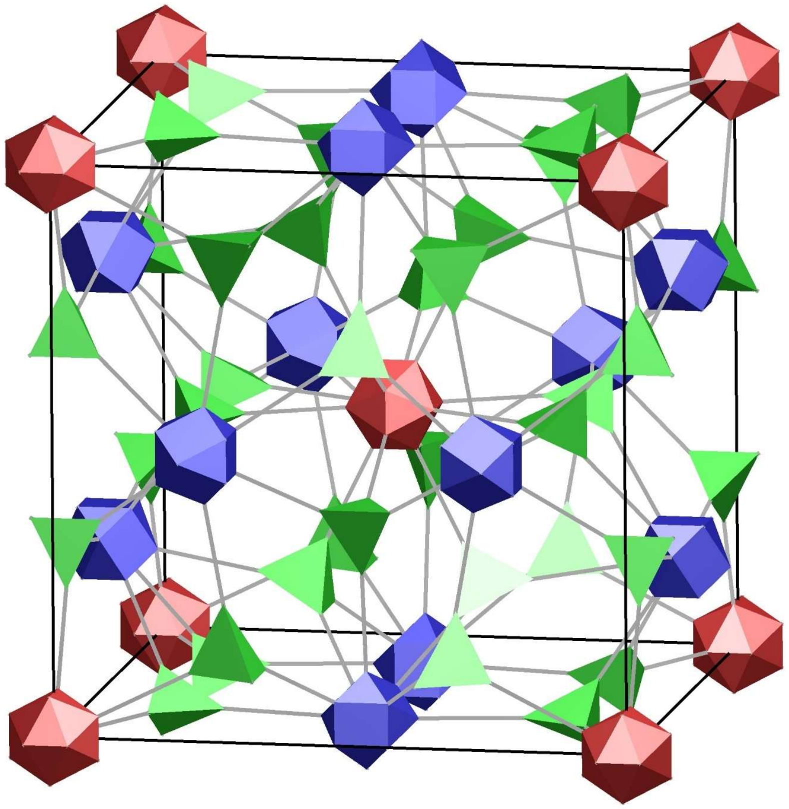

Figure S8.3. The cubic (4,12,12)-**buh** net shown in the augmented form **buh-a** with the cell content: (**ico**)$_2$(**cuo**)$_6$(**tet**)$_{24}$

S9. Remarks on the multiphase behaviour in Zr-MTBC MOFs

Although $Zr_6O_4(OH)_4$ cluster based MOFs have been intensively studied since 2008, the control over MOF phases remains challenging. Oftentimes, multiple MOF topologies with different catalytic activities and gas sorption properties can be built from the same linker and Zr-cluster. [21–24] We identified three MOF phases, cubic, tetragonal, and trigonal Zr-MTBC to form from $ZrCl_4$ and MTBC when using benzoic acid as modulator. The phase composition of the product was found to vary depending on the reaction conditions (Figure S5.1). Changing the reaction time, temperature, concentration of reactant, solvent, and water content during synthesis from $ZrCl_4$ led to several observations worth mentioning, which we list in the following.

(1) Both reaction temperature as well as concentrations of linker and $ZrCl_4$ did not significantly affected the composition of MOF phases formed, all yielding cubic Zr-MTBC as main product. As discussed above, decreasing both MTBC and $ZrCl_4$ concentration, however, remarkably slowed down particle growth (24 h vs 5 d) and yielded c-(4,12)MTBC-Zr_6 crystals with higher crystallinity and a cube-shaped crystal habit instead of a truncated-octahedral one.

(2) Increasing the reaction time, in turn, inversed the phase ratios in the product and more tetragonal and trigonal Zr-MTBC were formed.

(3) Importantly, separation of the supernatant after 24 h reaction and further reaction of the supernatant mostly yielded the tetragonal phases. This suggests, that at the beginning of the reaction cubic and trigonal Zr-MTBC form while tetragonal Zr-MTBC growth later in the reaction.

(4) When employing DMA as solvent instead of DEF, no cubic Zr-MTBC was formed. Similar to the reactions in DEF, longer reaction times favored the formation of tetragonal Zr-MTBC when using DMA as solvent. For instance, reaction from $ZrCl_4$_air yielded a mixture of the trigonal and tetragonal phase after 24 h whereas reaction for 48 h mainly yielded the tetragonal MOF suggesting a phase transformation from the trigonal to tetragonal phase.

To better control reaction kinetics, synthesis of Zr-MTBC MOFs in DMA was further performed using neat $ZrCl_4$_GB under addition of small amounts of water, based on our recent publication on the effect of water during synthesis of porphyritic Zr-MOFs.[1] Microscope images revealed that the amount of water added to the reaction mixture affected the phase composition of the products. Reaction under addition of 4 µL water yielded hexagonal particles whilst synthesis with 8 or 12 µL water yielded mixtures of hexagonal and octahedral particles, characteristic for trigonal and tetragonal Zr-MTBC, respectively. Reaction time affects the phase composition of the product: synthesis with 4 µL water yielded hexagonal particles after 1 d (MOF_DMA_1d_4) and 2 d (MOF_DMA_2d_4) reaction whereas longer reaction times increased the amount of octahedral particles in the product.

Regrettably, PXRD measurements of the products containing hexagonal particles only had poor crystallinity (see Section S4.3), and an unambiguous assignment of peaks to the trigonal phase is not possible. In the absence of phase homogeneity information from PXRD, our conclusions can only be based on the morphological characteristics of the product as hexagonal platelets. Although this morphology has only been observed for the trigonal phase we report, we cannot claim that this is not an additional polymorph of Zr-MTBC.

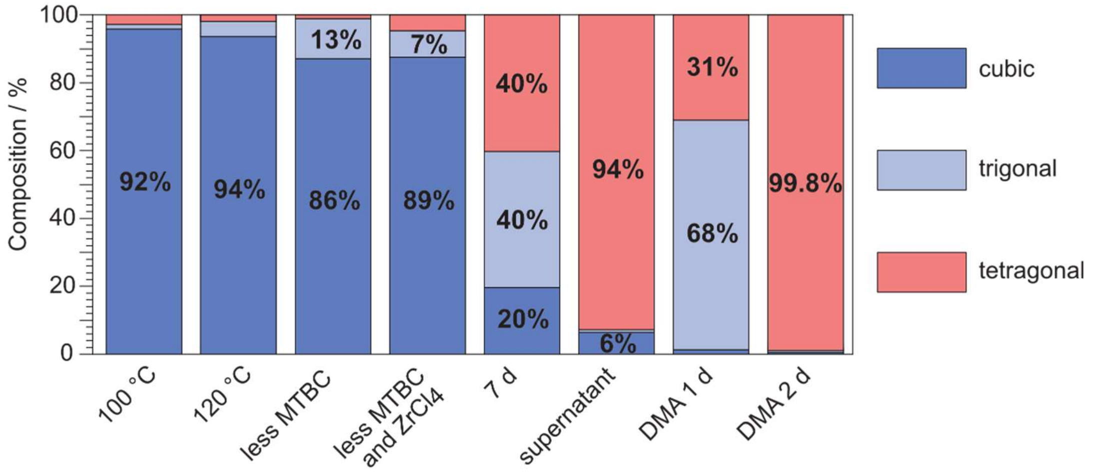

Figure S9.1. Phase compositions of cubic, trigonal, and tetragonal Zr-MTBC depending on the employed reaction conditions.

S10. Nitrogen sorption analysis

Sorption measurements were acquired on a Quantachrome Instruments Autosorb iQ 3 with nitrogen at 77 K. Samples were activated under high vacuum at 120 °C for 12 h before measurement unless stated otherwise. The pore size distribution (PSD) was determined from nitrogen adsorption isotherms using the QSDFT (cylindrical pores, adsorption branch) kernel in carbon for nitrogen at 77 K implemented in the ASiQwin software v 3.01.

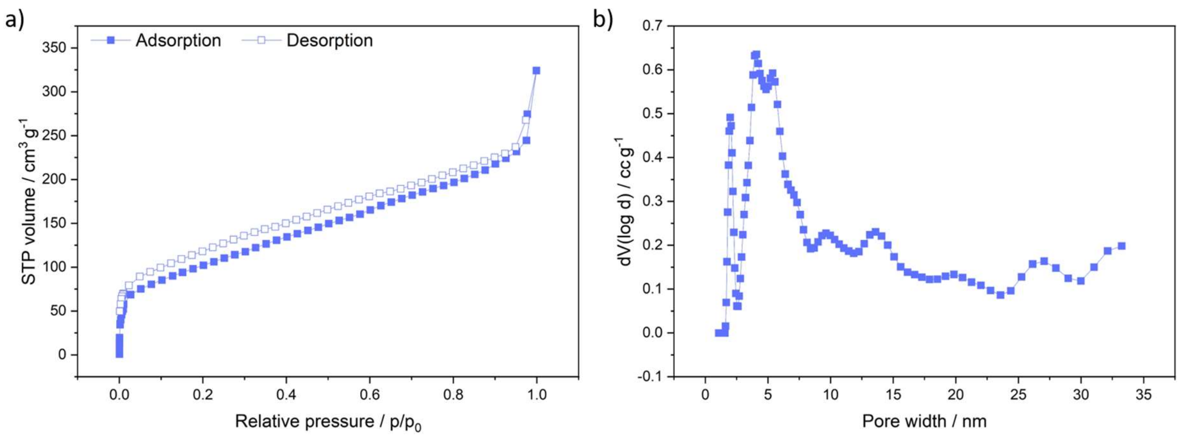

Figure S10.1. a) Nitrogen sorption isotherm (77 K) and b) pore size distribution of product obtained from MOF_DMA_2d_4 and $ZrCl_4$_GB after 24 h reaction time. The Brunauer-Emmett-Teller (BET) surface areas is 368 $m^2\ g^{-1}$.

References

(1) Koschnick, C.; Terban, M. W.; Canossa, S.; Etter, M.; Dinnebier, R. E.; Lotsch, B. V. Influence of Water Content on Speciation and Phase Formation in Zr-Porphyrin-Based MOFs Special Issue on Hygroscopic Materials. *Advanced Materials* **2023**, 2210613. https://doi.org/10.1002/ADMA.202210613.

(2) Ji, P.; Manna, K.; Lin, Z.; Urban, A.; Greene, F. X.; Lan, G.; Lin, W. Single-Site Cobalt Catalysts at New Zr8(M2-O)8(M2-OH)4 Metal-Organic Framework Nodes for Highly Active Hydrogenation of Alkenes, Imines, Carbonyls, and Heterocycles. *J Am Chem Soc* **2016**, *138* (37), 12234–12242.

(3) Schneider, C. A. ; R. W. S. & E. K. W. ImageJ. 2012.

(4) Coelho, A. A. TOPAS and TOPAS-Academic: An Optimization Program Integrating Computer Algebra and Crystallographic Objects Written in C++. *J Appl Crystallogr* **2018**, *51* (1), 210–218.

(5) Chupas, P. J.; Qiu, X.; Hanson, J. C.; Lee, P. L.; Grey, C. P.; Billinge, S. J. L. Rapid-Acquisition Pair Distribution Function (RA-PDF) Analysis. *J Appl Crystallogr* **2003**, *36* (6), 1342–1347.

(6) Ashiotis, G.; Deschildre, A.; Nawaz, Z.; Wright, J. P.; Karkoulis, D.; Picca, F. E.; Kieffer, J. The Fast Azimuthal Integration Python Library: PyFAI. *J Appl Crystallogr* **2015**, *48* (2), 510–519.

(7) Kieffer, J.; Valls, V.; Blanc, N.; Hennig, C. New Tools for Calibrating Diffraction Setups. *J Synchrotron Radiat* **2020**, *27* (2), 558–566.

(8) Billinge, S. J. L.; Farrow, C. L. Towards a Robust Ad Hoc Data Correction Approach That Yields Reliable Atomic Pair Distribution Functions from Powder Diffraction Data. *Journal of Physics: Condensed Matter* **2013**, *25* (45), 454202.

(9) Juhás, P.; Davis, T.; Farrow, C. L.; Billinge, S. J. L. PDFgetX3: A Rapid and Highly Automatable Program for Processing Powder Diffraction Data into Total Scattering Pair Distribution Functions. *J Appl Crystallogr* **2013**, *46* (2), 560–566.

(10) Juhás, P.; Farrow, C. L.; Yang, X.; Knox, K. R.; Billinge, S. J. L. Complex Modeling: A Strategy and Software Program for Combining Multiple Information Sources to Solve Ill Posed Structure and Nanostructure Inverse Problems. *Acta Crystallogr A Found Adv* **2015**, *71* (6), 562–568.

(11) *Oxford Cryosystems Ltd., Oxford, United Kingdom*.

(12) Kabsch, W.; IUCr. XDS. *Acta Crystallographica Section D* **2010**, *66* (2), 125–132.

(13) *Rigaku Oxford Diffraction, (2017), CrysAlisPro Software System, Version 1.171.40.67a, Oxford, UK.*

(14) Dolomanov, O. v.; Bourhis, L. J.; Gildea, R. J.; Howard, J. A. K.; Puschmann, H. OLEX2: A Complete Structure Solution, Refinement and Analysis Program. *J Appl Crystallogr* **2009**, *42* (2), 339–341. https://doi.org/10.1107/S0021889808042726.

(15) Sheldrick, G. M.; IUCr. Crystal Structure Refinement with SHELXL. *Acta Crystallographica Section C* **2015**, *71* (1), 3–8.

(16) Momma, K.; Izumi, F. VESTA 3 for Three-Dimensional Visualization of Crystal, Volumetric and Morphology Data. *J Appl Crystallogr* **2011**, *44* (6), 1272–1276. https://doi.org/10.1107/S0021889811038970.

(17) A. Simonov. Https://Github.Com/Aglie/Meerkat.

(18) Kobas, M.; Weber, T.; Steurer, W. Structural Disorder in the Decagonal Al-Co-Ni. I. Patterson Analysis of Diffuse x-Ray Scattering Data. *Phys Rev B Condens Matter Mater Phys* **2005**, *71* (22), 224205.

(19) Https://Docs.Scipy.Org/Doc/Scipy/Reference/Generated/Scipy.Interpolate.Rbf.Html#. Scipy.Interpolate.Rbf.

(20) Frison, R.; Chodkiewicz, M.; Ahrenberg, L.; Bürgi, H.-B.; Weber, T. ZODS – Zurich Oak Ridge Disorder Simulation Software, University of Zurich, Switzerland. 2020.

(21) Karadeniz, B.; Žilić, D.; Huskić, I.; Germann, L. S.; Fidelli, A. M.; Muratović, S.; Lončarić, I.; Etter, M.; Dinnebier, R. E.; Barišić, D.; Cindro, N.; Islamoglu, T.; Farha, O. K.; Friščić, T.; Užarević, K. Controlling the Polymorphism and Topology Transformation in Porphyrinic Zirconium Metal-Organic Frameworks via Mechanochemistry. *J Am Chem Soc* **2019**, *141* (49), 19214–19220.

(22) Gong, X.; Noh, H.; Gianneschi, N. C.; Farha, O. K. Interrogating Kinetic versus Thermodynamic Topologies of Metal-Organic Frameworks via Combined Transmission Electron Microscopy and X-Ray Diffraction Analysis. *J Am Chem Soc* **2019**, *141* (15), 6146–6151.

(23) Bon, V.; Senkovska, I.; Baburin, I. A.; Kaskel, S. Zr- and Hf-Based Metal-Organic Frameworks: Tracking down the Polymorphism. *Cryst Growth Des* **2013**, *13* (3), 1231–1237.

(24) Bai, Y.; Dou, Y.; Xie, L. H.; Rutledge, W.; Li, J. R.; Zhou, H. C. Zr-Based Metal–Organic Frameworks: Design, Synthesis, Structure, and Applications. *Chem Soc Rev* **2016**, *45* (8), 2327–2367.
